# Varying Projection Quality of Good Local Electric Field Gradients of Monochlorobenzaldehydes

**DOI:** 10.1021/acs.jpca.4c04915

**Published:** 2025-01-17

**Authors:** Robin Dohmen, Sean Arnold, Jessica Garrett, Beate Kempken, Beppo Hartwig, Benjamin Schröder, Pablo Pinacho, Melanie Schnell, Gordon G. Brown, Daniel A. Obenchain

**Affiliations:** † University of Göttingen, Institute for Physical Chemistry, Tammannstraße 6, 37077,Göttingen Germany; ‡ Coker University, Hartsville, South Carolina 29550, United States; § Deutsches Elektronen-Synchrotron DESY, Notkestr. 85, 22607, Hamburg Germany; ∥ Department of Physical Chemistry and Inorganic Chemistry, IU-CINQUIMA University of Valladolid, Paseo Belén 7, Valladolid 47011, Spain; ⊥ Institut für Physikalische Chemie, Christian-Albrechts-Universität zu Kiel, Max-Eyth-Str. 1, 24118, Kiel Germany

## Abstract

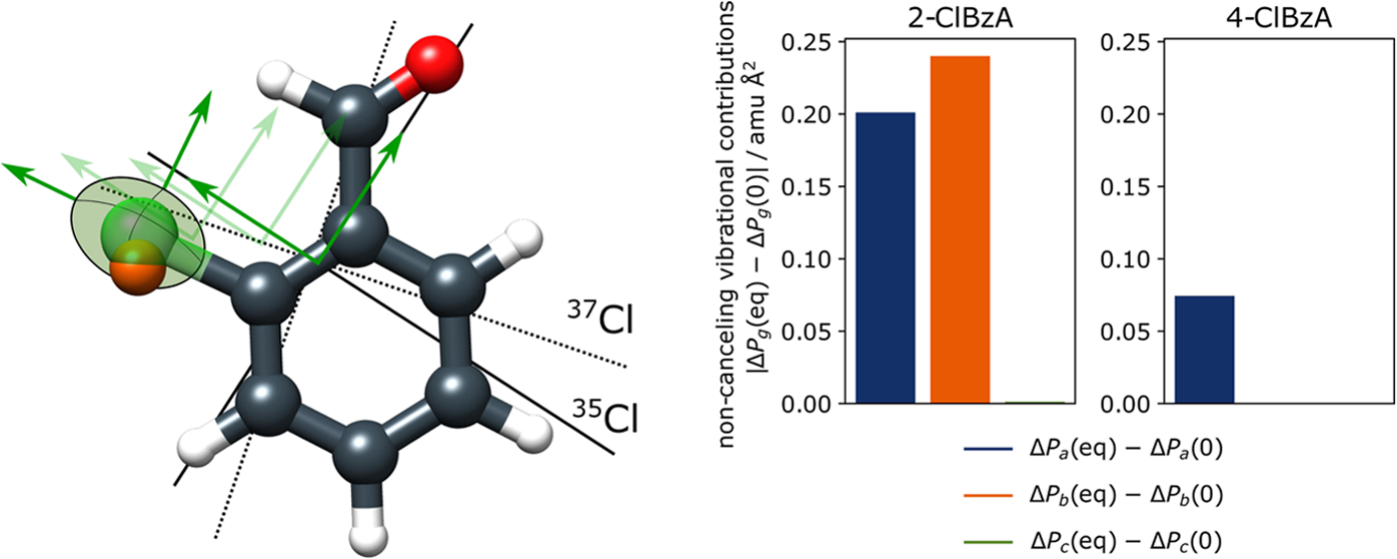

Rotational spectroscopy is an excellent tool for structure determination, which can provide additional insights into local electronic structure by investigating the hyperfine pattern due to nuclear quadrupole coupling. Jet-cooled molecules are good experimental benchmark targets for electronic structure calculations, as they are free of environmental effects. We report the rotational spectra of 2-chlorobenzaldehyde, 3-chlorobenzaldehyde, and 4-chlorobenzaldehyde, including a complete experimental description of the nuclear quadrupole coupling constants, which were previously not experimentally determined. We identified two conformers for 3-chlorobenzaldehyde and one conformer each for 2-chlorobenzaldehyde and 4-chlorobenzaldehyde. Rigorous structure fitting of 4-chlorobenzaldehyde was performed to determine bond lengths for *r*_0_, *r*_s_, *r*_e_^se^, and *r*_*m*_^(1)^ structures. Comparing experimental nuclear quadrupole coupling constants to computational results showed agreement in the nuclear axis system, but the accuracy of the projection into the principal axis system decreases in near-oblate 2-chlorobenzaldehyde. The experimental angle Θ_az_ = 19.16° between the principal *a*-axis and nuclear *z*-axis is larger than predicted by multiple computational methods by ≥4°. It is attributed to the high sensitivity of 2-chlorobenzaldehyde to low-energy vibrational contributions.

## Introduction

Rotational spectroscopy provides a direct link between spectroscopy and geometry through the moments of inertia of a rotating molecule, thereby providing information on bond lengths and angles.^1,2^ Additional angular momentum coupling leads to secondary spectroscopic constants, which can be experimentally determined.^3^ A common example is nuclear quadrupole coupling (NQC) caused by the interaction of the electric nuclear quadrupole moment with the rotational angular momentum. Atoms with a quadrupolar nucleus serve as spectroscopic probes of the local electronic environment. The observed hyperfine splitting is dependent on the electric quadrupole moment of the coupling nucleus and the electric field gradient (EFG) surrounding the nucleus. Hence, the NQC provides experimental insight into the electronic environment, which is a primary parameter for quantum chemical calculations.^4−8^ In addition, the nuclear quadrupole coupling constants (NQCCs) are experimentally determined in the principal axis system (PAS) of the moments of inertia; hence, they are also dependent on the geometry through a projection into that axis system.^1^

For rotational spectroscopy, theoretical predictions are common practice for structural interpretation and the assignment of spectroscopic data. Gas-phase rotational constants and NQCCs are parameters that are accessible to both theory and experiment, which makes them ideal target parameters for benchmarking.^4^ The NQCCs become harder to predict for theory when moving to larger nuclei with a high number of electrons, such as bromine or iodine,^9^ or in cases where the geometry is more flexible, such as weakly bound dimers.^10^ To ensure an accurate description of increasingly complex systems by theory, theory–theory benchmarking should be supplemented with experimental data because even isoelectronic systems can show significant differences, as we demonstrate in this work. To cross-validate predictions provided by theory, experimental benchmark data from both the primary structures and secondary electronic parameters are required.

In this work, we present rotational spectroscopic data for 2-chlorobenzaldehyde (2-ClBzA), two conformers of 3-chlorobenzaldehyde (3-ClBzA), and 4-chlorobenzaldehyde (4-ClBzA). This includes rotational constants, a full description of the NQC tensor for chlorine, and ^37^Cl isotope data for all conformers. Thereby we are expanding on previous structural investigations of 4-ClBzA.^11^ We present a detailed structural analysis of 4-ClBzA by conducting structural fits for the vibrational ground-state structure and semiexperimental equilibrium geometry fits. In addition, we perform extensive computational benchmarking of structures as well as NQCCs with 25 computational methods. This work expands the series of rotational studies of fluorobenzaldehydes^12,13^ by targeting heavier homologues and is a stepping stone for the investigation of bromo- and iodobenzaldehydes. It also serves as a foundation for future studies investigating weakly bound dimers containing halogenbenzaldehydes with nuclear quadrupole moments.

## Experimental Details

In this collaborative work, four instruments participated in the spectroscopic data collection process. Rotational spectra of 2-ClBzA and 3-ClBzA were recorded in a jet-cooled chirped-pulse Fourier transform microwave spectrometer in Hartsville with a spectral range of 8–18.5 GHz, as described previously.^14^ A sample of 2-ClBzA was purchased from Sigma-Aldrich and heated to 85 °C for seeding into a supersonic-jet expansion using a Ne/He admixture (80/20%) at a backing pressure of 3.5 bar. The heating and expansion conditions for 3-ClBzA were the same as those for 2-ClBzA.

A spectrum of 4-ClBzA (97% chemical purity, Sigma-Aldrich) was measured on a jet-cooled chirped-pulse Fourier transform microwave spectrometer in Hamburg (COMPACT) in a spectral range of 2–8 GHz.^15^ The solid was placed into the sample holder at the nozzle and heated to 50 °C. The compound-gas mixture was expanded into a vacuum chamber at an absolute pressure of 3.5 bar using a mixture of 95% Ne/5% D_2_ as a carrier gas. Deuterium was seeded into the expansion to observe complex formation between it and 4-ClBzA, but the discussion in this work focuses exclusively on the monomer.

Several lines from 2-ClBzA (97% chemical purity, Sigma-Aldrich) and 3-ClBzA (99% chemical purity, Sigma-Aldrich) were remeasured with a Balle–Flygare type^16^ cavity Fourier transform microwave spectrometer (QCUMBER) to further resolve hyperfine splittings and improve the determined constants. The instrument was originally built in Kiel^17^ and updated when moved to Göttingen. The updated circuit diagram is in the Supporting Information.^18^ Data between 8 and 18 GHz was recorded by heating the reservoir to 60 °C and seeding into neon-based supersonic-jet expansion at an absolute pressure of 1.6 bar. A few preliminary cavity measurements were done at the Balle–Flygare type cavity Fourier transform microwave spectrometer COBRA in Hannover.^19^ All lines measured at the COBRA instead of the QCUMBER are annotated in the lin file in the supplement on Gro.Data.^18^

Supplementary vibrational measurements were conducted at the *curry* setup, a jet-cooled Raman spectrometer. The setup has been described previously and therefore will not be discussed in this publication.^20−23^

All experimental spectroscopic constants were fitted by utilizing Pickett’s SPCAT/SPFIT^24^ software and Kisiel’s AABS package as a graphical interface.^25,26^ A semirigid, S-reduced Hamiltonian in the I^*r*^ representation was used to fit spectroscopic constants.

All figures presented in this work were rendered utilizing 119 PMIFST,^25^ gle,^27^ Origin 2020,^28^ Python 3.8,^29−33^ and UCSF Chimera.^34^

## Computational Details

For all conformers, equilibrium structure optimizations were performed using the ORCA program package version 5.0.4.^35−37^ The choice of methods was also based on comparability with previous works.^10^ Among the DFT methods, B3LYP^38−42^ was used, including D3^43,44^ and D4^45−48^ dispersion-corrected extensions with Becke–Johnson damping.^44^ Furthermore, the double hybrid functional B2PLYP^49^ with an auxiliary basis set,^50^ BP86,^38,51,52^ a Generalized Gradient Approximation (GGA) functional, and B97M-D3BJ,^53,54^ which is a meta-GGA functional, were chosen for additional calculations. For the DFT calculations, the Karlsruhe def2-TZVPPD,^55−58^ x2c-TZVPP-2c,^59^ and x2c-TZVPP-s^60^ basis sets were chosen. While those basis sets are originally parametrized for 2-component methods, this is not implemented in ORCA. For a relativistic treatment of the latter two, the Douglas–Kroll–Hess method, as implemented in ORCA, was used.^61−63^ A comparison between relativistic and nonrelativistic basis sets has been proven to be important, for e.g., copper, bromine, and iodine.^64−67^ We extended that comparison to chlorine to determine the extent of relativistic effects on this lighter atom. Further *ab initio* calculations were carried out using the MP2^68^ method with a 6-311++G(d,p)^69−74^ and 6-311++G(2d,2p) Pople basis set. While MP2 is susceptible to errors in aromatic systems, the addition of diffuse functions to the basis prevents a loss of planarity during optimization and hence makes MP2 an interesting test case.^75,76^

To fit the semiexperimental equilibrium structures, second-order vibrational perturbation theory (VPT2)^77−80^ calculations were performed with Gaussian 16 Rev C.01^81^ at the B3LYP-D3/def2-TZVPP level. These were carried out for the main 4-ClBzA isotopolouge and the eight monosubstituted isotopologues containing ^37^Cl and ^13^C. To determine the distortion coefficients using an I^*r*^ representation for the Watson S-reduction, the values for τ_gggg_^′^ yielded by Gaussian were converted to quartic distortion coefficients of the reduced rotational Hamiltonian in accordance with Watson as described in the Gordy & Cook monograph.^1,82,83^

In addition, a relaxed surface scan was carried out with Gaussian for all three structural isomers to determine the barrier of formyl rotation. The dihedral angle of the formyl group relative to the benzyl ring was set while all other coordinates were relaxed in the molecule. These calculations were carried out at the MP2/6-311++G(d,p), MP2/6-311++G(2d,2p), and B3LYP-D3/def2-TZVPP levels of theory.

## Results and Discussion

**Figure 1:**
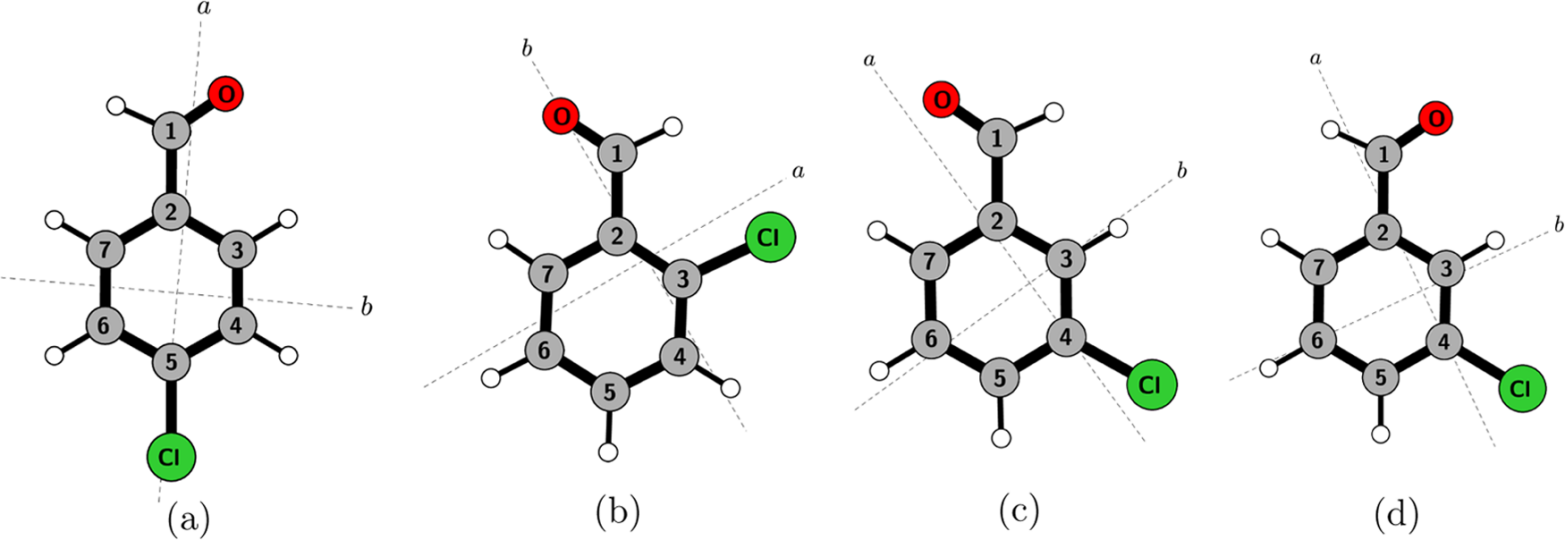
Four main structural isomers of chlorobenzaldehyde (a) 4-ClBzA, (b) *trans*-2-ClBzA, (c) *trans*-3-ClBzA, and (d) *cis*-3-ClBzA with numbered carbon atoms.

The three structural isomers are presented and sorted by decreasing the distance between the substituents on the aromatic ring. Only a single conformer exists for 4-ClBzA, which is shown in Figure 1, but the full set of carbon isotopologues observed in the experimental spectrum allows for an in-depth structural analysis. For 3-ClBzA, two isomers were observed and will be discussed. Only one conformer was observed for 2-ClBzA, and the discussion of its spectroscopic constants leads to benchmarking of computational values for the nuclear quadrupole coupling constants with experimental results.

### 4-Chlorobenzaldehyde

For 4-ClBzA, the experimental spectroscopic constants of the main isotopologue (^35^Cl) and eight singly substituted isotopologues (^37^Cl and ^13^C) are listed in Table 1. The small inertial defect of the ^35^Cl main isotopologue Δ = *I*_C_ – *I*_A_ – *I*_B_ = −0.2471(1) μÅ^2^ is consistent with a planar ground-state structure. The nonzero value of Δ arises from out-of-plane zero-point vibrations.^1^ A full list of assigned transitions can be found in the supplement.^18^ Due to symmetry, 4-ClBzA possesses only one structural isomer, as depicted in Figure 1.

**Table 1:** Experimental Spectroscopic Constants of 4-ClBzA and Its Eight Singly Substituted Isotopologues Compared to VPT2 Results at the B3LYP-D3(BJ)/def2-TZVPP Level of Theory^a^

	4-^35^ClBzA				
	B3LYP-D3/def2-TZVPP	4-^35^ClBzA (main, exp)	^37^Cl (exp)	^13^C1 (exp)	^13^C2 (exp)
*A*/MHz	5100.8	5056.9489(5)	5054.6383(7)	5050.183(1)	5054.457(2)
*B*/MHz	690.86	691.99234(8)	675.5223(1)	683.8799(2)	689.9627(2)
*C*/MHz	608.45	608.83587(8)	596.0178(1)	602.4523(2)	607.2287(2)
χ*_aa_*/MHz	–74.21	–70.696(1)	–55.737(2)	–70.73(1)	–70.70(2)
χ_*bb*–*cc*_/ MHz	8.40	5.988(4)	4.744(4)	5.99(2)	5.99(2)
|χ*_ab_*|/ MHz	10.14	9.5(1)	7.3(4)	10.5(8)	6(2)
*D*_*J*_/kHz	0.011	0.0086(9)	0.013(1)	[0.0086]	[0.0086]
*D*_JK_/kHz	–0.048	–0.042(6)	–0.06(1)	[−0.042]	[−0.042]
*D*_K_/kHz	1.38	1.6(1)	1.4(1)	[1.6]	[1.6]
*d*_1_/kHz	–0.0017	–0.0013(2)	[−0.0013]	[−0.0013]	[−0.0013]
no. lines		277	233	67	47
RMS/kHz		3.9	4.2	9.4	7.6
	^13^C3 (exp)	^13^C4 (exp)	^13^C5 (exp)	^13^C6 (exp)	^13^C7 (exp)
*A*/MHz	5003.234(1)	4989.951(1)	5057.170(1)	4977.537(2)	4963.620(1)
*B*/MHz	691.2701(2)	691.7725(2)	690.4644(1)	691.5225(2)	691.5707(2)
*C*/ MHz	607.4933(2)	607.6841(2)	607.6571(2)	607.3058(2)	607.1345(2)
χ*_aa_*/MHz	–70.66(1)	–70.73(1)	–70.70(1)	–70.65(1)	–70.73(1)
χ_*bb*–*cc*_/MHz	5.97(2)	5.96(2)	6.01(2)	5.93(2)	5.98(2)
|χ*_ab_*|/ MHz	9(1)	7(1)	10(1)	7(1)	8(1)
*D*_J_/kHz	[0.0086]	[0.0086]	[0.0086]	[0.0086]	[0.0086]
*D*_JK_/kHz	[−0.042]	[−0.042]	[−0.042]	[−0.042]	[−0.042]
*D*_K_/kHz	[1.6]	[1.6]	[1.6]	[1.6]	[1.6]
*d*_1_/kHz	[−0.0013]	[−0.0013]	[−0.0013]	[−0.0013]	[−0.0013]
no. lines	60	70	71	68	68
RMS/kHz	8.3	9.0	8.5	11	10

a The fit uses an *S*-reduced semirigid rotor Hamiltonian where *d*_2_ was excluded as its inclusion did not improve the fit and has no significant influence on other fitting parameters. Parameters in square brackets were fixed at the value from the main isotopologue. For the carbon isotopologues, the centrifugal distortion parameters are set to the main isotopologue values. Each isotopic species is labeled by the singly substituted atom (see Figure 1); all other atoms of the molecule are the naturally most abundant isotope.

Structural fitting of 4-ClBzA was performed based on experimental rotational constants of the main isotopologue species and all singly substituted isotopologues measured in natural abundance. The nomenclature for the carbon atom skeleton used is shown in Figure 1. For a first estimation of the heavy atom backbone, determination of the absolute atomic coordinates (*r*_s_) through Kraitchman’s equations for isotopic substitution was performed.^84^ For this purpose, the molecule was treated as planar in Kisiel’s KRA program,^25,26^ which implements the Kraitchman’s equation, yielding absolute atomic coordinates for the chlorine and carbon atoms of the molecule. Through cross-referencing with electronic structure calculations, the sign of the absolute atomic coordinates was inferred for the carbon backbone and chlorine atom. The supporting material contains the atomic coordinates and their respective errors.

An experimental ground-state structure *r*_0_ was fitted for 4-ClBzA using a least-squares fitting algorithm as implemented in Kisiel’s STRFIT program^25,85^ and using the ground-state B3LYP-D3(BJ)/def2-TZVPP structure as input geometry. As it is a planar molecule, each set of rotational constants for an isotopologue provides two degrees of freedom for the least-squares fit. The convergence of the ground-state structure is poor for a planar molecule such as 4-ClBzA,^86^ resulting in large errors for the bond distances and angles. This is caused by the influence of ground-state vibrations, as indicated by the nonzero experimental inertial defect. To mitigate the influence of rovibrational contributions on the fit, which increases with the moment of inertia, only rotational constants *A* and *B* were used for the *r*_0_ fit, resulting in a higher precision. For example, *r*_0_ was also determined by fitting only the rotational constants *A* and *C* or *B* and *C*, respectively, which yielded larger errors, as expected. The *r*_0,*AB*_ ground-state structure is listed in Table 2; all further ground-state structure calculations are included in the supplement.^18^ The ground-state structure obtained from microwave spectroscopy agrees with the structural analysis of 4-ClBzA presented in the literature.^11^ Previous structural analysis was conducted through a combination of gas electron diffraction and microwave spectroscopy, taking the experimental uncertainty of both experiments into account.

**Table 2:** Structural Fitting Parameters for the Substitution Structure *r*_s_, the Ground-State Effective Structure *r*_0_, the Mass-Dependent Structure *r*_*m*_^(1)^, and the Semiexperimental Equilibrium Structure *r*_e_^se^ (B3LYP-D3(BJ)/def2-TZVPP) for 4-ClBzA^a^^11^

parameter	*r*_s_	*r*_0,*AB*_	*r*_*m*_^(1)^	*r*_e_^se^	GED + MW^11^
*R*(C1 – C2)/Å	1.487(2)	1.482(7)	1.486(2)	1.477(4)	1.482(10)
*R*(C2 – C3)/Å	1.393(8)	1.402(30)	1.407(7)	1.396(20)	1.404(1)
*R*(C3 – C4)/Å	1.362(5)	1.382(19)	1.376(6)	1.381(14)	1.392(1)
*R*(C4 – C5)/Å	1.35(2)	1.396(56)	1.396(4)	1.393(37)	1.400(1)
*R*(C5 – C6)/Å	1.45(2)	1.396(66)	1.393(3)	1.391(45)	1.394(1)
*R*(C6 – C7)/Å	1.378(6)	1.392(21)	1.387(6)	1.379(15)	1.398(1)
*R*(C5 – Cl)/Å	1.736(5)	1.735(9)	1.737(2)	1.730(6)	1.734(3)
*A*(O – C1 – C2)/deg		124.7(17)	125.3(5)	124.4(11)	125.5(12)
*A*(C1 – C2 – C3)/deg	120.9(5)	120.5(20)	120.0(6)	120.8(15)	
*A*(C2 – C3 – C4)/deg	120.4(3)	120.1(9)	120.0(2)	120.6(6)	121.8(12)
*A*(C3 – C4 – C5)/deg	120.8(6)	119.0(17)	119.1(1)	118.7(12)	117.6(9)
*A*(C4 – C5 – C6)/deg	120.9(15)	122.0(8)	122.0(2)	121.5(5)	121.0(5)
*A*(C5 – C6 – C7)/deg	117.4(5)	118.4(14)	118.4(2)	119.1(9)	121.2(9)
*A*(C4 – C5 – Cl)/deg	123.3(11)	119.3(45)		118.6(31)	119.5(5)^b^
*c_g_*			0.0188(3)		
σ_fit_/uÅ^2^		0.015	0.0056	0.013	

a Additionally, the structure is compared to the values obtained from combined gas electron diffraction and microwave spectroscopic data from Møllendal et al.^11^

b In ref (11), the angle *A*(C6 – C5 – Cl) was fitted, which was transformed here for consistency.

Inherently, fitting an *r*_0_ structure from the experimental ground-state rotational constants is flawed as it assumes a rigid rotor model and disregards rovibrational contributions. A mass-dependent, semiempirical *r*_*m*_^(1)^ fit improves upon a ground-state *r*_0_ fit by considering rovibrational contributions through an isotopologue-independent parameter, *c*_*g*_, where *g* is the principal axis (*a*, *b*, *c*), which scales the moment of inertia.^2,86^
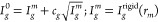
1Each experimental moment of inertia *I*_*g*_^0^ is corrected by the parameter *c*_*g*_ and the rovibrationally corrected moment of inertia *I*_*g*_^*m*^ is utilized to calculate *r*_*m*_^(1)^. For 4-ClBzA, the addition of separate scaling factors *c*_*g*_(*I*_*g*_^*m*^) for each moment of inertia was considered, but the addition of more fitting parameters caused a destabilization of the system due to the number of fitting parameters; therefore, the scaling factor was held constant for all moments of inertia, *c*_*g*_ = *c*_*a*_ = *c*_*c*_.

Finally, a semiexperimental equilibrium structure *r*_e_^se^ was derived from anharmonic ground-state calculations at the B3LYP-D3(BJ)/def2-TZVPP level performed with Gaussian. For this purpose, the first-order vibration–rotation interaction constants α_*i*_^*g*^ of each isotopologue and each vibration *i* were computed, and the ground-state rotational constants were corrected for the vibrational contributions according to  for *A*_e_^se^ and analogously for *B*_e_^se^ and *C*_e_^se^.^86^ The vibration–rotation interaction constants are derived, and the resulting corrected semiexperimental rotational constants (*A*_e_^se^, *B*_e_^se^, and *C*_e_^se^) are then used to perform a least-squares fit. The semiexperimental equilibrium structures can be compared directly to the theoretical results of equilibrium geometry optimizations as well as the other experimental structures.

**Figure 2:**
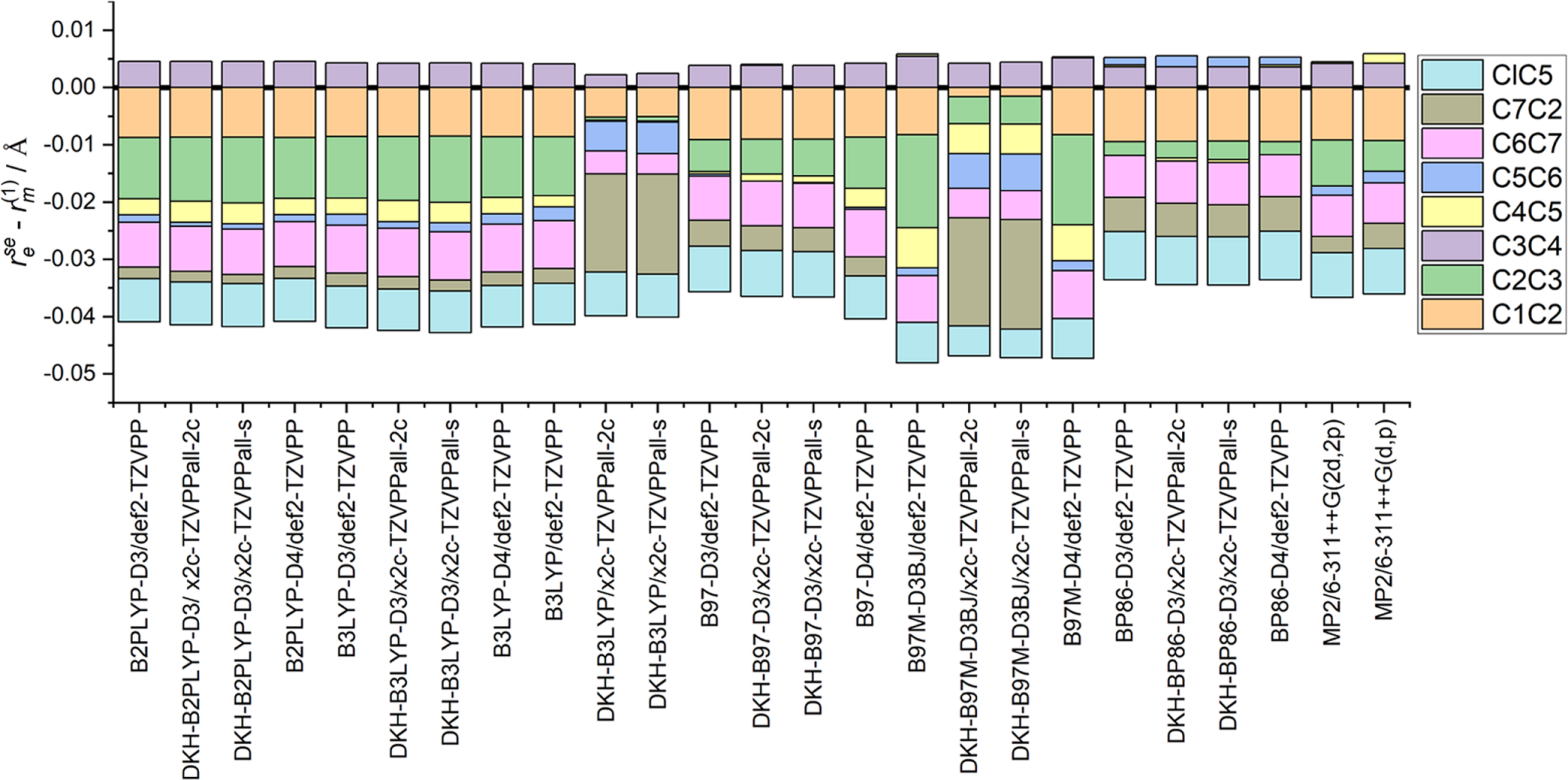
Comparison of *r*_*m*_^(1)^ structures of 4-ClBzA with starting geometries at different levels of theory compared against a *r*_e_^se^ structure that was obtained from a B3LYP-D3/def2-TZVPP calculation. The carbon–carbon bond lengths from each *r*_*m*_^(1)^ structure are subtracted from the bond lengths of the *r*_e_^se^ structure to compare the *r*_*m*_^(1)^ against each other, as well as against semiexperimental equilibrium bond lengths.

To compare theory with experiment, 25 levels of theory described above provided optimized equilibrium geometries as starting structures for a least-squares fitting routine to determine the *r*_*m*_^(1)^ structures of 4-ClBzA. All of the *r*_*m*_^(1)^ least-squares fits converged successfully, with the exception of those using a starting geometry provided by B3LYP and B97M-D3BJ methods combined with any tested x2c basis sets. The convergence issue is reflected in Figure 2, as the C7C2 bond length greatly diverges compared to *r*_*m*_^(1)^ structures by using different starting structures. As this is a cyclic system, it is impossible to define all six carbon–carbon bonds in the ring as fitting parameters, leading to an overestimation of the C7C2 bond by 0.015 Å in these poorly converged *r*_*m*_^(1)^ structures. If the ring definition for the fit is changed, the error shifts to another bond that is not directly treated in the least-squares fit. To maintain comparability between systems, all had to use the same internal coordinate definition. The divergence of the fit is likely caused by the other internal parameters kept constant during the fitting process. Both B3LYP and B97M-D3BJ overestimate the experimental *A* rotational constant by 1.8 and 2.4%, respectively. The latter is beyond the expectation of −1(±1) % deviation when comparing ground-state rotational constants of monomers with equilibrium rotational constants.^87^ However, other calculations such as MP2/6-311++G(d,p) display a similar disparity between experimental and equilibrium rotational constants but lead to a successful convergence of *r*_*m*_^(1)^ structural fits. Hence, the equilibrium rotational constants alone are an insufficient indicator if the geometry is a suitable starting structure for a mass-dependent structure fit.

Excluding the nonconverged structures, regardless of the method, all geometries successfully converged to a common *r*_*m*_^(1)^ geometry as the minimum of the hypersurface. When comparing *r*_e_ structures with the *r*_e_^se^ structure that was obtained from a B3LYP-D3/def2-TZVPP calculation, *r*_e_ bond lengths predicted by B97M are systematically larger than those for the B3LYP *r*_e_^se^ structure, while BP86 and MP2 predict shorter *r*_e_ bond lengths. Details can be found in Supporting Figure S3.^18^

The starting geometry for the least-squares fitting routine has a small impact on the obtained bond lengths but is within the 1σ confidence interval of the structure fit for every method used (Figure 2). Therefore, the choice of starting geometry has a minimal effect on the final result for the *r*_*m*_^(1)^ bond lengths. As expected, the bond angles present in the carbon framework have a higher relative uncertainty compared to the bond lengths. The highest discrepancy between different fits for the angle of C1C2C3 is between B97M-D4/def2-TZVPP and BP86-D4/def2-TZVPP, at 1.1°, which is within the 2σ interval of the least-squares fitting routine.

### 3-Chlorobenzaldehyde

For 3-ClBzA, both the *cis* (*c*) and the *trans* (*t*) isomers, as shown in Figure 1, were observed spectroscopically under supersonic-jet conditions. Data for 3-ClBzA and 2-ClBzA were recorded on a different instrument than 4-ClBzA, and it was not possible to detect singly substituted ^13^C isotopologues. Therefore, only spectroscopic constants for the ^35^Cl and ^37^Cl isotopologue were determined, as listed in Table 3. For *c*-3-ClBzA, 202 transitions were observed for the ^35^Cl species. The spectral intensity of *t*-3-ClBzA was much lower, leading to an experimental observation of 110 transitions for the main isotopologue. The high intensity of *c*-3-ClBzA aligns with the predicted dipole moment components. The spectrum of *c*-3-ClBzA is exclusively dominated by many *b*-type transitions arising from its largest dipole moment component along the *b*-axis (μ_*a*_ = −0.48 D, and μ_*b*_ = −3.65 D at B3LYP-D3(BJ)/def2-TZVPP). In contrast, for *t*-3-ClBzA, both *a*-type and *b*-type transitions (μ_*a*_ = −1.86 D, μ_*b*_ = 0.90 D) were observed experimentally. The abundance ratio of *cis* to *trans* isomers is approximately 1:1 based on the intensity of experimentally observed broadband *b*-type transitions scaled by the respective dipole moment components. The ratio is in accordance with the theory; both B3LYP and MP2 predict the isomers to be isoenergetic.

**Table 3:** Experimental Spectroscopic Fitting Parameters of *t*-3-ClBzA, *c*-3-ClBzA, and the Corresponding ^37^Cl Isotopologues Compared to VPT2 Calculations at the B3LYP-D3(BJ)/def2-TZVPP Level of Theory^a^

	*t* -3-^35^ClBzA		
	B3LYP/def2-TZVPP	*t*-3-^35^ClBzA (main, exp)	*t*-3-^37^ClBzA (exp)
*A*/MHz	3185.3	3172.4269(5)	3154.077(2)
*B*/MHz	811.61	813.00413(5)	794.13186(4)
*C*/MHz	646.81	647.22959(3)	634.47167(3)
χ*_aa_*/MHz	–55.29	–52.365(2)	–41.807(7)
χ_*bb*–*cc*_/MHz	–13.85	–14.360(2)	–10.776(8)
|χ*_ab_*|/ MHz	44.66	42.34(1)	32.90(1)
*D*_J_/kHz	0.014	0.0145(1)	0.0139(1)
*D*_JK_/kHz	–0.016	–0.0160(8)	–0.021(7)
*D*_K_/kHz	1.21	1.4(1)	1.2(2)
*d*_1_/kHz	–0.0039	–0.0042(1)	–0.0038(1)
*d*_2_/kHz	–0.00048	–0.00059(8)	–0.00053(9)
no. lines		110	84
RMS/kHz		0.5	0.5

	*c*-3-^35^ClBzA		
	B3LYP/def2-TZVPP	*c*-3-^35^ClBzA (main, exp)	*c*-3-^37^ClBzA (exp)
*A*/MHz	2349.2	2345.79472(4)	2327.41857(4)
*B*/MHz	958.27	959.44474(3)	937.52053(3)
*C*/MHz	680.63	680.97012(1)	668.34289(1)
χ*_aa_*/MHz	–39.48	–37.3833(7)	–30.633(2)
χ_*bb*–*cc*_/MHz	–29.15	–28.890(2)	–21.601(2)
|χ*_ab_*|/ MHz	54.42	51.50(2)	40.10(3)
D_J_/kHz	0.039	0.0413(1)	0.0396(1)
D_JK_/kHz	–0.15	–0.1687(8)	–0.1604(6)
D_K_/kHz	0.60	0.638(1)	0.625(2)
*d*_1_/kHz	–0.015	–0.01623(7)	–0.0153(1)
*d*_2_/kHz	–0.0013	–0.00142(3)	–0.00129(5)
no. lines		202	188
RMS/kHz		0.7	0.6

a The isomers are predicted to be isoenergetic by theory.

The relaxed scan of the interconversion between *cis* and *trans* isomer can be found in the Supporting Figure S2.^18^ The interconversion barrier purely derived from the relaxed scan is predicted to be 36.0 kJ·mol^–1^ by B3LYP-D3(BJ)/def2-TZVPP, whereas for MP2/6-311++G(d,p) the predicted barrier is around 30.1 and 31.9 kJ·mol^–1^ for MP2/6-311++G(2d,2p). The typical threshold for conformational interconversion in a supersonic-jet expansion is 5–10 kJ·mol^–1^, dependent on the carrier gas.^88^ The predicted barrier exceeds this threshold significantly; therefore, no interconversion is expected. Additionally, the conformers are almost isoenergetic, and hence, a thermodynamic driving force is limited.

### 2-Chlorobenzaldehyde

The barrier for relaxation from the *c*-2-ClBzA to *t*-2-ClBzA isomer at B3LYP-D3/def2-TZVPP is 20.9 kJ·mol^–1^, while MP2/6-311++G(d,p) and MP2/6-311++G(2d,2p) predict a barrier height of 14.2 and 16.2 kJ·mol^–1^, respectively. Similarly to the 3-ClBzA isomer, the interconversion barrier is not expected to be overcome under jet-cooled conditions. Therefore, the conformers are frozen out in the supersonic jet and under the nonequilibrium conditions of the jet, the conformational temperature is expected to be higher than the typical rotational temperature of around 1 K. The electronic energy difference is 10.5 kJ·mol^–1^ between the two isomers at the MP2/6-311++G(2d,2p) level, as shown in the torsional angle scan in Supporting Figure S2. The energy difference leads to a relative population of 2.8% for the energetically higher *c*-2-ClBzA at 85 °C, which was the temperature the compound was heated to during the experiment. As we did not observe additional lines, the molecular signal of *c*-2-ClBzA was deemed too weak to be observed on either the broadband instrument in Hartsville or in the cavity experiment.

The stabilization of *t*-2-ClBzA compared to *c*-2-ClBzA can be explained purely through electrostatic repulsion between the substituents, as this effect is already reported in the literature for 2-fluorobenzaldehyde.^12^ The relative energy differences for MP2/6-311++G(d,p) are smaller than those for B3LYP or when using a larger basis set in conjunction with MP2. This suggests a failure of MP2 in aromatic systems using small Pople basis sets, as is already documented in the literature.^76^ The addition of diffuse functions to the basis set reduces the susceptibility to this error.^76^ Spectroscopic constants were collected for the ^35^Cl *t*-2-ClBzA isotopologue and the ^37^Cl isotopologue, as listed in Table 4. Additional data for ^13^C isotopologues of *t*-2-ClBzA were collected on a cavity instrument, but due to the overlap of the strongest transitions from ^13^C isotopologues and lack of intensity for other transitions, the full set of singly substituted carbon isotopologues was not recorded.

**Table 4:** Experimental Spectroscopic Fitting Parameters of *t*-2-ClBzA and the Corresponding ^37^Cl Isotopologue Compared to VPT2 Results at the B3LYP-D3(BJ)/def2-TZVPP Level of Theory^a^

	*t* -2-^35^ClBzA		*t*-2-^37^ClBzA	
	B3LYP-D3/def2-TZVPP	*t* -2-^35^ClBzA (main, exp^a^)	B3LYP-D3/def2-TZVPP	*t*-2-^37^ClBzA (exp)
*A*/MHz	1571.61	1575.61775(8)	1574.8	1569.38135(4)
*B*/MHz	1541.5	1544.29206(7)	1499.1	1504.64924(3)
*C*/MHz	778.2	780.06375(5)	760.22	768.32345(2)
χ*_aa_*/MHz	–67.79	–59.980(1)	–53.78	–56.588(1)
χ_*bb*–*cc*_/MHz	6.357	–5.526(4)	5.36	4.960(2)
|χ*_ab_*|/ MHz	12.07	34.30(2)	7.82	1.8(6)
*D*_J_/kHz	0.0042	0.016(1)	0.0093	[0]
*D*_JK_/kHz	0.38	0.249(5)	0.36	0.423(2)
*D*_K_/kHz	–0.29	–0.111(4)	–0.27	–0.320(3)
*d*_1_/kHz	–0.019	– 0.0194(5)	–0.021	–0.0182(2)
*d*_2_/kHz	–0.022	–0.0161(2)	0.021	–0.0232(2)
no. lines		200		170
RMS/kHz		0.9		1.1

a The experimental values we show in this table are fitted with an *S*-reduced Hamiltonian in the I^*r*^ representation for consistency among the isomers, even though the *S*-reduced Hamiltonian in the III^*l*^ representation available in SPFIT/SPCAT is a closer description of the physical picture for a near-oblate top such as *t*-2-ClBzA. However, the impact of the chosen representation on the spectroscopic parameters is minimal, within 2σ of the values shown here, and has no influence on the conclusions we draw in this work. The fit for both representations can be found in the SI.

The experimental inertial defect of *t*-2-ClBzA is Δ = −0.13701(5) μÅ^2^, indicating a planar structure. The rotational constants based on equilibrium B3LYP-D3/def2-TZVPP calculations are accurately predicted with an error of less than <1%. However, the prediction of the NQCC tensor in the PAS shows a large error, as seen in Table 4. Hence, the NQCCs are utilized as a point of comparison between experiment and theory, which is the subject of discussion in the next section.

### Benchmarking Nuclear Quadrupole Coupling Constants

**Figure 3:**
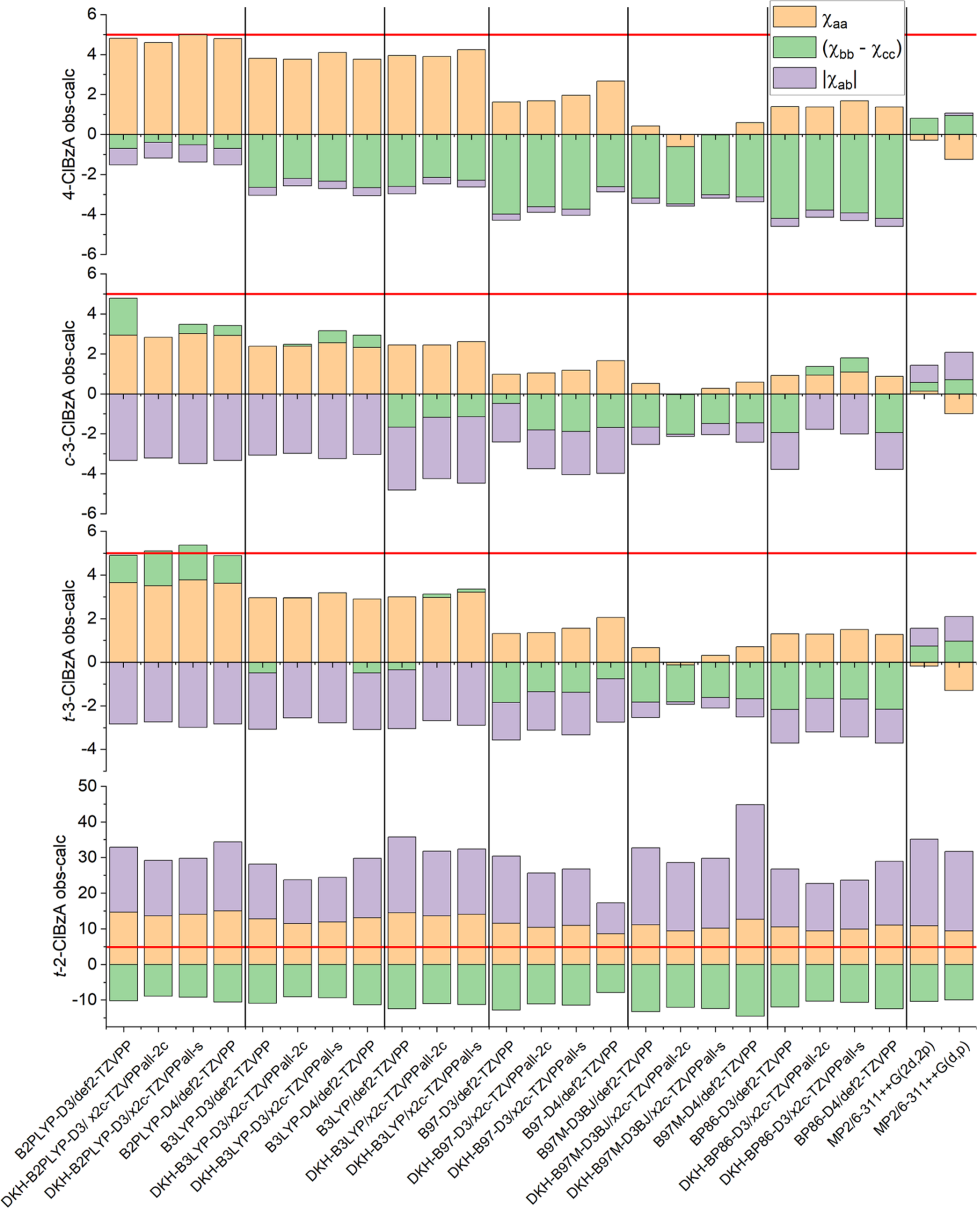
Calculated nuclear quadrupole coupling components in PAS subtracted from experimental values. All values are in MHz. The static red line at Δ*χ* = 5 MHz, chosen for ease of comparison, demonstrates the increase in error along the series with *t*-2-ClBzA being significantly higher than the rest. Experimental uncertainties are <1 MHz for all parameters.

The NQCCs of all four isomers were predicted with 25 computational methods and compared to experimental spectroscopic parameters for the purpose of benchmarking theory, as NQC provides insight into the electronic structure, as shown in Figure 3. Each ClBzA isomer is spectroscopically confirmed to be near-planar within the ab-plane. Hence, the NQCC tensor consists of three independent elements that were fitted: χ*_aa_*, χ_*bb*–*cc*_, and χ*_ab_*. These three fitting parameters fully describe the tensor since χ_*ac*_ and χ_*bc*_ are near-zero due to symmetry. The absolute χ*_ab_* values from each calculation were compared with the experimental results because the sign of χ*_ab_* is not experimentally determinable.

In the case of 4-ClBzA, for B2PLYP and B3LYP calculations, the determined value of χ*_aa_* has an average deviation of 4.2 MHz from the experimental results. The best computational result is provided by MP2/6-311++G(2d,2p), where all deviations for the predictions of the NQCCs are <1 MHz. The addition of diffuse functions to the basis set is important for an accurate description of the EFG, as its description of χ*_aa_* for 4-ClBzA is improved by 0.96 MHz, an effect that is also observed in the nuclear axis system.

The prediction quality for the two 3-ClBzA conformers is equal, with absolute deviations between calculation and experimental values for χ*_aa_* ranging from 0.1 MHz to 3.8. Notably, the error for the off-diagonal element χ_*ab*_ increases compared to 4-ClBzA from a mean deviation of 0.4–2.1 MHz between all methods. For a more direct comparison between the structures, the relative deviation for χ*_ab_* can be determined, which shows comparable relative deviations of 4%, averaged over all methods for both 4-ClBzA and 3-ClBzA. The best-performing DFT method in describing the NQCC is B97M-D3BJ/x2c-TZVPP-all, although the differences in calculation quality for different basis sets are small. A relativistic treatment of the chlorine and a nonrelativistic basis set with the same methods demonstrate that spin–orbit effects are negligible for a theoretical treatment of chlorine’s NQC, which is in accordance with previous work.^67^

For 2-ClBzA, all functionals fail to accurately predict the NQCCs. The mean deviations for χ_aa_ and |χ_ab_| are 11.8 and 17.7 MHz, respectively, among all methods tested in the PAS. When comparing to a relative deviation for |χ_*ab*,rel_|, the percentile error increases by 1 order of magnitude to 51%. For χ_*bb*–*cc*_, a sign flip is observed between experiment and theory for the ^35^Cl main isotopologue. This is surprising because the rotational Hamiltonian fit is converged to the experimental accuracy limit with 200 contributing transitions. This large error in NQCC prediction poses a problem in the initial phase of the assignment process, as it can lead to a different ordering of the predicted hyperfine transitions compared to the experimentally observed ordering, as shown in Figure 5. However, this large discrepancy also helps to confirm the assignment, as χ*_ab_* is crucial for the convergence of the fit. Upon substitution of ^35^Cl with its heavier isotope ^37^Cl, a similarly large change of NQCC and even a flip in the sign of a tensor component as displayed by 2-ClBzA has been previously observed for near-oblate rotors.^89^

### Projection of NQCC for 2-ClBzA

**Figure 4:**
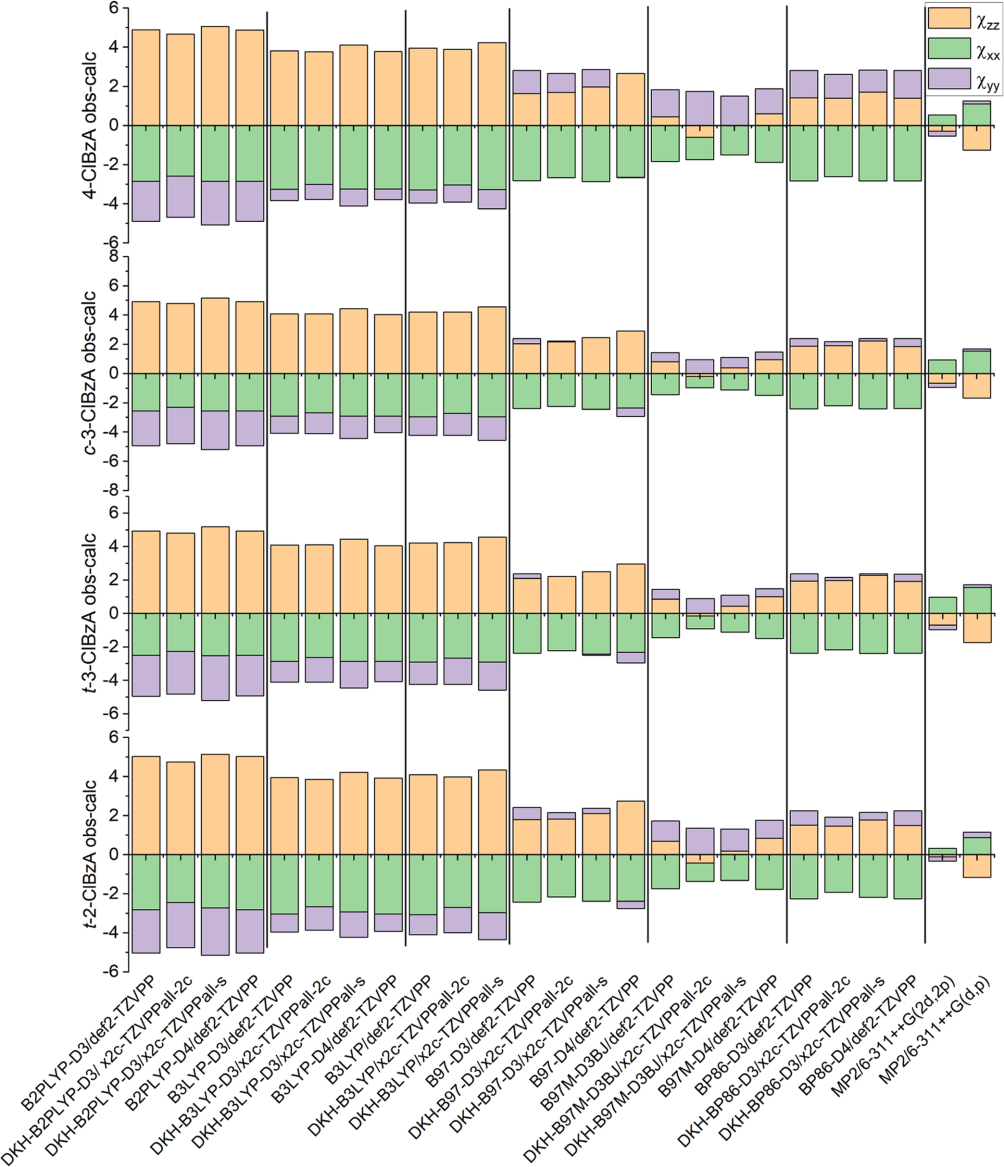
Calculated nuclear quadrupole coupling components in the nuclear axis system are subtracted from experimental values. All values are in MHz.

When the NQCC coupling constants in the nuclear axis system are compared, as displayed in Figure 4, it is apparent that the description of the electronic environment is of equal quality for all four isomers. In the nuclear axis system, the *z*-axis is defined along the steepest gradient of the EFG tensor, which is approximately along the chlorine carbon σ bond. Most methods perform similarly, with deviations in the description of χ*_zz_* between −1.3 and 5.1 to 5.1 MHz among all conformers and methods. Hence, the EFG is similar, as expected for isoelectronic systems, and as is the theoretical description thereof. Instead, the poor performance of the NQCC predictions for 2-ClBzA is caused by the projection of the NQCC from the local axis system into the PAS of the moments of inertia.

Computational predictions put the *a*-axis of 2-ClBzA almost parallel to the *z*-axis of the nuclear axis system. The angle Θ*_az_* between the *a* principal axis and the *z*-axis of the nuclear quadrupole coupling tensor, which can be calculated for planar molecules from the NQCC, ranges between 1.2° for B97M-D4/def2-TZVPP to 13.1° for B97-D4/def2-TZVPP. In contrast, the experimental angle is Θ*_az_* = 19.1°. A very small variation in the angle between these two vectors may already affect the predictions for χ*_aa_* and χ*_ab_* significantly. The cause of the error in the axis system transformation is likely rooted in insufficient theoretical treatment, as the error in χ*_ab_* directly correlates with the transformation angle Θ*_az_* between those two axis systems, as demonstrated in Figure 6(a). However, there exists no direct correlation between Θ*_az_* from theory and a single structural parameter, such as the distance between the formyl carbon and the chlorine atom, suggesting that several parameters might influence it, as illustrated in Figure 6b.

**Figure 5:**
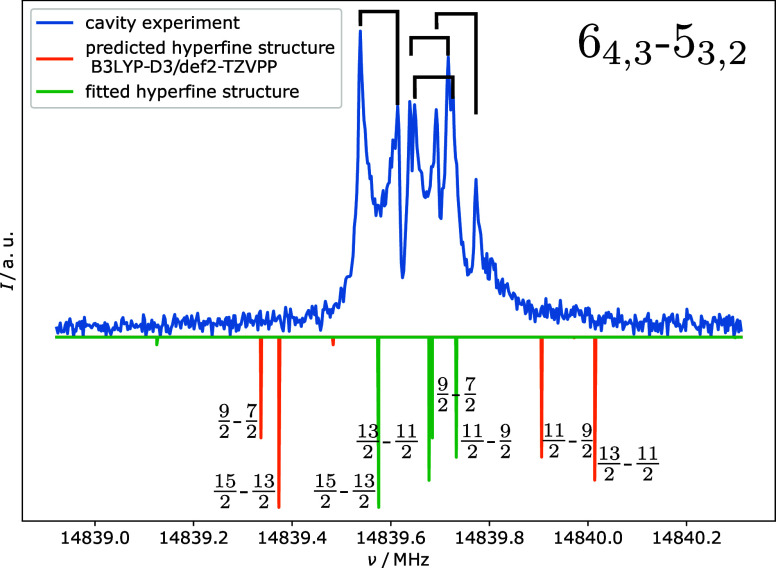
Rotational hyperfine splitting of chlorine as observed in 2-^35^ClBzA with the QCUMBER spectrometer. Above in blue is the experimental spectrum; each peak appears as a Doppler doublet. Orange shows the hyperfine splitting as predicted (B3LYP-D4/def2-TZVPP), utilizing the χ*_gg_* values from theory and combining them with the experimental rotational constants and centrifugal distortion parameters in the Hamiltonian. The green plot shows a line spectrum using all of the fitted experimental parameters, including an experimental fit of χ*_aa_*, χ_*bb*–*cc*_, and χ*_ac_*. The rotational quantum numbers *J*_K_a_,K_c__ are given in the top right, while each hyperfine transition is labeled with its assigned *F* = *J* + *I* quantum numbers. The nuclear spin *I* of chlorine is *I* = 3/2.

**Figure 6:**
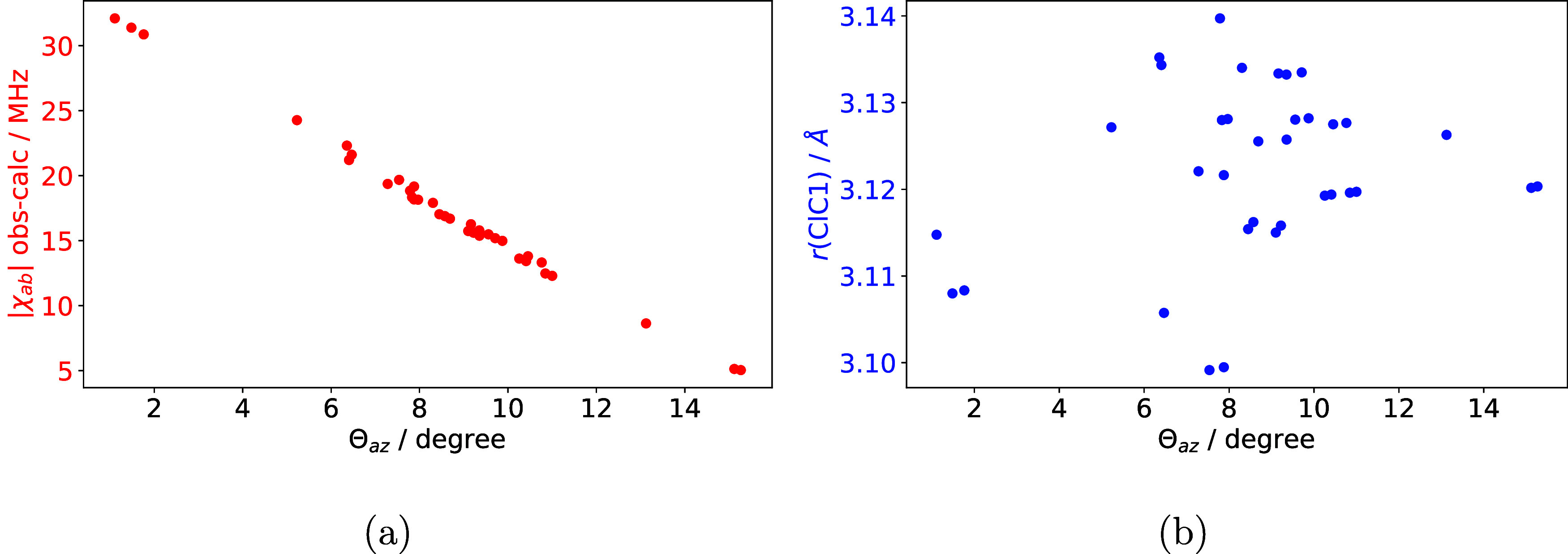
(a) Correlation of Θ*_az_* with the error in |χ*_ab_*| and (b) the distance between the Cl and C1 atoms in each equilibrium geometry of 2-t-ClBzA as an indicator for the substituent distance.

As an interaction between the two substituents on the benzene ring is the most chemically intuitive explanation for a distortion in geometry, we used Kraitchman’s equations to determine the absolute coordinates of chlorine through substitution with ^37^Cl. For 2-ClBzA, coordinates in the PAS from theory are substantially different from the coordinates acquired through substitution, with an average deviation between *r*_e_ and *r*_s_ of 0.519(4) Å among all methods. This difference is beyond an expected error for the substitution coordinates of 0.02 Å.^1^ Analogous analysis of the *r*_s_ and *r*_e_ coordinates for 3-ClBzA shows a difference of 0.014(6) and 0.009(7) Å for chlorine coordinates of the *trans* and *cis* conformers, respectively.

**Figure 7:**
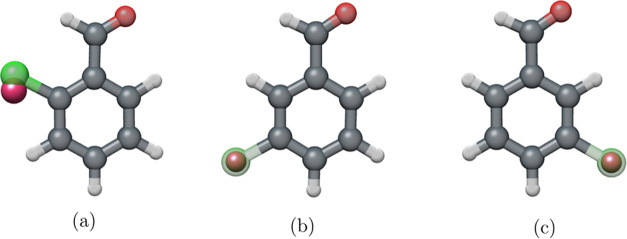
Overlay of MP2/6-311++G(2d,2p) optimized geometry of (a) *t*-2-ClBzA, (b) *t*-3-ClBzA, and (c) *c*-3-ClBzA on Kraitchman substitution coordinates of chlorine in pink.

An overlay of the *r*_s_ coordinate for chlorine with an equilibrium structure prediction at the MP2/6-311++G(2d,2p) level in Figure 7 shows that the atomic coordinates of the chlorine atom do not match for *t*-2-ClBzA. As mentioned previously, this error far exceeds acceptable methodical errors for Kraitchman’s substitution coordinates. In contrast to the other near-prolate isomers, 2-ClBzA is a near-oblate rotor with an asymmetry parameter κ(^35^Cl) = 0.9212481(3). Upon chlorine substitution, the asymmetry decreases to κ(^37^Cl) = 0.8383835(1), which indicates a comparatively large rotation of the PAS in the *ab*-plane. Kraitchman’s method is known to be unreliable for near-oblate tops with a large axis rotation due to the noncancellation of vibrational contributions upon substitution.^86,90,91^ In fact, Kraitchman’s method is only strictly valid for the equilibrium moments of inertia *I*_e_^*g*^, which are approximated by the experimental ground-state moments of inertia *I*_0_^*g*^ = *I*_e_^*g*^ + ϵ_*g*_. If the rovibrational contributions ϵ_*g*_ strongly differ between the most abundant and singly substituted isotopologue, then the contributions are non-negligible. When the inertial PAS experiences a large rotation ϕ_0_ upon substitution, ϵ_*g*_ may change drastically upon substitution and cause a failure of Kraitchman’s method.^90^

The error contributed by noncanceling vibrational effects can be estimated through the difference of *δP*_e_^*gg*^ – *δP*_0_^*gg*^, where *δP*_*gg*_ = *P*_iso_^*gg*^ – *P*_main_^*gg*^. The planar moment *P*_*gg*_ is defined as *P*_*gg*_ = ∑_*i*_*m*_*i*_*g*_*i*_^2^, where *m*_*i*_ is the mass of each atom *i* and *g*_*i*_ its respective principal coordinate, with *g* = *a*, *b*, *c*.^92^ The equilibrium and ground-state planar moments were calculated for each main ^35^Cl isomer and respective ^37^Cl isotopologue with B3LYP-D3/aug-cc-pVTZ for comparison.^93−99^ In the case of 4-ClBzA, *t*-3-ClBzA and *c*-3-ClBzA, |*δP*_e_^*gg*^ – *δP*_0_^*gg*^| for *g* = *a*, *b* are equal to 0.07 μÅ^2^ or smaller, as shown in Table 5. In contrast to that, |*δP*_e_^*gg*^ – *δP*_0_^*gg*^| is larger by almost an order of magnitude for *t*-2-ClBzA, indicating that the vibrational contributions are noncanceling. Thus, the inherent approximation for *r*_s_ structure determination is invalid for 2-ClBzA.

**Table 5:** Difference in Planar Moment Components *δP*_*gg*_ = *P*_iso_^*gg*^ – *P*_main_^*gg*^ for the Respective Chlorine Isotopologue Calculated by B3LYP-D3(BJ)/aug-cc-pVTZ for *t*-2-ClBzA and 4-ClBzA^a^

	2-ClBzA (*δP*_e_^*gg*^)	(*δP*_0_^*gg*^)	4-ClBzA (*δP*_e_^*gg*^)	(*δP*_0_^*gg*^)
*δP*_*aa*_	9.4836	9.2824	17.8949	17.9693
*δP*_*bb*_	0.5166	0.7566	0.0442	0.0446
*δP*_*cc*_	0.00011	–0.0012	0.00005	0.00014
*δP*_e_^*aa*^ – *δP*_0_^*aa*^	0.2012		–0.0744	
*δP*_e_^*bb*^ – *δP*_0_^*bb*^	–0.2405		–0.0004	
*δP*_e_^*cc*^ – *δP*_0_^*cc*^	0.0013		–0.00009	

a The calculations for 3-ClBzA were also conducted, but the rotational effects cancel out upon substitution, similar to 4-ClBzA. All values are in μÅ^2^.

Gas-phase Raman experiments were conducted at the *curry* setup,^20−23^ measuring the low-energy frequencies up to 700 cm^–1^ of 2-ClBzA and 4-ClBzA. While the full spectrum has not been assigned, the 2-ClBzA spectrum shows a band feature at 186 cm^–1^, which is unexplained in the harmonic approximation (see Supporting Figure S4). It is possibly a resonance of the formyl torsional vibration. There is no corresponding band feature in the 4-ClBzA spectrum. While the vibrational spectrum of 2-ClBzA is not fully understood, it also serves as an indication that vibrational contributions may be non-negligible in this system, in contrast to the other isomers, due to anharmonic effects.

The analysis in Table 5 and the vibrational data indicate that the 2-ClBzA Kraitchman’s coordinate for chlorine is unreliable. Seeing that the low-energy vibrations have a substantial impact on the structure analysis, it appears likely that vibrational contributions are also responsible for the high uncertainty in the principal axis system calculations and, hence, the NQCC predictions. The NQCC of 2-ClBzA is very sensitive to the angle between the C–Cl bond and the principal axis system, which is influenced by the vibrations in the system. It is particularly interesting whether this error is present when moving to heavier homologous of chlorine, as errors in NQCC predictions, as seen in Figure 5, are even more relevant for heavy nuclei with a large quadrupole moment and large nuclear spin.

## Conclusions

The spectroscopic rotational parameters of 4-ClBzA, 3-ClBzA, and 2-ClBzA were reported by fitting the parameters of the rotational Hamiltonian, confirming the existence of four planar isomers in a jet-cooled expansion. For 4-ClBzA and 2-ClBzA, a single isomer each has been observed, while both the *cis* and *trans* conformers of 3-ClBzA were detected. The two conformers are isoenergetic and observed in a 1:1 ratio in the jet based on the experimental intensities of their transitions. Only the *t*-2-ClBzA isomer, which is 10.5 kJ·mol^–1^ lower in energy than *c*-2-ClBzA, was experimentally observed.

Complete monosubstituted isotopic data of ^37^Cl and ^13^C was collected for 4-ClBzA, which allowed for extensive structure fitting of a substitution geometry *r*_s_, a ground-state geometry *r*_0_, a mass reduced geometry *r*_*m*_^(1)^, and a semiexperimental equilibrium structure *r*_e_^se^.

The complete NQCC tensor of each conformer was experimentally determined for the main ^35^Cl isotopologue and their respective ^37^Cl isotopologue. The NQCC terms were extensively benchmarked with 25 quantum chemical methods, which all described the NQCC in the nuclear axis system with an error <6 MHz. The best predictions for the chlorobenzaldehydes were given by MP2/6-311++G(2d,2p), which gave equilibrium values for both rotational constants and NQCC with low errors when compared to the experiment. The only exception is the 2-ClBzA isomer, in which case the NQCCs projected into the PAS, especially the off-diagonal element χ_*ab*_, lose quality. An analysis of the Kraitchman’s coordinates of chlorine suggests that vibrational effects are non-negligible in the analysis of 2-ClBzA. The fact that the 2-ClBzA is particularly error prone is a combination of the near-oblate nature of the molecule, which makes the PAS highly uncertain in the *ab*-plane, and the fact that the *a*-axis is near parallel to the Cl–C bond. While the relative error caused by rotational constant predictions is minor, the projection of NQCC into the PAS is very sensitive. Therefore, this series of chlorobenzaldehydes provides an excellent experimental double benchmark for the computational projection of NQCC into the PAS.

A rigorous treatment of NQCC will be increasingly significant for nuclei like bromine and iodine, which have an increase in size, nuclear quadrupole moment, and NQCC. Projections become more sensitive with the pursuit of chiral or floppy, weakly bound species. Hence, the NQCC needs to be treated with great scrutiny if failure is already observed in these comparatively rigid systems, with their similar rigidity demonstrated by their small centrifugal distortion constants. Incorrect projections of electronic parameters also extend beyond NQCC to other parameters, such as electric dipole moments, which are harder to access experimentally. Rigorous benchmarking of projection-dependent spectroscopic parameters is vital to ensuring the reliability of these predictions.

## Supporting Information Available

All calculation and fit files are published on our data server in Göttingen, Gro.data (https://doi.org/10.25625/SCSHJK).^18^ In addition, the raw spectroscopic data from the Cavity instrument can be found there. The boradand spectra contains still unpublished complexes so we will wait to publish this data (PDF)

## References

[ref1] Gordy W.; Cook R. L. Microwave Molecular Spectra; Wiley, 1984.

[ref2] Watson J. K. G.; Roytburg A.; Ulrich W. Least-Squares Mass-Dependence Molecular Structures. J. Mol. Spectrosc. 1999, 196, 102–119. 10.1006/jmsp.1999.7843. 10361061

[ref3] Puzzarini C.; Stanton J. F.; Gauss J.; Gauss J. J. Quantum-chemical calculation of spectroscopic parameters for rotational spectroscopy. Int. Rev. Phys. Chem. 2010, 29, 273–367. 10.1080/01442351003643401.

[ref4] Bailey W. C. Calculation of 14-N and 33-S Quadrupole Coupling Constants on Optimized Molecular Structures of Thiazole. J. Mol. Spectrosc. 2001, 209, 57–59. 10.1006/jmsp.2001.8408.

[ref5] Bailey W. C. HF-DFT calculation of 14-N and 35-Cl quadrupole coupling constants on optimized molecular structures of pyridine and the monochloropyridines. J. Mol. Struct.:THEOCHEM 2001, 541, 195–201. 10.1016/S0166-1280(00)00765-X.

[ref6] Leung H. O.; Cashion W. T.; Duncan K. K.; Hagan C. L.; Joo S. Nuclear quadrupole hyperfine structure in the microwave spectrum of HCl-N_2_O: Electric field gradient perturbation of N_2_O by HCl. J. Chem. Phys. 2004, 121, 237–247. 10.1063/1.1756871. 15260541

[ref7] Bizzocchi L.; Alessandrini S.; Melosso M.; Puzzarini C. Dipolar spin-spin coupling as an auxiliary tool for the structure determination of small isolated molecules. Phys. Chem. Chem. Phys. 2022, 24, 15173–15181. 10.1039/D2CP01124G. 35703976

[ref8] Leung H. O.; Marshall M. D.; Hong S. Microwave Spectrum and Novel Molecular Structure of (Z)-1-Chloro-3,3,3-trifluoropropene-Acetylene. J. Phys. Chem. A 2023, 127, 6241–6250. 10.1021/acs.jpca.3c03517. 37471081

[ref9] Signore J.; Novick S.; Cooke S.; Pringle W.; Nguyen H. V. L. In Combination of Iodine Quadrupole Coupling and Essentially Free Methyl Internal Rotation in 3-Iodotoluene, Proceedings of the 2021 International Symposium on Molecular Spectroscopy; 2021.

[ref10] Dohmen R.; Fedosov D.; Obenchain D. A. Benchmarking the quadrupolar coupling tensor for chlorine to probe weak-bonding interactions. Phys. Chem. Chem. Phys. 2023, 25, 2420–2429. 10.1039/D2CP04067K. 36598167

[ref11] Møllendal H.; Gundersen S.; Tafipolsky M. A.; Volden H. V. The molecular structure of benzene derivatives, part 2:4-chloro-benzaldehyde by joint analysis of gas electron diffraction, microwave spectroscopy and ab initio molecular orbital calculations. J. Mol. Struct. 1998, 444, 47–56. 10.1016/S0022-2860(97)00310-4.

[ref12] Sun W.; Lozada I. B.; Wijngaarden J. V. Fourier Transform Microwave Spectroscopic and ab Initio Study of the Rotamers of 2-Fluorobenzaldehyde and 3-Fluorobenzaldehyde. J. Phys. Chem. A 2018, 122, 2060–2068. 10.1021/acs.jpca.7b11673. 29406715

[ref13] González S. R.; Villamafian R. M.; Alonso J. L. Microwave Spectrum of 4-Fluorobenzaldehyde. J. Mol. Struct. 1988, 190, 79–84. 10.1016/0022-2860(88)80272-2.

[ref14] Smith M.; Short B. D.; Ruthven A. M.; Thomas K. M.; Hang M. J.; Brown G. G. Chirped-pulse microwave spectrum and ab initio calculations of four distinct conformers of 3-vinylbenzaldehyde. J. Mol. Spectrosc. 2015, 307, 49–53. 10.1016/j.jms.2014.12.014.

[ref15] Schmitz D.; Shubert V. A.; Betz T.; Schnell M. Multi-resonance effects within a single chirp in broadband rotational spectroscopy: The rapid adiabatic passage regime for benzonitrile. J. Mol. Spectrosc. 2012, 280, 77–84. 10.1016/j.jms.2012.08.001.

[ref16] Balle T. J.; Flygare W. H. Fabry-Perot cavity pulsed Fourier transform microwave spectrometer with a pulsed nozzle particle source. Rev. Sci. Instrum. 1981, 52, 33–45. 10.1063/1.1136443.

[ref17] Andresen U.; Dreizler H.; Kretschmer U.; Stahl W.; Thomsen C. A molecular beam Fourier transform microwave spectrometer for analytical purposes. Anal. Chem. 1994, 349, 272–276. 10.1007/BF00323202.

[ref18] Dohmen R.; Arnold S.; Garrett J.; Kempken B.; Hartwig B.; Schröder B.; Pinacho P.; Schnell M.; Brown G. G.; Obenchain D. A. Supporting information for “Varying Projection Quality of Good Local Electric Field Gradients of Monochlorobenzaldehydes”. 2024. 10.25625/SCSHJK. PMC11789141 39823215

[ref19] Schnell M.; Banser D.; Grabow J.-U. Coaxially aligned electrodes for Stark-effect applied in resonators using a supersonic jet Fourier transform microwave spectrometer. Rev. Sci. Instrum. 2004, 75, 2111–2115. 10.1063/1.1755439.

[ref20] Lüttschwager N. O. B.; Suhm M. A. Stretching and folding of 2-nanometer hydrocarbon rods. Soft Matter 2014, 10, 4885–4901. 10.1039/C4SM00508B. 24866111

[ref21] Forsting T.; Gottschalk H. C.; Hartwig B.; Mons M.; Suhm M. A. Correcting the record: the dimers and trimers of trans-N-methylacetamide. Phys. Chem. Chem. Phys. 2017, 19, 10727–10737. 10.1039/C6CP07989J. 28045153

[ref22] Gawrilow M.; Suhm M. A. 2-Methoxyethanol: harmonic tricks, anharmonic challenges and chirality-sensitive chain aggregation. Phys. Chem. Chem. Phys. 2020, 22, 15303–15311. 10.1039/D0CP02488K. 32626860

[ref23] Hartwig B. Diols under Investigation: Benchmarking their Monomers, Dimers and Chirality Recognition, 2022.

[ref24] Pickett H. M. The fitting and prediction of vibration-rotation spectra with spin interactions. J. Mol. Spectrosc. 1991, 148, 371–377. 10.1016/0022-2852(91)90393-O.

[ref25] Kisiel Z. PROSPE—Programs for ROtational SPEctroscopy; 2001. http://info.ifpan.edu.pl/ kisiel/prospe.htm.

[ref26] Kisiel Z. Least-squares mass-dependence molecular structures for selected weakly bound intermolecular clusters. J. Mol. Spectrosc. 2003, 218, 58–67. 10.1016/S0022-2852(02)00036-X.

[ref27] Budden A. S.; Grozin A. G.; Rohde A.; Jeffries B.; Pugmire C.; Brendes C.; Parfitt D.; Edmonson E.; Stuyf J.; Abbott L. gle. 2022. https://glx.sourceforge.io/.

[ref28] OriginPro, Version; OriginLab Corporation: Northampton, MA.

[ref29] Python Language Reference, 2000. https://www.python.org/.

[ref30] Rossum G. V.; Drake F. L. Python 3 Reference Manual; CreateSpace, 2009.

[ref31] Hunter J. D. Matplotlib: A 2D graphics environment. Comput. Sci. Eng. 2007, 9, 90–95. 10.1109/MCSE.2007.55.

[ref32] pandas development team, T. pandas-dev/pandas, Pandas 2020. https://pandas.pydata.org/.

[ref33] Harris C. R.; Millman K. J.; van der Walt S. J.; Gommers R.; Virtanen P.; Cournapeau D.; Wieser E.; Taylor J.; Berg S.; Smith N. J.; et al. Array programming with NumPy. Nature 2020, 585, 357–362. 10.1038/s41586-020-2649-2. 32939066 PMC7759461

[ref34] Pettersen E. F.; Goddard T. D.; Huang C. C.; Couch G. S.; Greenblatt D. M.; Meng E. C.; Ferrin T. E. UCSF ChimeraA visualization system for exploratory research and analysis. J. Comput. Chem. 2004, 25, 1605–1612. 10.1002/jcc.20084. 15264254

[ref35] Neese F. Software update: The ORCA program system—Version 5.0. Wiley Interdiscip. Rev.:Comput. Mol. Sci. 2022, 12, e1606 10.1002/wcms.1606.

[ref36] Valeev E. F. Libint: A library for the evaluation of molecular integrals of many-body operators over Gaussian functions, 2017. http://libint.valeyev.net/, version 2.7.1.

[ref37] Lehtola S.; Steigemann C.; Oliveira M. J.; Marques M. A. Recent developments in LIBXCA comprehensive library of functionals for density functional theory. SoftwareX 2018, 7, 1–5. 10.1016/j.softx.2017.11.002.

[ref38] Becke A. D. Density-functional exchange-energy approximation with correct asymptotic behavior. Phys. Rev. A 1988, 38, 3098–3100. 10.1103/PhysRevA.38.3098. 9900728

[ref39] Lee C.; Yang W.; Parr R. G. Development of the Colic-Salvetti correlation-energy formula into a functional of the electron density. Phys. Rev. B 1988, 37, 785–789. 10.1103/physrevb.37.785. 9944570

[ref40] Becke A. D. Density-functional thermochemistry. III. The role of exact exchange. J. Chem. Phys. 1993, 98, 5648–5652. 10.1063/1.464913.

[ref41] Stephens P. J.; Devlin F. J.; Chabalowski C. F.; Frisch M. J. Ab Initio calculation of vibrational absorption and circular dichroism spectra using density functional force fields. J. Phys. Chem. A 1994, 98, 11623–11627. 10.1021/j100096a001.

[ref42] Zhang I. Y.; Wu J.; Xu X. Extending the reliability and applicability of B3LYP. Chem. Commun. 2010, 46, 3057–3070. 10.1039/c000677g. 20372746

[ref43] Grimme S.; Antony J.; Ehrlich S.; Krieg H. A consistent and accurate ab initio parametrization of density functional dispersion correction (DFT-D) for the 94 elements H-Pu. J. Chem. Phys. 2010, 132, 154104–154124. 10.1063/1.3382344. 20423165

[ref44] Grimme S.; Ehrlich S.; Goerigk L. Effect of the damping function in dispersion corrected density functional theory. J. Comput. Chem. 2011, 32, 1456–1465. 10.1002/jcc.21759. 21370243

[ref45] Caldeweyher E.; Bannwarth C.; Grimme S. Extension of the D3 dispersion coefficient model. J. Chem. Phys. 2017, 147, 034112–034119. 10.1063/1.4993215. 28734285

[ref46] Bursch M.; Caldeweyher E.; Hansen A.; Neugebauer H.; Ehlert S.; Grimme S. Understanding and Quantifying London Dispersion Effects in Organometallic Complexes. Acc. Chem. Res. 2019, 52, 258–266. 10.1021/acs.accounts.8b00505. 30586286

[ref47] Caldeweyher E.; Ehlert S.; Hansen A.; Neugebauer H.; Spicher S.; Bannwarth C.; Grimme S. A generally applicable atomic-charge dependent London dispersion correction. J. Chem. Phys. 2019, 150, 154122 10.1063/1.5090222. 31005066

[ref48] Najibi A.; Goerigk L. DFT-D4 counterparts of leading meta-generalized-gradient approximation and hybrid density functionals for energetics and geometries. J. Comput. Chem. 2020, 41, 2562–2572. 10.1002/jcc.26411. 32870518

[ref49] Grimme S. Semiempirical hybrid density functional with perturbative second-order correlation. J. Chem. Phys. 2006, 124, 034108 10.1063/1.2148954. 16438568

[ref50] Stoychev G. L.; Auer A. A.; Neese F. Automatic Generation of Auxiliary Basis Sets. J. Chem. Theory Comput. 2017, 13, 554–562. 10.1021/acs.jctc.6b01041. 28005364

[ref51] Perdew J. P. Density-functional approximation for the correlation energy of the inhomogeneous electron gas. Phys. Rev. B 1986, 33, 8822–8824. 10.1103/PhysRevB.33.8822. 9938299

[ref52] Perdew J. P. Erratum: Density-functional approximation for the correlation energy of the inhomogeneous electron gas. Phys. Rev. B 1986, 34, 7406 10.1103/PhysRevB.34.7406. 9949100

[ref53] Mardirossian N.; Head-Gordon M. Mapping the genome of meta-generalized gradient approximation density functionals: The search for B97M-V. J. Chem. Phys. 2015, 142, 74111 10.1063/1.4907719. 25702006

[ref54] Najibi A.; Goerigk L. The Nonlocal Kernel in van der Waals Density Functionals as an Additive Correction: An Extensive Analysis with Special Emphasis on the B97M-V and wB97M-V Approaches. J. Chem. Theory Comput. 2018, 14, 5725–5738. 10.1021/acs.jctc.8b00842. 30299953

[ref55] Weigend F.; Ahlrichs R. Balanced basis sets of split valence, triple zeta valence and quadruple zeta valence quality for H to Rn: Design and assessment of accuracy. Phys. Chem. Chem. Phys. 2005, 7, 3297–3305. 10.1039/b508541a. 16240044

[ref56] Rappoport D.; Furche F. Property-optimized Gaussian basis sets for molecular response calculations. J. Chem. Phys. 2010, 133, 134105 10.1063/1.3484283. 20942521

[ref57] Andrae D.; Häußermann U.; Dolg M.; Stoll H.; Preuß H. Energy-adjusted ab initio pseudopotentials for the second and third row transition elements. Theor. Chim. Acta 1990, 77, 123–141. 10.1007/BF01114537.

[ref58] Peterson K. A.; Figgen D.; Goll E.; Stoll H.; Dolg M. Systematically convergent basis sets with relativistic pseudopotentials. II. Small-core pseudopotentials and correlation consistent basis sets for the post-d group 16–18 elements. J. Chem. Phys. 2003, 119, 11113–11123. 10.1063/1.1622924.

[ref59] Pollak P.; Weigend F. Segmented Contracted Error-Consistent Basis Sets of Double- and Triple-Zeta Valence Quality for One- and Two-Component Relativistic All-Electron Calculations. J. Chem. Theory Comput. 2017, 13, 3696–3705. 10.1021/acs.jctc.7b00593. 28679044

[ref60] Franzke Y. J.; Treß R.; Pazdera T. M.; Weigend F. Error-consistent segmented contracted all-electron relativistic basis sets of double- and triple-zeta quality for NMR shielding constants. Phys. Chem. Chem. Phys. 2019, 21, 16658–16664. 10.1039/C9CP02382H. 31317138

[ref61] Douglas M.; Kroll N. M. Quantum electrodynamical corrections to the fine structure of helium. Ann. Phys. 1974, 82, 89–155. 10.1016/0003-4916(74)90333-9.

[ref62] Hess B. A. Relativistic electronic-structure calculations employing a two-component no-pair formalism with external-field projection operators. Phys. Rev. A 1986, 33, 3742–3748. 10.1103/PhysRevA.33.3742. 9897114

[ref63] Jansen G.; Heß B. A. Revision of the Douglas-Kroll transformation. Phys. Rev. A 1989, 39, 6016–6017. 10.1103/PhysRevA.39.6016. 9901188

[ref64] Cheng L.; Stopkowicz S.; Stanton J. F.; Gauss J. The route to high accuracy in ab initio calculations of Cu quadrupole-coupling constants. J. Chem. Phys. 2012, 137, 224302 10.1063/1.4767767. 23248998

[ref65] Stopkowicz S.; Cheng L.; Harding M. E.; Puzzarini C.; Gauss J. The bromine nuclear quadrupole moment revisited. Mol. Phys. 2013, 111, 1382–1389. 10.1080/00268976.2013.796072.

[ref66] Obenchain D. A.; Frank D. S.; Grubbs G. S.; Pickett H. M.; Pickett H. M.; Novick S. E. The covalent interaction between dihydrogen and gold: A rotational spectroscopic study of H_2_-AuCl. J. Chem. Phys. 2017, 146, 204302 10.1063/1.4983042. 28571327 PMC5648549

[ref67] Gaul K.; Berger R. Quasi-relativistic study of nuclear electric quadrupole coupling constants in chiral molecules containing heavy elements. Mol. Phys. 2020, 118, e1797199 10.1080/00268976.2020.1797199.

[ref68] Møller C.; Plesset M. S. Note on an Approximation Treatment for Many-Electron. Phys. Rev. 1934, 46, 618 10.1103/PhysRev.46.618.

[ref69] Krishnan R.; Binkley J. S.; Seeger R.; Pople J. A. Self-consistent molecular orbital methods. XX. A basis set for correlated wave functions. J. Chem. Phys. 1980, 72, 650–654. 10.1063/1.438955.

[ref70] McLean A. D.; Chandler G. S. Contracted Gaussian basis sets for molecular calculations. I. Second row atoms, Z = 11–18. J. Chem. Phys. 1980, 72, 5639–5648. 10.1063/1.438980.

[ref71] Blaudeau J. P.; McGrath M. P.; Curtiss L. A.; Radom L. Extension of Gaussian-2 (G2) theory to molecules containing third-row atoms K and Ca. J. Chem. Phys. 1997, 107, 5016–5021. 10.1063/1.474865.

[ref72] Curtiss L. A.; McGrath M. P.; Blaudeau J. P.; Davis N. E.; Binning R. C.; Radom L. Extension of Gaussian-2 theory to molecules containing third-row atoms Ga-Kr. J. Chem. Phys. 1995, 103, 6104–6113. 10.1063/1.470438.

[ref73] Clark T.; Chandrasekhar J.; Spitznagel G. W.; Schleyer P. V. R. Efficient diffuse function-augmented basis sets for anion calculations. III. The 3-21+G basis set for first-row elements, Li-F. J. Comput. Chem. 1983, 4, 294–301. 10.1002/jcc.540040303.

[ref74] Frisch M. J.; Pople J. A.; Binkley J. S. Self-consistent molecular orbital methods 25. Supplementary functions for Gaussian basis sets. J. Chem. Phys. 1984, 80, 3265–3269. 10.1063/1.447079.

[ref75] Marshall M. S.; Sears J. S.; Burns L. A.; Brédas J.-L.; Sherrill C. D. An Error and Efficiency Analysis of Approximations to Møller Plesset Perturbation Theory. J. Chem. Theory Comput. 2010, 6, 3681–3687. 10.1021/ct100468f.

[ref76] Burns L. A.; Murdock D.; Vaccaro P. H. An exploration of electronic structure and nuclear dynamics in tropolone. I. The XA11 ground state. J. Chem. Phys. 2006, 124, 204307 10.1063/1.2200343. 16774332

[ref77] Nielsen H. H. The Vibration-rotation Energies of Molecules and their Spectra in the Infrared; der Physik Handbuch.; Flügge S., Eds.; Springer, 1959; Vol. 37, pp 173–313.

[ref78] Amat G.; Nielsen H. H.; Tarrago G. Rotation-Vibration of Polyatomic Molecules; Dekker 1971, 10.1119/1.1986798.

[ref79] Clabo D.; Allen W. D.; Remington R. B.; Yamaguchi Y.; Schaefer H. F. A systematic study of molecular vibrational anharmonicity and vibrationrotation interaction by self-consistent-field higher-derivative methods. Asymmetric top molecules. Chem. Phys. 1988, 123, 187–239. 10.1016/0301-0104(88)87271-9.

[ref80] Piccardo M.; Bloino J.; Barone V. Generalized vibrational perturbation theory for rotovibrational energies of linear, symmetric and asymmetric tops: Theory, approximations, and automated approaches to deal with medium-to-large molecular systems. Int. J. Quantum Chem. 2015, 115, 948–982. 10.1002/qua.24931. 26345131 PMC4553754

[ref81] Frisch M. J.; Trucks G. W.; Schlegel H. B.; Scuseria G. E.; Robb M. A.; Cheeseman J. R.; Scalmani G.; Barone V.; Petersson G. A.; Nakatsuji H. Gaussian 16, Revision C.01; 2016.

[ref82] Watson J. K. G. Determination of Centrifugal Distortion Coefficients of Asymmetric-Top Molecules. J. Chem. Phys. 1967, 46, 1935–1949. 10.1063/1.1840957.

[ref83] Watson J. K. Simplification of the molecular vibration-rotation hamiltonian. Mol. Phys. 1968, 15, 479–490. 10.1080/00268976800101381.

[ref84] Kraitchman J. Determination of Molecular Structure from Microwave Spectroscopic Data. Am. J. Phys. 1953, 21, 17–24. 10.1119/1.1933338.

[ref85] Kisiel Z. Spectroscopy from Space; Demaison J.; Sarka K.; Cohen E. A., Eds.; Springer: Netherlands, 2001; pp 91–106.

[ref86] Demaison J.; Boggs J. E.; Csaszar A. G. Equilibrium Molecular Structures; CRC Press, 2011.

[ref87] Oswald S.; Suhm M. A. Soft experimental constraints for soft interactions: a spectroscopic benchmark data set for weak and strong hydrogen bonds. Phys. Chem. Chem. Phys. 2019, 21, 18799 10.1039/C9CP03651B. 31453998

[ref88] Ruoff R. S.; Klots T. D.; Emilsson T.; Gutowsky H. S. Relaxation of conformers and isomers in seeded supersonic jets of inert gases. J. Chem. Phys. 1990, 93, 3142–3150. 10.1063/1.458848.

[ref89] Van Wynsberghe A. W.; Peebles S. A.; Peebles R. A.; Kuczkowski R. L. Rotational Spectrum and Structure of 1,2-Dichloro-3,3,4,4-tetrafluorocyclobutene: Comparison of Spectroscopy, Diffraction, and ab Initio Results. J. Phys. Chem. A 2000, 104, 8702–8708. 10.1021/jp001857l.

[ref90] Demaison J.; Császár A. G.; Margulès L. D.; Rudolph H. D. Equilibrium Structures of Heterocyclic Molecules with Large Principal Axis Rotations upon Isotopic Substitution. J. Phys. Chem. A 2011, 115, 14078–14091. 10.1021/jp2063595. 22032750

[ref91] Császár A. G.; Demaison J.; Rudolph H. D. Equilibrium Structures of Three-, Four-, Five-, Six-, and Seven-Membered Unsaturated N-Containing Heterocycles. J. Phys. Chem. A 2015, 119, 1731–1746. 10.1021/jp5084168. 25340501

[ref92] Kuczkowski R. L.; Gillies C.; Gallaher K. Microwave spectrum and structure of ethylene ozonide: Effects of large axes rotations in structure calculations. J. Mol. Spectrosc. 1976, 60, 361–372. 10.1016/0022-2852(76)90139-9.

[ref93] Wilson A. K.; Woon D. E.; Peterson K. A.; Dunning T. H. Gaussian basis sets for use in correlated molecular calculations. IX. The atoms gallium through krypton. J. Chem. Phys. 1999, 110, 7667–7676. 10.1063/1.478678.

[ref94] Woon D. E.; Dunning T. H. Gaussian basis sets for use in correlated molecular calculations. V. Core-valence basis sets for boron through neon. J. Chem. Phys. 1995, 103, 4572–4585. 10.1063/1.470645.

[ref95] Prascher B. P.; Woon D. E.; Peterson K. A.; Dunning T. H.; Wilson A. K. Gaussian basis sets for use in correlated molecular calculations. VII. Valence, core-valence, and scalar relativistic basis sets for Li, Be, Na, and Mg. Theor. Chem. Acc. 2011, 128, 69–82. 10.1007/s00214-010-0764-0.

[ref96] Woon D. E.; Dunning T. H. Calculation of the electron affinities of the second row atoms: Al-Cl. J. Chem. Phys. 1993, 99, 3730–3737. 10.1063/1.466148.

[ref97] Dunning T. H. Gaussian basis sets for use in correlated molecular calculations. I. The atoms boron through neon and hydrogen. J. Chem. Phys. 1989, 90, 1007–1023. 10.1063/1.456153.

[ref98] Kendall R. A.; Dunning T. H.; Harrison R. J. Electron affinities of the first-row atoms revisited Systematic basis sets and wave functions. J. Chem. Phys. 1992, 96, 6796–6806. 10.1063/1.462569.

[ref99] Woon D. E.; Dunning T. H. Gaussian basis sets for use in correlated molecular calculations. III. The atoms aluminum through argon. J. Chem. Phys. 1993, 98, 1358–1371. 10.1063/1.464303.

