# Supplementary material for: Varying Projection Quality of Good Local Electric Field Gradients of Monochlorobenzaldehydes
Source: J Phys Chem A. 2025 Jan 17;129(4):860–73. doi: 10.1021/acs.jpca.4c04915 (PMC11789141; doi:10.1021/acs.jpca.4c04915)
Supplement: Supplementary file 1 — jp4c04915_si_001.pdf [file jp4c04915_si_001.pdf]

Supporting information for 'Varying projection quality of good local electric field gradients of mono-chlorobenzaldehydes'

Robin Dohmen, Sean Arnold, Jessica Garrett, Beate Kempken, Beppo Hartwig, Benjamin Schröder, Pablo Pinacho, Melanie Schnell, Gordon G. Brown, and Daniel A. Obenchain

Contents

1 Contents of the electronic dataset S2

2 QCUMBER: Balle-Flygare type cavity Fourier transform microwave spectrometer S3

3 Relaxed scan of formyl torsional angle S4

4 Fitted structural information of 4-ClBzA S5

5 Hyperfine structure of 2-^{37}ClBzA S8

6 Experimental Transitions S10

6.1 4-ClBzA S10
6.2 3-ClBzA S35
6.3 2-ClBzA S50

7 NQCC calcultation S62

7.1 2-ClBzA S62
7.2 3-ClBzA S63
7.3 4-ClBzA S66

8 Raman Spectra S69

1 Contents of the electronic dataset

The folder ”calculation” contains all quantum chemical calculations performed in ORCA 5.0.4 and Gaussian 16 Rev C.01. Each isomer folder contains the output files for the equilibrium structure optimizations. An overview of the results, including rotational constants and NQCCs is found in each of the excel tables labeled ”isomer-results_table.xlsx”. The torsional scans and the results of the VPT2 analysis performed in Gaussian can be found in their respective folders.

The spectra from the cavity MW spectrometers is found in the folder labeled ”cavity spectra”. It contains data of the 2-, and 3-chlorobenzaldehyde. The 4-chlorobenzaldehyde was fitted exclusively with broadband data where additional lines were observed, hence we do not yet publish it in this data set. The dat files can be processed with the FTMW++ program by Jens-Uwe Grabow. All spectra containing transitions are also provided in txt format.

The ”pickett” folder contains a fit file and and a piform file for each species presented. This provides the line list found in Section 6 in txt format.

The ”STRFIT” folder contains the strfit output files and the xyz files from the structure fits, using a variety of starting geometries from theory. In addition, the Kraitchman calculations are also provided for each isomer.

This pdf document contains information on the experimental setup of the QCUMBER, select results of the structural fits performed, line lists for experimental transitions that were assigned, data on the NQCC and its transformation into the principal axis system and the nuclear axis system, and a Raman spectrum of 4-ClBzA and 2-ClBzA.

2 QCUMBER: Balle-Flygare type cavity Fourier transform microwave spectrometer

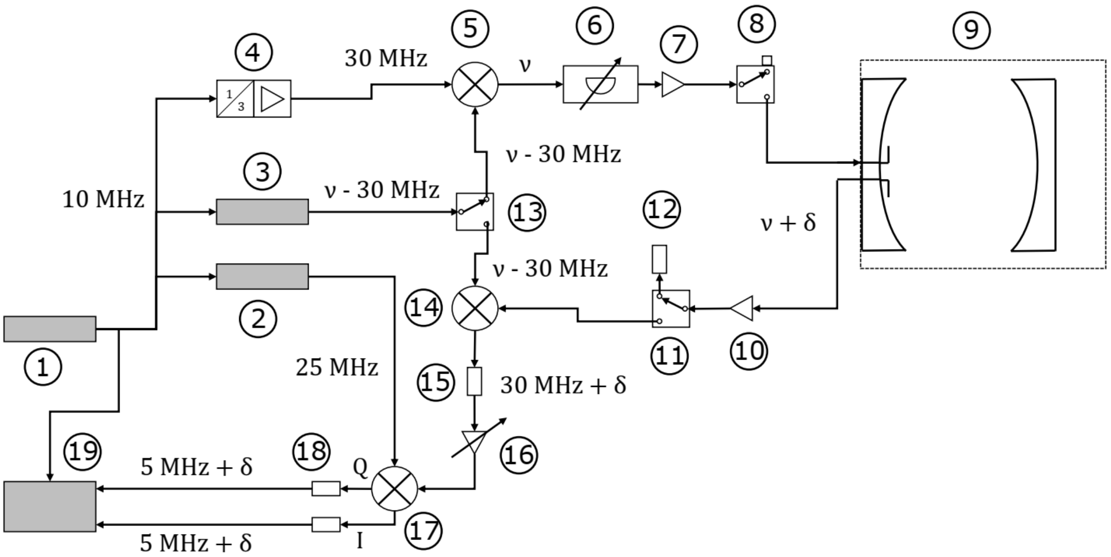

Figure S1: Scheme of the signal processing circuit. 1: Rb frequency generator; 2: radio frequency synthesizer; 3: microwave synthesizer; 4: frequency tripler; 5, 14: single sideband mixer; 6: variable attenuator; 7: power amplifier; 8, 11, 13: SPDT switch; 9: vacuum chamber; 10: low noise amplifier; 12: diode detector; 15: 30 MHz band pass filter (29-31 MHz); 16: variable amplifier; 17: I/Q demodulator; 18: 5 MHz low pass filters; 19: oscilloscope.

3 Relaxed scan of formyl torsional angle

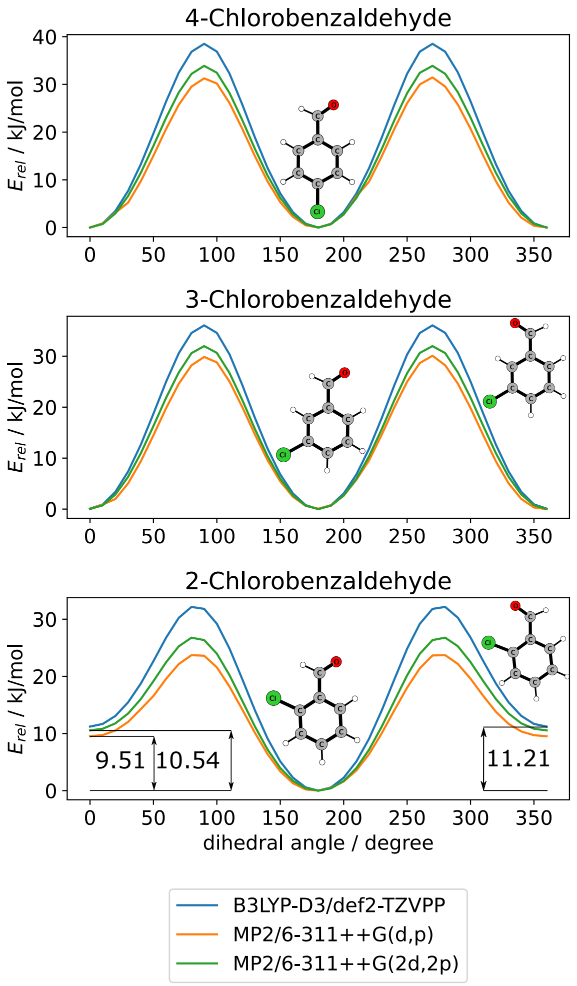

Figure S2: Relaxed scan curve of rotation around the aldehyde torsional angle describing the interconversion barrier from *cis* to *trans* chlorobenzaldehyde for the three molecules reported.

4 Fitted structural information of 4-ClBzA

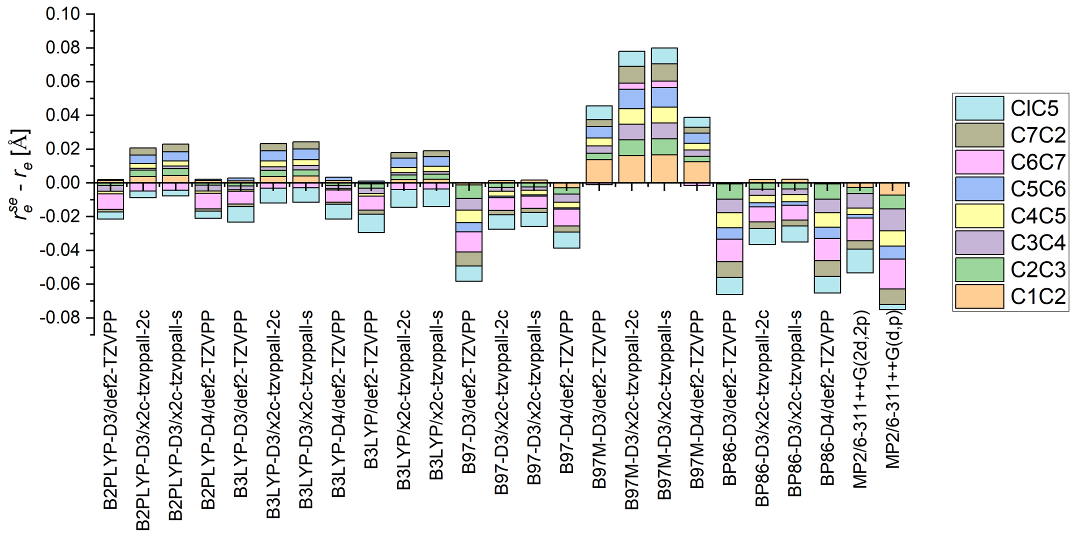

Figure S3: Comparison of r_e equilibirum geometries of 4-ClBzA at different levels of theory compared against an r_e^{se} structure that was obtained from B3LYP-D3/def2-TZVPP calculation.

Table S1: Cartesian coordinates of 4-ClBzA from kra calulation in / Å

atom label	a / Å	b / Å	c / Å
C1	2.94848 ± 0.00051	0.36782 ± 0.00409	0.03014 ± 0.04986
C2	1.46836 ± 0.00102	0.22192 ± 0.00677	0.01989 ± 0.07557
C3	0.87401 ± 0.00172	1.03805 ± 0.00145	0.02760 ± 0.05447
C4	0.48183 ± 0.00312	1.16064 ± 0.00129	0.01795 ± 0.08371
C5	1.27339 ± 0.00118	0.07195 ± 0.02088	0.02792 ± 0.05381
C6	0.70508 ± 0.00213	1.26575 ± 0.00119	0.02065 ± 0.07279
C7	0.66821 ± 0.00225	1.37421 ± 0.00109	0.03002 ± 0.05007
Cl	3.00711 ± 0.00050	0.15383 ± 0.00975	0.01365 ± 0.10995

Table S2: Cartesian coordinates for r_0 least square fit from AB rotational constants with B3LYP-D3/def2-TZVPP anharmonic rovibrational optimization of 4-ClBzA as a starting guess in / Å

atom label	a / Å	b / Å	c / Å
O	−3.728766	−0.555955	−0.000089
H	−3.315979	1.415492	−0.000118
C1	−2.950358	0.367936	0.000064
C2	−1.474512	0.227816	0.000039
C3	−0.879776	−1.041679	0.000045
C4	0.496412	−1.163902	0.000028
C5	1.279175	−0.007760	0.000002
C6	0.713916	1.269107	−0.000007
C7	−0.673778	1.378042	0.000011
Cl	3.008267	−0.152780	−0.000021
H	−1.518231	−1.914515	0.000067
H	0.970471	−2.134509	0.000034
H	−1.137637	2.361724	0.000006
H	1.342795	2.137453	−0.000027

Table S3: Cartesian coordinates of 4-ClBzA for $r_m^{(1)}$ least square fit with B3LYP-D3/def2-TZVPP equilibrium structure calculation as a starting guess in / Å

atom label	a / Å	b / Å	c / Å
O	−3.732813	−0.555633	−0.000012
H	−3.311417	1.413311	0.000001
C1	−2.950398	0.364878	0.000006
C2	−1.470423	0.233897	0.000005
C3	−0.876511	−1.041538	0.000007
C4	0.493768	−1.161932	0.000004
C5	1.277003	−0.006405	0.000000
C6	0.712679	1.266931	−0.000001
C7	−0.669950	1.376810	0.000002
Cl	3.008048	−0.153903	−0.000003
H	−1.514886	−1.914251	0.000010
H	0.968731	−2.132043	0.000006
H	−1.135895	2.359544	0.000001
H	1.346130	2.136231	−0.000004

Table S4: Cartesian coordinates of 4-ClBzA for r_e^{se} least square fit using B3LYP-D3/def2-TZVPP anharmonic rovibrational optimization for α_e correction in / Å

atom label	a / Å	b / Å	c / Å
O	−3.714658	−0.564134	−0.000088
H	−3.315927	1.410204	−0.000118
C1	−2.942852	0.365279	0.000064
C2	−1.472173	0.227425	0.000038
C3	−0.871936	−1.032989	0.000045
C4	0.503193	−1.155934	0.000028
C5	1.278015	0.001667	0.000002
C6	0.697330	1.265559	−0.000007
C7	−0.677870	1.371613	0.000011
Cl	3.000880	−0.153754	−0.000021
H	−1.504856	−1.909847	0.000067
H	0.976681	−2.126821	0.000034
H	−1.147403	2.356822	0.000005
H	1.334014	2.143363	−0.000027

5 Hyperfine structure of 2-^{37}ClBzA

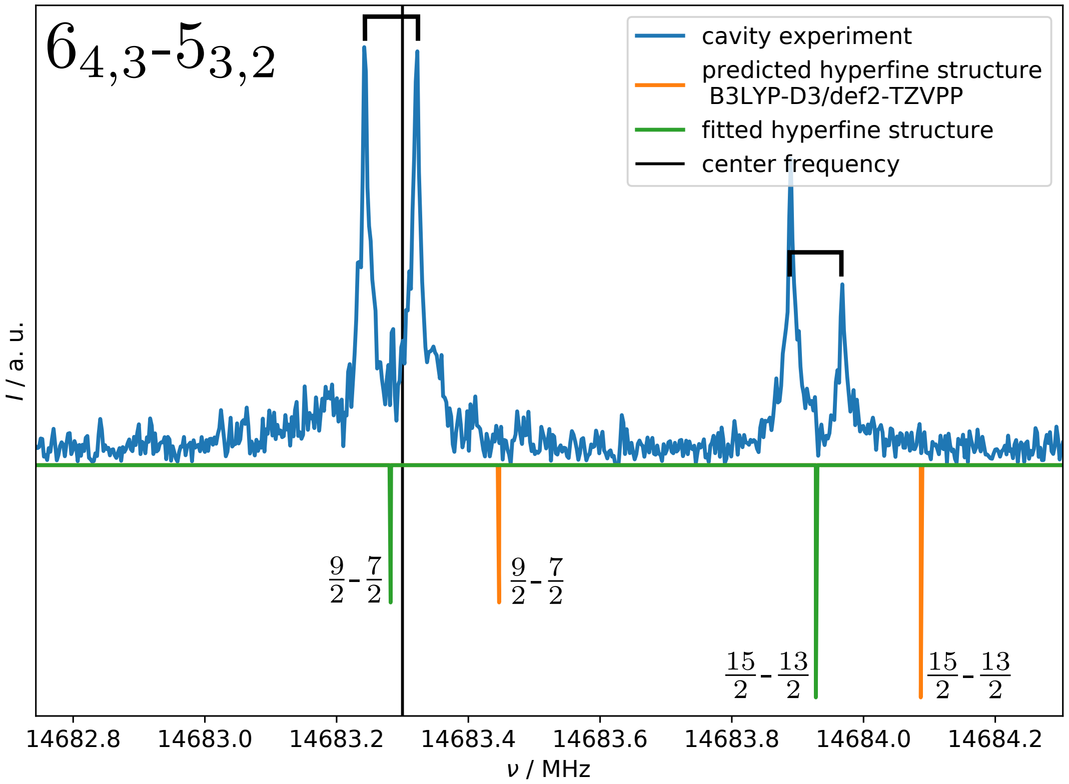

Figure S4: Rotational hyperfine splitting of chlorine as observed in 2-^{37}ClBzA at the QCUMBER spectrometer. It is the analous transition to the ones shown in Figure 5 in the main work, however, due to the difference in projection, the splitting is larger and hence it is impossible to observe the entire hyperfine pattern within the spectral window of the QCUMBER. Above in blue is the experimental spectrum, each peak appears as a Doppler doublet. Orange shows the hyperfine splitting as predicted (B3LYP-D4/def2-TZVPP) where the experimental rotational constants and centrifugal distortion parameters were used in the Hamiltonian. The green plot shows a line spectrum using all the fitted experimental parameters, including an experimental fit of χ_{aa}, χ_{bb-cc}, and χ_{ac}. The rotational quantum numbers J_{K_a,K_c} are given in the top right, while each hyperfine transition is labeled with its assigned $F = J + I$ quantum numbers. The intensity of the each transition is higher if it is close to the excitation frequency, which cause for the apparent weakness of the $\frac{15}{2} \leftarrow \frac{13}{2}$ transition. Upon centering that transition, it is stronger than the $\frac{9}{2} \leftarrow \frac{7}{2}$ transition, which can be found in the raw measurement data in the Gro.Data database.

6 Experimental Transitions

6.1 4-ClBzA

Table S5: Assignment in Pickett notation of experimentally determined transitions of 4-^{35}ClBzA

J'	K_a'	K_c'	F'	J''	K_a''	K_c''	F''	ν_{obs} / MHz	ν_{calc} / MHz	$\nu_{obs - calc}$ / MHz
3	1	2	3	3	0	3	2	4644.23855	4644.24060	−0.00205
3	1	2	4	3	0	3	5	4645.95381	4645.95640	−0.00258
3	1	2	5	3	0	3	4	4677.55015	4677.54903	0.00112
3	1	2	2	3	0	3	3	4675.86381	4675.86352	0.00029
3	1	2	3	3	0	3	4	4670.13049	4670.13009	0.0004
4	1	3	5	4	0	4	5	4838.00049	4837.99946	0.00103
4	1	3	4	4	0	4	4	4837.33469	4837.33361	0.00108
4	1	3	6	4	0	4	6	4836.15148	4836.15164	−0.00016
4	1	3	3	4	0	4	3	4835.50695	4835.50773	−0.00078
4	1	3	5	4	0	4	4	4831.78749	4831.78757	−0.00008
4	1	3	5	4	0	4	6	4820.40940	4820.41232	−0.00292
4	1	3	6	4	0	4	5	4853.73962	4853.73878	0.00084
4	1	3	3	4	0	4	4	4853.09934	4853.10113	−0.00179
4	1	3	4	4	0	4	5	4843.54583	4843.54550	0.00033
4	1	3	4	4	0	4	3	4819.73779	4819.74020	−0.00241
3	1	2	4	3	0	3	3	4655.29103	4655.29182	−0.00079
3	1	2	2	3	0	3	2	4658.26600	4658.26723	−0.00124
3	1	2	5	3	0	3	5	4659.92035	4659.92041	−0.00006
3	1	2	3	3	0	3	3	4661.83415	4661.83689	−0.00274
3	1	2	4	3	0	3	4	4663.58241	4663.58502	−0.00261
2	1	1	2	2	0	2	1	4514.82790	4514.82708	0.00082
2	1	1	3	2	0	2	4	4520.60060	4520.60112	−0.00053
2	1	1	3	2	0	2	2	4525.53824	4525.53652	0.00172
2	1	1	1	2	0	2	1	4524.45432	4524.45578	−0.00146
2	1	1	4	2	0	2	4	4530.13952	4530.13033	0.00919
2	1	1	2	2	0	2	2	4532.39203	4532.40365	−0.01161
2	1	1	3	2	0	2	3	4538.24054	4538.24514	−0.0046
2	1	1	1	2	0	2	2	4542.03203	4542.03234	−0.00032
2	1	1	4	2	0	2	3	4547.77501	4547.77435	0.00066
2	1	1	2	2	0	2	3	4545.11096	4545.11227	−0.00131
3	1	2	3	2	1	1	3	4030.51771	4030.51864	−0.00093
3	1	2	2	2	1	1	2	4037.67871	4037.67814	0.00058
3	1	2	5	2	1	1	4	4028.40491	4028.40837	−0.00346
3	1	2	2	2	1	1	1	4028.04655	4028.04944	−0.00289
3	1	2	4	2	1	1	3	4023.97625	4023.97357	0.00268
3	1	2	3	2	1	1	2	4023.65283	4023.65151	0.00132

Table S5: Assignment in Pickett notation of experimentally determined transitions of 4-^{35}ClBzA

J'	K_a'	K_c'	F'	J''	K_a''	K_c''	F''	ν_{obs} / MHz	ν_{calc} / MHz	$\nu_{obs - calc}$ / MHz
3	1	2	4	2	1	1	4	4014.44092	4014.44436	−0.00344
3	2	1	2	2	2	0	1	3924.95968	3924.96040	−0.00072
3	0	3	2	2	0	2	3	3924.52292	3924.52317	−0.00026
3	2	2	2	2	2	1	1	3920.23522	3920.23834	−0.00312
6	0	6	6	5	1	5	5	3946.84821	3946.83480	0.01341
6	0	6	7	5	1	5	6	3946.97829	3946.96339	0.0149
6	0	6	5	5	1	5	4	3949.19749	3949.20332	−0.00583
6	0	6	8	5	1	5	7	3949.34222	3949.34589	−0.00367
3	0	3	2	2	0	2	2	3911.81513	3911.81455	0.00058
3	2	2	5	2	2	1	4	3907.55028	3907.54997	0.00032
3	2	1	3	2	2	0	2	3907.24418	3907.24288	0.0013
3	0	3	3	2	0	2	3	3906.92572	3906.92689	−0.00117
3	2	2	2	2	2	1	2	3902.64611	3902.64451	0.0016
3	2	2	3	2	2	1	2	3902.54803	3902.54540	0.00263
3	2	1	4	2	2	0	3	3894.61014	3894.60705	0.00309
3	0	3	3	2	0	2	2	3894.20513	3894.21827	−0.01314
3	0	3	2	2	0	2	1	3894.24993	3894.23799	0.01195
3	2	2	4	2	2	1	3	3889.91993	3889.91728	0.00265
3	0	3	4	2	0	2	4	3880.98592	3880.98967	−0.00376
3	1	3	2	2	1	2	2	3787.01351	3787.01494	−0.00143
3	1	3	3	2	1	2	3	3780.30666	3780.30550	0.00116
3	1	3	2	2	1	2	1	3778.88745	3778.88141	0.00604
3	1	3	4	2	1	2	4	3766.41903	3766.42073	−0.00171
8	2	6	10	9	1	9	11	3680.05801	3680.06136	−0.00335
8	2	6	7	9	1	9	8	3680.22602	3680.22204	0.00398
6	2	5	7	7	1	6	8	2977.68867	2977.68447	0.00421
6	2	5	6	7	1	6	7	2977.10828	2977.10427	0.00402
6	2	5	8	7	1	6	9	2972.85879	2972.85490	0.00389
6	2	5	5	7	1	6	6	2972.30724	2972.30345	0.00379
2	1	1	2	1	1	0	1	2693.03625	2693.03522	0.00102
2	1	1	4	1	1	0	3	2689.20252	2689.20376	−0.00124
2	1	1	1	1	1	0	2	2688.10079	2688.09687	0.00392
2	1	1	2	1	1	0	3	2686.53836	2686.54167	−0.00331
2	1	1	3	1	1	0	3	2679.67508	2679.67454	0.00053
2	1	1	2	1	1	0	2	2678.46751	2678.46817	−0.00067
2	1	1	3	1	1	0	2	2671.60097	2671.60105	−0.00008
2	0	2	1	1	0	1	2	2632.29162	2632.29086	0.00076
2	0	2	2	1	0	1	2	2614.71037	2614.71430	−0.00393
2	0	2	4	1	0	1	3	2602.00486	2602.00421	0.00064
2	0	2	1	1	0	1	1	2600.46098	2600.46076	0.00022

Table S5: Assignment in Pickett notation of experimentally determined transitions of 4-^{35}ClBzA

J'	K_a'	K_c'	F'	J''	K_a''	K_c''	F''	ν_{obs} / MHz	ν_{calc} / MHz	$\nu_{\text{obs - calc}}$ / MHz
2	0	2	2	1	0	1	3	2597.06206	2597.06881	−0.00676
2	0	2	3	1	0	1	3	2584.36186	2584.36019	0.00167
2	0	2	2	1	0	1	1	2582.88538	2582.88419	0.00119
2	1	2	1	1	1	1	1	2536.35253	2536.35307	−0.00054
2	1	2	2	1	1	1	1	2528.21845	2528.21954	−0.0011
2	1	2	4	1	1	1	3	2522.75780	2522.75616	0.00164
2	1	2	2	1	1	1	2	2510.96415	2510.96389	0.00026
2	1	2	3	1	1	1	2	2505.15552	2505.15472	0.0008
5	0	5	4	4	1	4	4	2491.10712	2491.10744	−0.00032
5	0	5	5	4	1	4	5	2478.58338	2478.58484	−0.00146
5	0	5	7	4	1	4	6	2476.90659	2476.90833	−0.00174
5	0	5	4	4	1	4	3	2476.84453	2476.83998	0.00455
5	0	5	6	4	1	4	6	2459.35210	2459.35577	−0.00367
2	1	1	1	1	1	0	1	2702.66553	2702.66392	0.00161
3	1	3	3	2	1	2	4	3772.28497	3772.28451	0.00046
3	1	3	4	2	1	2	3	3774.44179	3774.44172	0.00007
3	1	3	3	2	1	2	2	3774.49521	3774.49633	−0.00112
3	2	1	2	2	2	0	2	3907.38369	3907.38341	0.00028
6	0	6	5	5	1	5	5	3964.34212	3964.34097	0.00115
3	1	2	3	2	1	1	4	4020.98501	4020.98942	−0.00442
3	1	2	2	2	1	1	3	4044.54799	4044.54527	0.00272
1	1	0	1	1	0	1	1	4422.25971	4422.25262	0.00709
1	1	0	2	1	0	1	1	4436.82439	4436.81967	0.00472
1	1	0	3	1	0	1	3	4442.92948	4442.93079	−0.00131
1	1	0	2	1	0	1	3	4451.00638	4451.00429	0.00209
1	1	0	1	1	0	1	2	4454.07981	4454.08272	−0.00291
1	1	0	3	1	0	1	2	4460.57559	4460.57627	−0.00068
1	1	0	2	1	0	1	2	4468.65078	4468.64977	0.001
7	2	5	9	8	1	8	10	4492.24993	4492.25267	−0.00274
5	2	4	4	6	1	5	5	4552.23044	4552.22957	0.00087
5	2	4	7	6	1	5	8	4553.26457	4553.26329	0.00128
5	2	4	5	6	1	5	6	4558.73319	4558.73152	0.00167
5	2	4	6	6	1	5	7	4559.81263	4559.81201	0.00062
4	1	4	5	3	1	3	5	5021.45778	5021.45915	−0.00136
4	1	4	4	3	1	3	3	5033.09482	5033.09342	0.0014
4	1	4	5	3	1	3	4	5033.92772	5033.92698	0.00075
4	1	4	3	3	1	3	2	5034.84473	5034.84227	0.00245
4	1	4	6	3	1	3	5	5035.70452	5035.70149	0.00302
4	1	4	4	3	1	3	4	5038.95284	5038.95719	−0.00436
4	1	4	3	3	1	3	3	5047.35846	5047.36088	−0.00242

Table S5: Assignment in Pickett notation of experimentally determined transitions of 4-^{35}ClBzA

J'	K_a'	K_c'	F'	J''	K_a''	K_c''	F''	ν_{obs} / MHz	ν_{calc} / MHz	$\nu_{obs - calc}$ / MHz
5	1	4	6	5	0	5	5	5058.51505	5058.51124	0.00381
5	1	4	4	5	0	5	4	5062.30730	5062.30504	0.00226
5	1	4	7	5	0	5	7	5062.56509	5062.56486	0.00023
5	1	4	5	5	0	5	5	5063.21957	5063.22221	−0.00264
5	1	4	6	5	0	5	6	5063.49686	5063.49797	−0.00111
5	1	4	5	5	0	5	6	5068.20546	5068.20894	−0.00348
5	1	4	7	5	0	5	6	5080.11817	5080.11742	0.00076
5	1	4	4	5	0	5	5	5079.85735	5079.85785	−0.0005
4	0	4	5	3	0	3	5	5174.49650	5174.49630	0.0002
4	0	4	3	3	0	3	2	5190.04010	5190.04074	−0.00064
4	2	3	5	3	2	2	4	5197.88702	5197.88688	0.00014
4	0	4	4	3	0	3	4	5198.33593	5198.33682	−0.00089
4	2	3	4	3	2	2	3	5200.39287	5200.39425	−0.00138
4	2	3	6	3	2	2	5	5204.97436	5204.97588	−0.00152
4	2	3	3	3	2	2	2	5207.41231	5207.41326	−0.00095
4	0	4	3	3	0	3	3	5207.63624	5207.63703	−0.00079
4	2	2	5	3	2	1	4	5209.62133	5209.61902	0.00231
4	2	2	4	3	2	1	5	5212.13660	5212.13514	0.00146
4	3	1	6	3	3	0	5	5210.82340	5210.82176	0.00164
4	3	2	6	3	3	1	5	5210.75604	5210.75917	−0.00313
4	2	2	6	3	2	1	5	5216.73620	5216.73918	−0.00298
4	2	2	3	3	2	1	2	5219.19063	5219.18743	0.00321
4	2	2	3	3	2	1	3	5219.32872	5219.32796	0.00076
6	1	5	7	6	0	6	8	5326.34004	5326.34367	−0.00362
6	1	5	6	6	0	6	5	5326.24504	5326.24386	0.00118
6	1	5	7	6	0	6	6	5339.68489	5339.68214	0.00276
6	1	5	5	6	0	6	5	5343.37207	5343.36870	0.00337
6	1	5	8	6	0	6	8	5343.46008	5343.45894	0.00114
6	1	5	6	6	0	6	6	5343.75241	5343.75003	0.00238
6	1	5	7	6	0	6	7	5343.85256	5343.84970	0.00286
6	1	5	6	6	0	6	7	5347.91705	5347.91760	−0.00054
4	1	3	5	3	1	2	5	5352.56663	5352.57536	−0.00873
4	1	3	4	3	1	2	3	5365.54000	5365.54034	−0.00035
4	1	3	5	3	1	2	4	5366.54041	5366.53937	0.00104
4	1	3	3	3	1	2	2	5367.28242	5367.28124	0.00118
4	1	3	6	3	1	2	5	5368.31792	5368.31468	0.00325
4	1	3	4	3	1	2	4	5372.08908	5372.08541	0.00367
4	1	3	3	3	1	2	3	5381.30858	5381.30787	0.00071
6	2	4	5	7	1	7	6	5381.54382	5381.54169	0.00213
6	2	4	8	7	1	7	9	5381.75594	5381.75443	0.00151

Table S5: Assignment in Pickett notation of experimentally determined transitions of 4-^{35}ClBzA

J'	K'_a	K'_c	F'	J''	K''_a	K''_c	F''	ν_{obs} / MHz	ν_{calc} / MHz	$\nu_{obs - calc}$ / MHz
6	2	4	6	7	1	7	7	5384.68756	5384.68669	0.00087
6	2	4	7	7	1	7	8	5384.92860	5384.92854	0.00006
7	0	7	8	6	1	6	7	5441.54234	5441.54251	−0.00017
7	0	7	7	6	1	6	6	5441.39543	5441.39622	−0.00079
7	0	7	6	6	1	6	5	5443.21865	5443.22078	−0.00213
7	0	7	9	6	1	6	8	5443.37203	5443.37312	−0.00109
7	0	7	7	6	1	6	7	5445.12081	5445.12097	−0.00016
1	1	1	1	0	0	0	2	5656.20137	5656.20066	0.00071
1	1	1	3	0	0	0	2	5663.87630	5663.87587	0.00043
1	1	1	2	0	0	0	2	5673.45829	5673.45632	0.00196
7	1	6	8	7	0	7	7	5680.13657	5680.13629	0.00028
7	1	6	7	7	0	7	8	5687.28373	5687.28153	0.00219
7	1	6	7	7	0	7	8	5687.28453	5687.28153	0.003
7	1	6	9	7	0	7	8	5701.13182	5701.13254	−0.00072
8	1	7	10	8	0	8	10	6088.48952	6088.47824	0.01128
8	1	7	9	8	0	8	9	6088.26808	6088.25642	0.01167
4	2	3	4	5	1	4	4	6086.11899	6086.11705	0.00194
8	1	7	9	8	0	8	8	6085.11892	6085.12144	−0.00252
4	2	3	3	5	1	4	4	6093.23642	6093.23516	0.00126
4	2	3	6	5	1	4	7	6095.40926	6095.40459	0.00467
4	2	3	5	5	1	4	5	6100.29544	6100.29141	0.00402
5	1	5	6	4	1	4	6	6276.72261	6276.72236	0.00025
5	1	5	5	4	1	4	4	6290.23024	6290.23064	−0.00041
5	1	5	6	4	1	4	5	6290.96528	6290.96470	0.00058
5	1	5	4	4	1	4	3	6291.10078	6291.10083	−0.00005
5	1	5	7	4	1	4	6	6291.84633	6291.84589	0.00044
5	1	5	5	4	1	4	5	6295.26410	6295.26086	0.00324
4	2	3	5	5	1	4	6	6105.00078	6105.00239	−0.00161
5	1	5	4	4	1	4	4	6305.36887	6305.36829	0.00057
5	2	3	6	6	1	6	7	6345.35243	6345.35414	−0.00171
5	2	3	5	6	1	6	6	6344.64554	6344.64846	−0.00293
5	0	5	6	4	0	4	6	6463.47140	6463.47490	−0.0035
5	0	5	7	4	0	4	6	6481.03244	6481.02746	0.00498
5	0	5	5	4	0	4	4	6479.82324	6479.83687	−0.01363
5	0	5	5	4	0	4	5	6486.04834	6486.04877	−0.00043
5	2	4	6	4	2	3	6	6492.46563	6492.46969	−0.00406
5	0	5	4	4	0	4	4	6497.39117	6497.38969	0.00149
5	4	1	6	4	4	0	5	6497.56288	6497.56253	0.00035
5	2	4	6	4	2	3	5	6499.48973	6499.49134	−0.00161
5	2	4	5	4	2	3	4	6500.01517	6500.01748	−0.00231

Table S5: Assignment in Pickett notation of experimentally determined transitions of 4-^{35}ClBzA

J'	K'_a	K'_c	F'	J''	K''_a	K''_c	F''	ν_{obs} / MHz	ν_{calc} / MHz	$\nu_{obs - calc}$ / MHz
5	2	4	5	4	2	3	5	6502.48141	6502.47875	0.00267
5	2	4	7	4	2	3	6	6503.03859	6503.03624	0.00235
5	3	2	6	4	3	1	5	6503.26261	6503.26191	0.0007
5	2	4	4	4	2	3	3	6503.52321	6503.52225	0.00096
5	3	2	5	4	3	1	4	6505.95065	6505.95224	−0.00159
5	3	3	5	4	3	2	4	6505.73302	6505.73400	−0.00097
5	2	4	4	4	2	3	4	6510.64828	6510.64036	0.00792
5	3	3	7	4	3	2	6	6511.02020	6511.01736	0.00284
5	3	2	7	4	3	1	6	6511.23730	6511.23622	0.00108
5	4	2	7	4	4	1	6	6511.68094	6511.68018	0.00076
5	3	3	4	4	3	2	3	6513.66057	6513.66622	−0.00565
5	3	2	4	4	3	1	3	6513.87806	6513.88527	−0.00721
5	2	3	6	4	2	2	6	6515.84677	6515.84275	0.00401
5	2	3	6	4	2	2	5	6522.93371	6522.93653	−0.00282
5	2	3	5	4	2	2	4	6523.46453	6523.46586	−0.00133
5	2	3	7	4	2	2	6	6526.52622	6526.52433	0.00189
5	2	3	4	4	2	2	3	6527.00942	6527.01136	−0.00193
5	2	3	5	4	2	2	5	6525.95336	6525.95560	−0.00224
9	1	8	9	9	0	9	9	6562.89231	6562.89415	−0.00184
9	1	8	11	9	0	9	11	6563.23786	6563.24428	−0.00642
9	1	8	8	9	0	9	8	6563.35131	6563.35713	−0.00582
5	1	4	6	4	1	3	6	6690.81943	6690.82123	−0.0018
5	1	4	5	4	1	3	4	6705.72320	6705.72548	−0.00228
5	1	4	6	4	1	3	5	6706.56456	6706.56055	0.00402
5	1	4	7	4	1	3	6	6707.44199	6707.44068	0.00131
5	1	4	5	4	1	3	5	6711.27230	6711.27152	0.00078
5	1	4	4	4	1	3	4	6722.36112	6722.36112	0.0000
2	1	2	2	1	0	1	1	6865.83895	6865.83858	0.00037
2	1	2	1	1	0	1	1	6873.98359	6873.97211	0.01148
2	1	2	3	1	0	1	3	6874.22296	6874.21404	0.00892
2	1	2	2	1	0	1	3	6880.02169	6880.02320	−0.00152
2	1	2	4	1	0	1	3	6882.23228	6882.23503	−0.00275
2	1	2	3	1	0	1	2	6891.85446	6891.85952	−0.00506
2	1	2	2	1	0	1	2	6897.66679	6897.66869	−0.0019
2	1	2	1	1	0	1	2	6905.80223	6905.80221	0.00001
8	0	8	8	7	1	7	7	6951.33772	6951.33790	−0.00019
8	0	8	9	7	1	7	8	6951.47085	6951.47019	0.00066
8	0	8	7	7	1	7	6	6952.80279	6952.79716	0.00564
8	0	8	10	7	1	7	9	6952.93923	6952.93478	0.00445
8	0	8	7	7	1	7	7	6968.72742	6968.72819	−0.00077

Table S5: Assignment in Pickett notation of experimentally determined transitions of 4-^{35}ClBzA

J'	K_a'	K_c'	F'	J''	K_a''	K_c''	F''	ν_{obs} / MHz	ν_{calc} / MHz	$\nu_{obs - calc}$ / MHz
0	1	9	11	10	0	10	11	7112.61175	7112.61488	−0.00313
0	1	9	10	10	0	10	10	7112.68974	7112.69133	−0.00159
0	1	9	12	10	0	10	12	7113.18427	7113.18125	0.00302
0	1	9	9	10	0	10	9	7113.26989	7113.26535	0.00454
4	2	2	3	5	1	5	4	7358.96037	7358.96896	−0.00858
4	2	2	6	5	1	5	7	7360.68741	7360.69096	−0.00355
4	2	2	4	5	1	5	5	7366.91737	7366.91430	0.00307
4	2	2	5	5	1	5	6	7368.72803	7368.72072	0.00731
6	1	6	7	5	1	5	7	7531.18407	7531.17957	0.00449
6	1	6	8	5	1	5	7	7546.80084	7546.80051	0.00033
6	1	6	6	5	1	5	5	7545.73408	7545.73170	0.00238
6	1	6	5	5	1	5	4	7546.22321	7546.22133	0.00189
6	1	6	7	5	1	5	6	7546.30539	7546.30311	0.00228
6	1	6	6	5	1	5	6	7550.02589	7550.02786	−0.00197
6	1	6	5	5	1	5	5	7561.35711	7561.35897	−0.00187
3	2	2	2	4	1	3	3	7592.40456	7592.41549	−0.01093
3	2	2	5	4	1	3	6	7597.87077	7597.86938	0.00138
3	2	2	3	4	1	3	4	7608.08675	7608.08391	0.00284
3	2	2	4	4	1	3	5	7613.66598	7613.67605	−0.01008
6	0	6	7	5	0	5	7	7746.77548	7746.77742	−0.00193
6	0	6	6	5	0	5	5	7763.50784	7763.51082	−0.00298
6	0	6	7	5	0	5	6	7764.31854	7764.32998	−0.01144
6	0	6	6	5	0	5	6	7768.49737	7768.49755	−0.00017
6	2	5	7	5	2	4	6	7798.61911	7798.61973	−0.00062
6	2	5	8	5	2	4	7	7800.65106	7800.64141	0.00965
6	4	2	7	5	4	1	6	7803.95358	7803.95745	−0.00387
6	4	2	6	5	4	1	5	7806.36519	7806.36535	−0.00016
6	3	4	7	5	3	3	6	7808.43628	7808.44028	−0.00399
6	3	3	7	5	3	2	6	7809.01977	7809.02188	−0.00211
6	3	4	6	5	3	3	5	7809.44349	7809.45052	−0.00703
6	3	3	6	5	3	2	5	7810.02412	7810.03243	−0.00831
6	2	5	5	5	2	4	5	7811.23601	7811.24178	−0.00577
6	4	3	8	5	4	2	7	7812.03899	7812.04588	−0.00689
6	3	4	8	5	3	3	7	7813.00369	7813.00486	−0.00117
6	3	3	8	5	3	2	7	7813.58836	7813.58793	0.00044
6	4	2	6	5	4	1	7	7813.97947	7813.97205	0.00743
6	4	2	5	5	4	1	4	7814.44236	7814.43603	0.00633
6	3	3	5	5	3	2	4	7814.56636	7814.56440	0.00196
6	2	4	6	5	2	3	5	7839.53312	7839.52673	0.00639
6	2	4	8	5	2	3	7	7841.59726	7841.59044	0.00682

Table S5: Assignment in Pickett notation of experimentally determined transitions of 4-^{35}ClBzA

J'	K_a'	K_c'	F'	J''	K_a''	K_c''	F''	ν_{obs} / MHz	ν_{calc} / MHz	$\nu_{obs - calc}$ / MHz
5	2	3	4	6	1	6	5	6339.74906	6339.75899	−0.00993

Table S6: Assignment in Pickett notation of experimentally determined transitions of 4-^{37}ClBzA

J'	K_a'	K_c'	F'	J''	K_a''	K_c''	F''	ν_{obs} / MHz	ν_{calc} / MHz	$\nu_{obs - calc}$ / MHz
2	1	2	3	1	1	1	2	2453.03821	2453.03918	−0.00097
2	1	2	2	1	1	1	2	2457.61413	2457.61195	0.00218
2	1	2	4	1	1	1	3	2466.92653	2466.92718	−0.00065
2	0	2	4	1	0	1	3	2543.20374	2543.20734	−0.00360
2	0	2	1	1	0	1	1	2542.00563	2541.99376	0.01187
2	0	2	2	1	0	1	2	2553.21502	2553.21563	−0.00061
2	1	1	3	1	1	0	2	2612.15525	2612.15193	0.00332
2	1	1	2	1	1	0	2	2617.55993	2617.56375	−0.00382
2	1	1	3	1	1	0	3	2618.51422	2618.51636	−0.00214
2	1	1	4	1	1	0	3	2626.04102	2626.04036	0.00065
2	1	1	2	1	1	0	1	2629.04243	2629.04211	0.00031
2	1	2	3	1	1	1	3	2460.59159	2460.59603	−0.00443
2	1	2	1	1	1	1	2	2464.01296	2464.01484	−0.00188
2	1	2	2	1	1	1	3	2465.17613	2465.16879	0.00734
2	1	2	2	1	1	1	1	2471.22238	2471.22154	0.00083
2	1	2	1	1	1	1	1	2477.62447	2477.62444	0.00003
2	1	1	1	1	1	0	1	2636.63298	2636.63150	0.00149
3	1	3	3	2	1	2	3	3697.28595	3697.28535	0.00060
3	1	3	5	2	1	2	4	3696.16850	3696.16923	−0.00072
3	1	3	4	2	1	2	4	3686.33481	3686.33712	−0.00230
3	1	3	4	2	1	2	3	3692.68085	3692.66827	0.01258
3	1	3	2	2	1	2	2	3702.57373	3702.57703	−0.00330
3	0	3	4	2	0	2	4	3797.08841	3797.09132	−0.00290
3	2	2	4	2	2	1	3	3804.70638	3804.70338	0.00300
3	0	3	3	2	0	2	2	3807.52897	3807.52787	0.00109
3	2	1	4	2	2	0	3	3808.98362	3808.98119	0.00242
3	0	3	5	2	0	2	4	3810.99496	3810.99116	0.00380
3	2	2	3	2	2	1	2	3814.65950	3814.65885	0.00065
3	0	3	3	2	0	2	3	3817.53568	3817.53324	0.00244
3	2	2	5	2	2	1	4	3818.61714	3818.61119	0.00595
3	2	1	3	2	2	0	2	3818.94363	3818.94231	0.00132
3	2	1	2	2	2	0	2	3819.03508	3819.03645	−0.00137
3	0	3	2	2	0	2	2	3821.40873	3821.40913	−0.00041

Table S6: Assignment in Pickett notation of experimentally determined transitions of 4-^{37}ClBzA

J'	K'_a	K'_c	F'	J''	K''_a	K''_c	F''	ν_{obs} / MHz	ν_{calc} / MHz	$\nu_{obs - calc}$ / MHz
3	2	1	5	2	2	0	4	3822.90650	3822.90749	−0.00099
3	2	2	2	2	2	1	1	3828.60934	3828.60518	0.00416
3	2	1	2	2	2	0	1	3832.90460	3832.90709	−0.00248
3	1	2	4	2	1	1	4	3923.70316	3923.70531	−0.00216
3	1	2	4	2	1	1	3	3931.22681	3931.22931	−0.00251
3	1	2	3	2	1	1	2	3930.97795	3930.97671	0.00124
3	1	2	2	2	1	1	1	3934.44965	3934.44546	0.00419
3	1	2	5	2	1	1	4	3934.72520	3934.72269	0.00251
3	1	2	3	2	1	1	3	3936.38826	3936.38852	−0.00026
3	1	2	2	2	1	1	2	3942.03496	3942.03484	0.00012
2	1	1	2	2	0	2	1	4525.29951	4525.29791	0.00160
2	1	1	3	2	0	2	4	4529.85344	4529.84881	0.00463
2	1	1	4	2	0	2	4	4537.36967	4537.37281	−0.00314
2	1	1	2	2	0	2	2	4539.16935	4539.16835	0.00100
2	1	1	3	2	0	2	3	4543.76317	4543.76190	0.00127
2	1	1	1	2	0	2	2	4546.75873	4546.75773	0.00100
2	1	1	2	2	0	2	3	4549.17493	4549.17372	0.00122
2	1	1	4	2	0	2	3	4551.28822	4551.28590	0.00231
2	1	1	3	2	0	2	2	4533.74631	4533.75653	−0.01022
1	1	0	2	1	0	1	2	4474.82266	4474.82023	0.00243
1	1	0	3	1	0	1	2	4468.46108	4468.45580	0.00528
1	1	0	1	1	0	1	2	4463.34078	4463.34186	−0.00109
1	1	0	2	1	0	1	3	4460.90426	4460.90422	0.00004
1	1	0	3	1	0	1	3	4454.53906	4454.53979	−0.00073
1	1	0	2	1	0	1	1	4449.72782	4449.72792	−0.00010
1	1	0	1	1	0	1	1	4438.24920	4438.24956	−0.00036
3	1	2	3	3	0	3	2	4648.73507	4648.73592	−0.00085
3	1	2	4	3	0	3	5	4650.08566	4650.08696	−0.00130
3	1	2	4	3	0	3	3	4657.45960	4657.45797	0.00163
3	1	2	2	3	0	3	2	4659.79619	4659.79406	0.00213
3	1	2	5	3	0	3	5	4661.10182	4661.10434	−0.00252
3	1	2	3	3	0	3	3	4662.61513	4662.61718	−0.00205
3	1	2	4	3	0	3	4	4663.98714	4663.98681	0.00033
3	1	2	3	3	0	3	4	4669.14839	4669.14602	0.00237
3	1	2	2	3	0	3	3	4673.67772	4673.67532	0.00241
3	1	2	5	3	0	3	4	4675.00922	4675.00419	0.00503
7	2	5	8	8	1	8	9	4683.63563	4683.63136	0.00426
5	2	4	7	6	1	5	8	4803.63113	4803.62989	0.00124
5	2	4	4	6	1	5	5	4802.81163	4802.81012	0.00151
5	2	4	5	6	1	5	6	4807.94545	4807.94426	0.00118

Table S6: Assignment in Pickett notation of experimentally determined transitions of 4-^{37}ClBzA

J'	K'_a	K'_c	F'	J''	K''_a	K''_c	F''	ν_{obs} / MHz	ν_{calc} / MHz	$\nu_{obs - calc}$ / MHz
5	2	4	6	6	1	5	7	4808.79385	4808.79397	−0.00013
4	1	3	4	4	0	4	3	4816.14816	4816.14633	0.00183
4	1	3	5	4	0	4	6	4816.67077	4816.67241	−0.00164
4	1	3	5	4	0	4	4	4825.64886	4825.65061	−0.00175
4	1	3	3	4	0	4	3	4828.58002	4828.57937	0.00066
4	1	3	6	4	0	4	6	4829.08720	4829.08771	−0.00051
4	1	3	4	4	0	4	4	4830.02143	4830.02134	0.00009
4	1	3	5	4	0	4	5	4830.54270	4830.54253	0.00017
4	1	3	4	4	0	4	5	4834.91163	4834.91326	−0.00163
4	1	3	3	4	0	4	4	4842.45874	4842.45437	0.00437
4	1	3	6	4	0	4	5	4842.95865	4842.95782	0.00083
4	1	4	5	3	1	3	5	4914.60534	4914.60974	−0.00440
4	1	4	4	3	1	3	3	4923.78723	4923.78516	0.00207
4	1	4	5	3	1	3	4	4924.44407	4924.44185	0.00223
4	1	4	3	3	1	3	2	4925.16906	4925.16586	0.00320
4	1	4	6	3	1	3	5	4925.83945	4925.83889	0.00056
4	1	4	4	3	1	3	4	4928.40225	4928.40224	0.00001
4	1	4	3	3	1	3	3	4935.02441	4935.03031	−0.00590
5	1	4	6	5	0	5	5	5041.51064	5041.51094	−0.00031
5	1	4	5	5	0	5	6	5049.15105	5049.15102	0.00003
5	1	4	6	5	0	5	6	5045.43698	5045.43854	−0.00156
5	1	4	4	5	0	5	4	5044.50376	5044.49816	0.00560
5	1	4	5	5	0	5	4	5031.37792	5031.37955	−0.00163
5	1	4	6	5	0	5	7	5031.60233	5031.59461	0.00772
4	0	4	5	3	0	3	5	5061.98459	5061.98694	−0.00234
4	0	4	4	3	0	3	3	5074.24847	5074.24986	−0.00139
4	2	3	5	3	2	2	4	5081.77034	5081.77123	−0.00088
4	0	4	4	3	0	3	4	5080.77507	5080.77870	−0.00364
4	2	3	4	3	2	2	3	5083.74324	5083.74344	−0.00020
4	2	3	6	3	2	2	5	5087.35626	5087.35687	−0.00061
4	0	4	3	3	0	3	3	5088.12469	5088.12487	−0.00018
4	2	3	3	3	2	2	2	5089.28118	5089.28451	−0.00333
4	2	2	5	3	2	1	4	5092.47640	5092.47555	0.00085
4	2	2	4	3	2	1	3	5094.44959	5094.44948	0.00010
4	2	2	6	3	2	1	5	5098.08270	5098.08682	−0.00413
4	2	2	3	3	2	1	2	5100.01167	5100.01515	−0.00347
4	2	2	3	3	2	1	3	5100.10420	5100.10929	−0.00509
7	0	7	7	6	1	6	6	5197.33876	5197.33920	−0.00044
7	0	7	8	6	1	6	7	5197.44472	5197.44409	0.00063
7	0	7	6	6	1	6	5	5198.78461	5198.78549	−0.00088

Table S6: Assignment in Pickett notation of experimentally determined transitions of 4-^{37}ClBzA

J'	K_a'	K_c'	F'	J''	K_a''	K_c''	F''	ν_{obs} / MHz	ν_{calc} / MHz	$\nu_{obs - calc}$ / MHz
7	0	7	9	6	1	6	8	5198.89614	5198.89659	−0.00045
4	1	3	5	3	1	2	5	5231.42376	5231.42512	−0.00136
4	1	3	4	3	1	2	3	5241.65352	5241.65402	−0.00050
4	1	3	5	3	1	2	4	5242.44164	5242.44250	−0.00086
4	1	3	3	3	1	2	2	5243.02844	5243.02891	−0.00048
4	1	3	6	3	1	2	5	5243.84197	5243.84042	0.00155
4	1	3	4	3	1	2	4	5246.81136	5246.81323	−0.00187
4	1	3	3	3	1	2	3	5254.08456	5254.08705	−0.00249
6	1	5	5	6	0	6	5	5311.78896	5311.79796	−0.00900
6	1	5	8	6	0	6	8	5311.86789	5311.86956	−0.00167
6	1	5	6	6	0	6	6	5312.09955	5312.10122	−0.00167
6	1	5	7	6	0	6	7	5312.17467	5312.17895	−0.00428
6	1	5	6	6	0	6	7	5315.38074	5315.38406	−0.00332
6	1	5	5	6	0	6	6	5325.60328	5325.60659	−0.00332
6	1	5	8	6	0	6	7	5325.68047	5325.67829	0.00218
6	2	4	8	7	1	7	9	5563.72814	5563.72569	0.00245
6	2	4	6	7	1	7	7	5566.04124	5566.04139	−0.00015
6	2	4	7	7	1	7	8	5566.23152	5566.23064	0.00088
7	1	6	7	7	0	7	6	5621.29867	5621.28525	0.01342
7	1	6	8	7	0	7	7	5632.23655	5632.24269	−0.00613
7	1	6	9	7	0	7	9	5635.02583	5635.03256	−0.00673
1	1	1	1	0	0	0	2	5643.09671	5643.09703	−0.00032
1	1	1	3	0	0	0	2	5649.14935	5649.14978	−0.00042
1	1	1	2	0	0	0	2	5656.70780	5656.70662	0.00118
8	1	7	10	8	0	8	10	6019.02686	6019.03727	−0.01041
8	1	7	8	8	0	8	8	6018.89504	6018.89464	0.00040
5	1	5	6	4	1	4	6	6142.91138	6142.91642	−0.00504
5	1	5	5	4	1	4	4	6153.56706	6153.56750	−0.00045
5	1	5	6	4	1	4	5	6154.14501	6154.14557	−0.00056
5	1	5	4	4	1	4	3	6154.25193	6154.25448	−0.00255
5	1	5	7	4	1	4	6	6154.83648	6154.83955	−0.00307
5	1	5	5	4	1	4	5	6157.52741	6157.52790	−0.00049
5	1	5	4	4	1	4	4	6165.49963	6165.49964	0.00000
4	2	3	3	5	1	4	4	6304.66978	6304.67170	−0.00193
4	2	3	6	5	1	4	7	6306.38857	6306.38985	−0.00128
4	2	3	4	5	1	4	5	6312.18135	6312.18614	−0.00479
4	2	3	5	5	1	4	6	6313.95307	6313.95568	−0.00261
5	0	5	5	4	0	4	4	6335.64731	6335.64165	0.00566
5	0	5	7	4	0	4	6	6336.59257	6336.57979	0.01279
5	0	5	5	4	0	4	5	6340.52439	6340.53357	−0.00918

Table S6: Assignment in Pickett notation of experimentally determined transitions of 4-^{37}ClBzA

J'	K_a'	K_c'	F'	J''	K_a''	K_c''	F''	ν_{obs} / MHz	ν_{calc} / MHz	$\nu_{obs - calc}$ / MHz
5	4	1	6	4	4	0	5	6352.83181	6352.83222	−0.00041
5	2	4	6	4	2	3	5	6353.70012	6353.71193	−0.01181
5	2	4	5	4	2	3	4	6354.11374	6354.12439	−0.01066
5	2	4	7	4	2	3	6	6356.49851	6356.50449	−0.00598
5	2	4	4	4	2	3	3	6356.88551	6356.89144	−0.00594
5	3	2	6	4	3	1	5	6357.50719	6357.51441	−0.00723
5	3	3	5	4	3	2	4	6359.44024	6359.44242	−0.00217
5	3	2	5	4	3	1	4	6359.63325	6359.63214	0.00111
5	3	3	7	4	3	2	6	6363.60891	6363.60882	0.00009
5	3	2	7	4	3	1	6	6363.79857	6363.79898	−0.00042
5	3	3	4	4	3	2	3	6365.70134	6365.70018	0.00116
5	3	2	4	4	3	1	3	6365.89114	6365.89048	0.00066
5	2	3	6	4	2	2	5	6375.08825	6375.08686	0.00139
5	2	3	5	4	2	2	4	6375.51364	6375.50726	0.00638
5	2	3	5	4	2	2	5	6377.47064	6377.46621	0.00443
5	2	3	7	4	2	2	6	6377.91669	6377.90921	0.00748
5	2	3	4	4	2	2	3	6378.31224	6378.30490	0.00733
5	2	3	7	6	1	6	8	6510.43632	6510.43565	0.00067
5	2	3	4	6	1	6	5	6509.91754	6509.92047	−0.00293
5	2	3	5	6	1	6	6	6513.77744	6513.78432	−0.00688
5	2	3	6	6	1	6	7	6514.32001	6514.32924	−0.00923
5	1	4	6	4	1	3	6	6539.08263	6539.08669	−0.00406
5	1	4	7	4	1	3	6	6552.20719	6552.19522	0.01197
5	1	4	5	4	1	3	4	6550.85243	6550.84373	0.00870
5	1	4	4	4	1	3	4	6563.95733	6563.96235	−0.00502
8	0	8	8	7	1	7	7	6672.29576	6672.29228	0.00349
8	0	8	9	7	1	7	8	6672.39534	6672.39419	0.00116
8	0	8	7	7	1	7	6	6673.45613	6673.45451	0.00162
8	0	8	10	7	1	7	9	6673.56174	6673.56001	0.00173
2	1	2	2	1	0	1	1	6828.79278	6828.79370	−0.00092
2	1	2	1	1	0	1	1	6835.19703	6835.19660	0.00044
2	1	2	3	1	0	1	3	6835.39651	6835.39723	−0.00071
2	1	2	2	1	0	1	3	6839.96750	6839.96999	−0.00250
2	1	2	4	1	0	1	3	6841.72475	6841.72838	−0.00362
2	1	2	3	1	0	1	2	6849.31356	6849.31323	0.00033
2	1	2	2	1	0	1	2	6853.88872	6853.88600	0.00272
2	1	2	1	1	0	1	2	6860.28745	6860.28890	−0.00145
6	1	6	6	5	1	5	5	7381.87409	7381.87832	−0.00423
6	1	6	5	5	1	5	4	7382.26004	7382.26749	−0.00746
6	1	6	7	5	1	5	6	7382.33598	7382.33638	−0.00039

Table S6: Assignment in Pickett notation of experimentally determined transitions of 4-^{37}ClBzA

J'	K_a'	K_c'	F'	J''	K_a''	K_c''	F''	ν_{obs} / MHz	ν_{calc} / MHz	$\nu_{obs - calc}$ / MHz
6	1	6	8	5	1	5	7	7382.72343	7382.72833	−0.00490
4	2	2	3	5	1	5	4	7513.89035	7513.88306	0.00729
4	2	2	6	5	1	5	7	7515.26796	7515.25477	0.01319
4	2	2	4	5	1	5	5	7520.15849	7520.15538	0.00311
4	2	2	5	5	1	5	6	7521.57458	7521.57876	−0.00418
6	0	6	7	5	0	5	7	7578.29189	7578.28929	0.00260
1	1	10	12	11	0	11	12	7584.97098	7584.97594	−0.00496
1	1	10	11	11	0	11	11	7585.05203	7585.04426	0.00777
1	1	10	13	11	0	11	13	7585.51505	7585.51577	−0.00072
6	0	6	6	5	0	5	6	7595.40949	7595.41606	−0.00657
6	0	6	5	5	0	5	5	7605.29729	7605.29710	0.00019
6	2	5	7	5	2	4	6	7623.58392	7623.57928	0.00464
6	3	4	7	5	3	3	6	7632.74716	7632.74924	−0.00207
6	3	3	7	5	3	2	6	7633.25642	7633.25488	0.00154
6	3	4	6	5	3	3	5	7633.54048	7633.54327	−0.00279
6	3	3	6	5	3	2	5	7634.04942	7634.04913	0.00029
6	3	4	8	5	3	3	7	7636.34973	7636.34568	0.00405
6	3	3	8	5	3	2	7	7636.85818	7636.85237	0.00581
6	3	4	5	5	3	3	4	7637.12399	7637.11807	0.00592
6	3	3	5	5	3	2	4	7637.63134	7637.62497	0.00637
6	2	4	7	5	2	3	6	7660.88830	7660.88816	0.00014
6	2	4	8	5	2	3	7	7662.51902	7662.51722	0.00180
6	2	4	5	5	2	3	5	7670.95969	7670.96430	−0.00461
6	2	4	6	5	2	3	6	7663.27485	7663.27093	0.00392
3	2	2	2	4	1	3	3	7766.91669	7766.91651	0.00019
3	2	2	5	4	1	3	6	7771.22967	7771.22820	0.00147
3	2	2	3	4	1	3	4	7779.28891	7779.28643	0.00248
3	2	2	4	4	1	3	5	7783.68056	7783.68644	−0.00588
6	1	5	7	5	1	4	7	7845.76126	7845.76511	−0.00384
6	1	5	6	5	1	4	6	7862.07162	7862.07874	−0.00712
6	1	5	8	5	1	4	7	7859.25735	7859.26445	−0.00710
6	1	5	6	5	1	4	5	7858.37125	7858.36627	0.00499
6	1	5	5	5	1	4	4	7858.75759	7858.75302	0.00457
6	1	5	7	5	1	4	6	7858.87402	7858.87364	0.00038
6	1	5	5	5	1	4	5	7871.87118	7871.87164	−0.00046
3	1	3	4	2	0	2	4	7984.85225	7984.85816	−0.00591
3	1	3	2	2	0	2	1	7989.38155	7989.37697	0.00458
3	1	3	3	2	0	2	2	7993.39039	7993.38296	0.00743
3	1	3	5	2	0	2	4	7994.69771	7994.69027	0.00745
3	1	3	4	2	0	2	3	7998.77340	7998.77124	0.00216

Table S7: Assignment in Pickett notation of experimentally determined transitions of $^{13}C_1$-4-ClBzA

J'	K_a'	K_c'	F'	J''	K_a''	K_c''	F''	ν_{obs} / MHz	ν_{calc} / MHz	$\nu_{obs - calc}$ / MHz
2	1	2	4	1	1	1	3	2495.49358	2495.49439	−0.00082
2	0	2	2	1	0	1	1	2553.92750	2553.93321	−0.00571
2	0	2	3	1	0	1	3	2555.40801	2555.40897	−0.00095
2	0	2	4	1	0	1	3	2573.06211	2573.06097	0.00115
2	1	1	4	1	1	0	3	2658.48562	2658.48458	0.00104
3	1	3	4	2	1	2	3	3733.57541	3733.57472	0.00069
3	1	3	3	2	1	2	2	3733.63342	3733.62940	0.00402
3	1	3	5	2	1	2	4	3738.02297	3738.02401	−0.00104
3	0	3	4	2	0	2	4	3837.69104	3837.68683	0.00421
3	0	3	3	2	0	2	2	3850.91847	3850.92198	−0.00351
3	0	3	5	2	0	2	4	3855.32337	3855.32443	−0.00106
3	1	2	3	2	1	1	2	3977.59056	3977.59419	−0.00363
3	1	2	4	2	1	1	3	3977.91631	3977.91992	−0.00361
3	1	2	2	2	1	1	1	3982.00465	3981.99823	0.00643
3	1	2	5	2	1	1	4	3982.35510	3982.35517	−0.00007
1	1	0	2	1	0	1	1	4436.44714	4436.43360	0.01354
1	1	0	3	1	0	1	3	4442.54593	4442.54794	−0.00201
1	1	0	1	1	0	1	2	4453.71913	4453.70469	0.01444
1	1	0	3	1	0	1	2	4460.19819	4460.20072	−0.00253
1	1	0	2	1	0	1	2	4468.27898	4468.27745	0.00153
2	1	1	4	2	0	2	4	4527.97747	4527.97155	0.00593
7	2	5	9	8	1	8	10	4571.07591	4571.07893	−0.00303
3	1	2	2	3	0	3	2	4653.34223	4653.35165	−0.00941
3	1	2	5	3	0	3	5	4655.00702	4655.00229	0.00473
3	1	2	3	3	0	3	3	4656.91765	4656.91868	−0.00104
3	1	2	4	3	0	3	4	4658.66487	4658.67153	−0.00666
5	2	4	7	6	1	5	8	4659.23928	4659.24335	−0.00407
3	1	2	3	3	0	3	4	4665.20910	4665.21715	−0.00805
4	1	3	3	4	0	4	3	4826.74279	4826.74471	−0.00191
4	1	3	6	4	0	4	6	4827.39380	4827.39209	0.00172
4	1	3	5	4	0	4	5	4829.24500	4829.24020	0.00480
4	1	4	4	3	1	3	3	4978.66103	4978.66110	−0.00007
4	1	4	5	3	1	3	4	4979.49617	4979.49498	0.00119
4	1	4	3	3	1	3	2	4980.41036	4980.41078	−0.00041
4	1	4	6	3	1	3	5	4981.26801	4981.27062	−0.00261
5	1	4	7	5	0	5	6	5066.30107	5066.29946	0.00161
4	0	4	3	3	0	3	2	5132.54060	5132.54541	−0.00481
4	3	1	4	3	3	0	4	5134.60014	5134.62807	−0.02792
4	3	2	5	3	3	1	4	5136.80416	5136.79092	0.01324
4	3	1	5	3	3	0	4	5136.87058	5136.84933	0.02125

Table S7: Assignment in Pickett notation of experimentally determined transitions of $^{13}C_1$-4-ClBzA

J'	K_a'	K_c'	F'	J''	K_a''	K_c''	F''	ν_{obs} / MHz	ν_{calc} / MHz	$\nu_{obs - calc}$ / MHz
4	1	3	4	3	1	2	3	5304.19794	5304.19928	−0.00134
4	1	3	5	3	1	2	4	5305.19747	5305.19667	0.00080
4	1	3	3	3	1	2	2	5305.94088	5305.93848	0.00240
4	1	3	6	3	1	2	5	5306.97585	5306.97429	0.00156
6	1	5	5	6	0	6	5	5323.08014	5323.08229	−0.00215
6	1	5	8	6	0	6	8	5323.19188	5323.17340	0.01848
6	1	5	6	6	0	6	6	5323.46461	5323.46657	−0.00196
6	1	5	7	6	0	6	7	5323.56777	5323.56684	0.00094
7	0	7	9	6	1	6	8	5328.59676	5328.60351	−0.00675
1	1	1	3	0	0	0	2	5650.72447	5650.72671	−0.00224
7	1	6	8	7	0	7	8	5655.39768	5655.40303	−0.00535
1	1	1	2	0	0	0	2	5660.30918	5660.31175	−0.00257
5	1	5	5	4	1	4	4	6222.28610	6222.27876	0.00735
5	1	5	6	4	1	4	5	6223.00905	6223.01314	−0.00409
5	1	5	4	4	1	4	3	6223.14663	6223.14951	−0.00288
5	1	5	7	4	1	4	6	6223.89490	6223.89502	−0.00012
5	0	5	5	4	0	4	4	6408.30227	6408.32235	−0.02007
5	0	5	7	4	0	4	6	6409.52294	6409.51489	0.00805
5	2	4	5	4	2	3	4	6427.68624	6427.64475	0.04149
5	2	3	7	4	2	2	6	6453.21559	6453.21668	−0.00108
5	1	4	5	4	1	3	4	6629.13885	6629.14399	−0.00514
5	1	4	6	4	1	3	5	6629.97140	6629.97910	−0.00770
5	1	4	7	4	1	3	6	6630.85448	6630.86012	−0.00564
2	1	2	3	1	0	1	3	6848.28732	6848.29531	−0.00799
2	1	2	4	1	0	1	3	6856.31963	6856.31863	0.00100
6	0	6	6	5	0	5	5	7678.19269	7678.21639	−0.02370
6	0	6	8	5	0	5	7	7679.00695	7678.99047	0.01648

Table S8: Assignment in Pickett notation of experimentally determined transitions of $^{13}C_2$-4-ClBzA

J'	K_a'	K_c'	F'	J''	K_a''	K_c''	F''	ν_{obs} / MHz	ν_{calc} / MHz	$\nu_{obs - calc}$ / MHz
1	1	0	2	1	0	1	1	4435.93663	4435.92927	0.00735
1	1	0	3	1	0	1	3	4442.04105	4442.04377	−0.00271
1	1	0	2	1	0	1	3	4450.10364	4450.11432	−0.01068
1	1	0	1	1	0	1	2	4453.20061	4453.19559	0.00503
1	1	0	3	1	0	1	2	4459.69576	4459.68862	0.00714
2	1	1	4	2	0	2	4	4528.81145	4528.80678	0.00468
2	1	1	3	2	0	2	3	4536.92902	4536.92010	0.00892

Table S8: Assignment in Pickett notation of experimentally determined transitions of $^{13}C_2$-4-ClBzA

J'	K_a'	K_c'	F'	J''	K_a''	K_c''	F''	ν_{obs} / MHz	ν_{calc} / MHz	$\nu_{obs - calc}$ / MHz
3	1	2	2	3	0	3	2	4656.27531	4656.26871	0.00660
3	1	2	5	3	0	3	5	4657.92747	4657.93052	−0.00305
3	1	2	4	3	0	3	4	4661.58754	4661.58715	0.00039
4	1	3	3	4	0	4	3	4832.57636	4832.58278	−0.00641
4	1	3	6	4	0	4	6	4833.21784	4833.22055	−0.00271
4	1	3	4	4	0	4	4	4834.40029	4834.40345	−0.00316
4	1	3	5	4	0	4	5	4835.07085	4835.06959	0.00126
4	1	4	4	3	1	3	3	5019.41284	5019.41362	−0.00078
4	1	4	5	3	1	3	4	5020.25006	5020.24676	0.00329
4	1	4	3	3	1	3	2	5021.16062	5021.16235	−0.00173
4	1	4	6	3	1	3	5	5022.02286	5022.02123	0.00162
4	0	4	4	3	0	3	3	5175.61305	5175.61051	0.00254
4	0	4	5	3	0	3	4	5177.67242	5177.68707	−0.01465
6	1	5	5	6	0	6	5	5337.62937	5337.62639	0.00297
6	1	5	8	6	0	6	8	5337.70830	5337.71536	−0.00705
6	1	5	6	6	0	6	6	5338.00904	5338.00537	0.00367
6	1	5	7	6	0	6	7	5338.10746	5338.10498	0.00248
4	1	3	6	3	1	2	5	5352.93843	5352.94292	−0.00449
1	1	1	1	0	0	0	2	5652.10215	5652.09636	0.00579
1	1	1	3	0	0	0	2	5659.77521	5659.77475	0.00047
1	1	1	2	0	0	0	2	5669.35985	5669.35615	0.00370
5	1	5	5	4	1	4	4	6273.15088	6273.15308	−0.00220
5	1	5	6	4	1	4	5	6273.89241	6273.88678	0.00563
5	1	5	4	4	1	4	3	6274.02314	6274.02321	−0.00007
5	1	5	7	4	1	4	6	6274.76947	6274.76795	0.00152
5	2	3	6	6	1	6	7	6360.17444	6360.18318	−0.00874
5	0	5	5	4	0	4	4	6461.87401	6461.88811	−0.01410
5	0	5	7	4	0	4	6	6463.08548	6463.07786	0.00762
5	2	4	5	4	2	3	4	6481.86017	6481.85877	0.00140
5	1	4	5	4	1	3	4	6686.53600	6686.53860	−0.00260
5	1	4	6	4	1	3	5	6687.37600	6687.37400	0.00199
5	1	4	7	4	1	3	6	6688.25499	6688.25372	0.00126
2	1	2	3	1	0	1	2	6884.54613	6884.54463	0.00150
6	1	6	5	5	1	5	4	7525.73157	7525.75789	−0.02632
6	1	6	7	5	1	5	6	7525.83265	7525.84150	−0.00885
6	1	6	8	5	1	5	7	7526.33284	7526.33888	−0.00605
6	0	6	5	5	0	5	4	7742.07100	7742.05356	0.01745
6	0	6	8	5	0	5	7	7742.86683	7742.87195	−0.00513
6	2	5	7	5	2	4	6	7776.87006	7776.85300	0.01706
6	2	4	5	5	2	3	4	7819.41218	7819.40221	0.00997

Table S9: Assignment in Pickett notation of experimentally determined transitions of $^{13}C_3$-4-ClBzA

J'	K'_a	K'_c	F'	J''	K''_a	K''_c	F''	ν_{obs} / MHz	ν_{calc} / MHz	$\nu_{obs - calc}$ / MHz
2	1	2	3	1	1	1	2	2500.40956	2500.41105	−0.00150
2	0	2	4	1	0	1	3	2597.84069	2597.83996	0.00073
3	1	3	5	2	1	2	4	3771.74354	3771.74121	0.00233
3	0	3	5	2	0	2	4	3892.30174	3892.29314	0.00860
3	1	2	3	2	1	1	2	4018.36020	4018.36975	−0.00955
3	1	2	4	2	1	1	3	4018.68917	4018.68857	0.00060
3	1	2	5	2	1	1	4	4023.13426	4023.12217	0.01209
1	1	0	2	1	0	1	1	4384.45538	4384.45622	−0.00084
1	1	0	3	1	0	1	3	4390.56075	4390.56193	−0.00117
1	1	0	3	1	0	1	2	4408.19262	4408.19767	−0.00505
2	1	1	3	2	0	2	4	4468.88386	4468.89006	−0.00620
2	1	1	1	2	0	2	1	4472.72735	4472.73856	−0.01122
2	1	1	3	2	0	2	2	4473.81144	4473.82226	−0.01082
2	1	1	4	2	0	2	4	4478.41093	4478.41137	−0.00045
2	1	1	2	2	0	2	2	4480.68682	4480.68320	0.00362
2	1	1	3	2	0	2	3	4486.52705	4486.52393	0.00313
2	1	1	1	2	0	2	2	4490.29303	4490.30522	−0.01219
3	1	2	5	3	0	3	5	4609.23467	4609.24040	−0.00573
3	1	2	3	3	0	3	3	4611.15474	4611.15742	−0.00268
3	1	2	4	3	0	3	4	4612.90664	4612.90323	0.00341
4	1	3	3	4	0	4	3	4786.32665	4786.32218	0.00447
4	1	3	6	4	0	4	6	4786.96625	4786.96421	0.00204
4	1	3	5	4	0	4	5	4788.81643	4788.81347	0.00296
5	1	4	4	5	0	5	4	5015.15283	5015.15881	−0.00598
5	1	4	7	5	0	5	7	5015.42163	5015.41823	0.00339
5	1	4	5	5	0	5	5	5016.06966	5016.07593	−0.00627
5	1	4	6	5	0	5	6	5016.34636	5016.35175	−0.00539
4	1	4	4	3	1	3	3	5023.53461	5023.53016	0.00444
4	1	4	5	3	1	3	4	5024.37054	5024.36355	0.00698
4	1	4	3	3	1	3	2	5025.28004	5025.27788	0.00216
4	1	4	6	3	1	3	5	5026.14674	5026.13695	0.00978
4	1	4	4	3	1	3	4	5029.39124	5029.39170	−0.00046
4	0	4	5	3	0	3	5	5165.92036	5165.92477	−0.00441
4	0	4	4	3	0	3	3	5181.46956	5181.46361	0.00595
4	0	4	6	3	0	3	5	5183.51112	5183.50235	0.00877
4	2	3	6	3	2	2	5	5196.69658	5196.68742	0.00915
6	1	5	5	6	0	6	5	5298.93273	5298.91709	0.01563
6	1	5	8	6	0	6	8	5299.01158	5299.00708	0.00449
6	1	5	6	6	0	6	6	5299.32298	5299.29807	0.02491
6	1	5	7	6	0	6	7	5299.40193	5299.39781	0.00411

Table S9: Assignment in Pickett notation of experimentally determined transitions of $^{13}C_3$-4-ClBzA

J'	K'_a	K'_c	F'	J''	K''_a	K''_c	F''	ν_{obs} / MHz	ν_{calc} / MHz	$\nu_{obs - calc}$ / MHz
7	0	7	6	6	1	6	6	5500.04789	5500.07915	−0.03126
1	1	1	1	0	0	0	2	5601.14675	5601.15153	−0.00477
1	1	1	3	0	0	0	2	5608.81496	5608.82099	−0.00603
1	1	1	2	0	0	0	2	5618.39501	5618.39353	0.00147
7	1	6	9	7	0	7	8	5660.12391	5660.13054	−0.00662
5	1	5	5	4	1	4	4	6278.22564	6278.22065	0.00499
5	1	5	4	4	1	4	3	6279.09656	6279.09016	0.00639
5	1	5	7	4	1	4	6	6279.83371	6279.83500	−0.00129
5	1	4	5	4	1	3	4	6696.81277	6696.80588	0.00689
5	1	4	4	4	1	3	3	6697.65365	6697.67357	−0.01992
5	1	4	7	4	1	3	6	6698.52828	6698.51991	0.00837
2	1	2	4	1	0	1	3	6824.48939	6824.49427	−0.00488
2	1	2	3	1	0	1	2	6834.11428	6834.11099	0.00329
8	0	8	7	7	1	7	6	6991.96358	6991.96847	−0.00489
6	1	6	7	5	1	5	6	7531.81467	7531.81186	0.00281
6	1	6	8	5	1	5	7	7532.31649	7532.30877	0.00772
6	0	6	6	5	0	5	5	7750.00965	7750.01943	−0.00978
6	0	6	8	5	0	5	7	7750.80321	7750.79080	0.01241
3	1	3	4	2	0	2	4	7985.91313	7985.93219	−0.01906
3	1	3	5	2	0	2	4	7998.39932	7998.39551	0.00381

Table S10: Assignment in Pickett notation of experimentally determined transitions of $^{13}C_4$-4-ClBzA

J'	K'_a	K'_c	F'	J''	K''_a	K''_c	F''	ν_{obs} / MHz	ν_{calc} / MHz	$\nu_{obs - calc}$ / MHz
2	1	2	3	1	1	1	2	2501.47351	2501.47257	0.00094
5	0	5	4	4	1	4	3	2539.51305	2539.49869	0.01436
5	0	5	7	4	1	4	6	2539.57484	2539.57084	0.00400
2	0	2	3	1	0	1	2	2599.21662	2599.21750	−0.00087
2	1	1	4	1	1	0	3	2687.40015	2687.39101	0.00913
3	1	3	4	2	1	2	3	3768.90213	3768.89863	0.00350
3	1	3	5	2	1	2	4	3773.34670	3773.34737	−0.00067
3	2	2	5	2	2	1	4	3903.42929	3903.43463	−0.00535
3	2	1	5	2	2	0	4	3908.32998	3908.32882	0.00116
6	0	6	5	5	1	5	4	4011.45123	4011.46481	−0.01358
6	0	6	8	5	1	5	7	4011.61578	4011.61049	0.00529
6	0	6	6	5	1	5	6	4013.41934	4013.40222	0.01713
3	1	2	3	2	1	1	2	4020.91221	4020.91320	−0.00099
3	1	2	4	2	1	1	3	4021.22736	4021.22695	0.00041

Table S10: Assignment in Pickett notation of experimentally determined transitions of $^{13}C_4$-4-ClBzA

J'	K_a'	K_c'	F'	J''	K_a''	K_c''	F''	ν_{obs} / MHz	ν_{calc} / MHz	$\nu_{obs - calc}$ / MHz
3	1	2	5	2	1	1	4	4025.66678	4025.66738	−0.00060
1	1	0	2	1	0	1	1	4370.97185	4370.97060	0.00124
1	1	0	3	1	0	1	3	4377.08312	4377.08215	0.00097
2	1	1	3	2	0	2	4	4455.73505	4455.72872	0.00633
2	1	1	1	2	0	2	1	4459.59460	4459.57935	0.01525
2	1	1	4	2	0	2	4	4465.25931	4465.25792	0.00139
2	1	1	2	2	0	2	2	4467.53664	4467.52985	0.00679
2	1	1	3	2	0	2	3	4473.37423	4473.38008	−0.00585
2	1	1	2	2	0	2	3	4480.24686	4480.24476	0.00209
2	1	1	4	2	0	2	3	4482.91514	4482.90928	0.00586
3	1	2	5	3	0	3	5	4596.60048	4596.60279	−0.00231
3	1	2	4	3	0	3	4	4600.27081	4600.26704	0.00377
4	1	3	6	4	0	4	6	4775.03937	4775.05117	−0.01180
4	1	3	4	4	0	4	4	4776.26212	4776.23676	0.02536
4	1	3	5	4	0	4	5	4776.89891	4776.90462	−0.00571
5	1	4	7	5	0	5	7	5004.48456	5004.48156	0.00300
5	1	4	5	5	0	5	5	5005.13413	5005.14107	−0.00693
5	1	4	6	5	0	5	6	5005.41720	5005.41695	0.00025
4	1	4	4	3	1	3	3	5025.66201	5025.65305	0.00896
4	1	4	5	3	1	3	4	5026.48554	5026.48735	−0.00181
4	1	4	3	3	1	3	2	5027.40185	5027.40245	−0.00060
4	1	4	6	3	1	3	5	5028.26230	5028.26248	−0.00019
4	0	4	4	3	0	3	3	5184.10596	5184.10601	−0.00005
4	0	4	5	3	0	3	4	5186.16533	5186.18461	−0.01928
4	2	3	5	3	2	2	4	5192.36564	5192.36132	0.00432
4	2	3	6	3	2	2	5	5199.45641	5199.45379	0.00262
4	2	2	5	3	2	1	4	5204.53417	5204.54276	−0.00859
4	1	3	5	3	1	2	4	5362.80357	5362.82220	−0.01863
4	1	3	3	3	1	2	2	5363.56439	5363.56533	−0.00094
4	1	3	6	3	1	2	5	5364.57868	5364.59637	−0.01769
1	1	1	1	0	0	0	2	5588.04617	5588.05003	−0.00386
1	1	1	2	0	0	0	2	5605.30785	5605.30805	−0.00019
7	1	6	9	7	0	7	9	5634.61941	5634.63590	−0.01649
7	1	6	8	7	0	7	8	5634.68039	5634.66952	0.01087
5	1	5	5	4	1	4	4	6280.85271	6280.85256	0.00014
5	1	5	6	4	1	4	5	6281.58631	6281.58712	−0.00081
5	1	5	4	4	1	4	3	6281.71727	6281.72287	−0.00560
5	1	5	7	4	1	4	6	6282.46317	6282.46845	−0.00529
5	0	5	5	4	0	4	4	6472.07274	6472.08829	−0.01555
5	0	5	7	4	0	4	6	6473.28483	6473.27773	0.00710

Table S10: Assignment in Pickett notation of experimentally determined transitions of $^{13}C_4$-4-ClBzA

J'	K'_a	K'_c	F'	J''	K''_a	K''_c	F''	ν_{obs} / MHz	ν_{calc} / MHz	$\nu_{obs - calc}$ / MHz
5	1	4	5	4	1	3	4	6700.98843	6700.99260	−0.00416
5	1	4	6	4	1	3	5	6701.82825	6701.82821	0.00005
5	1	4	7	4	1	3	6	6702.70937	6702.70811	0.00126
2	1	2	2	1	0	1	1	6795.37968	6795.37639	0.00328
2	1	2	1	1	0	1	1	6803.50955	6803.51784	−0.00829
2	1	2	3	1	0	1	3	6803.75578	6803.75435	0.00143
2	1	2	4	1	0	1	3	6811.78247	6811.78276	−0.00029
2	1	2	3	1	0	1	2	6821.41139	6821.40797	0.00342
6	1	6	6	5	1	5	5	7534.36805	7534.36272	0.00532
6	1	6	5	5	1	5	4	7534.86277	7534.85400	0.00877
6	1	6	7	5	1	5	6	7534.93344	7534.93884	−0.00540
6	1	6	8	5	1	5	7	7535.43663	7535.43616	0.00046
6	0	6	5	5	0	5	4	7753.71787	7753.68898	0.02889
6	0	6	8	5	0	5	7	7754.50696	7754.50811	−0.00114
6	2	5	5	5	2	4	4	7792.18102	7792.18789	−0.00688
3	1	3	4	2	0	2	3	7991.08609	7991.08911	−0.00302

Table S11: Assignment in Pickett notation of experimentally determined transitions of $^{13}C_5$-4-ClBzA

J'	K'_a	K'_c	F'	J''	K''_a	K''_c	F''	ν_{obs} / MHz	ν_{calc} / MHz	$\nu_{obs - calc}$ / MHz
2	0	2	4	1	0	1	3	2596.5955	2596.6	−0.00444
2	1	1	4	1	1	0	3	2683.4425	2683.4402	0.00226
3	1	3	5	2	1	2	4	3771.2813	3771.2971	−0.01579
3	0	3	3	2	0	2	2	3886.123	3886.1379	−0.01485
3	0	3	5	2	0	2	4	3890.5339	3890.538	−0.00412
3	0	3	3	2	0	2	3	3898.8529	3898.847	0.00591
3	2	1	3	2	2	0	2	3899.094	3899.0795	0.01452
3	2	2	5	2	2	1	4	3899.4182	3899.4277	−0.00946
6	0	6	8	5	1	5	7	3929.552	3929.5611	−0.0091
3	1	2	4	2	1	1	3	4015.3274	4015.3342	−0.00677
3	1	2	5	2	1	1	4	4019.7696	4019.7686	0.00097
1	1	0	3	1	0	1	3	4444.3238	4444.3305	−0.00671
1	1	0	1	1	0	1	2	4455.4843	4455.4842	0.00002
1	1	0	3	1	0	1	2	4461.9749	4461.9764	−0.00148
1	1	0	2	1	0	1	2	4470.049	4470.0483	0.00068
2	1	1	4	2	0	2	4	4531.1824	4531.1708	0.01158
2	1	1	3	2	0	2	3	4539.2859	4539.2841	0.00182
2	1	1	1	2	0	2	2	4543.0835	4543.0743	0.00927

Table S11: Assignment in Pickett notation of experimentally determined transitions of $^{13}C_5$-4-ClBzA

J'	K'_a	K'_c	F'	J''	K''_a	K''_c	F''	ν_{obs} / MHz	ν_{calc} / MHz	$\nu_{obs - calc}$ / MHz
2	1	1	2	2	0	2	3	4546.1534	4546.1531	0.00031
2	1	1	4	2	0	2	3	4548.8163	4548.8153	0.00098
3	1	2	4	3	0	3	3	4655.7739	4655.7713	0.00261
3	1	2	2	3	0	3	2	4658.7636	4658.7501	0.0135
3	1	2	5	3	0	3	5	4660.403	4660.4014	0.00166
3	1	2	3	3	0	3	3	4662.3111	4662.3167	−0.00562
4	1	3	3	4	0	4	3	4835.2111	4835.2087	0.00236
4	1	3	6	4	0	4	6	4835.8523	4835.8529	−0.00058
4	1	3	4	4	0	4	4	4837.0404	4837.0335	0.00691
4	1	3	5	4	0	4	5	4837.7068	4837.6987	0.00815
4	1	4	4	3	1	3	3	5022.9821	5022.984	−0.00194
4	1	4	5	3	1	3	4	5023.8141	5023.8174	−0.00324
4	1	4	3	3	1	3	2	5024.7254	5024.7329	−0.0075
4	1	4	6	3	1	3	5	5025.5908	5025.592	−0.00122
4	1	4	4	3	1	3	4	5028.8599	5028.8472	0.01267
5	1	4	4	5	0	5	4	5060.9751	5060.9757	−0.00052
5	1	4	6	5	0	5	6	5062.1749	5062.1668	0.00816
4	0	4	4	3	0	3	3	5179.3134	5179.3187	−0.00535
4	0	4	6	3	0	3	5	5181.3671	5181.3584	0.0087
4	2	3	5	3	2	2	4	5187.0721	5187.0657	0.00638
4	2	3	6	3	2	2	5	5194.1677	5194.1549	0.01274
4	2	3	3	3	2	2	2	5196.5807	5196.5924	−0.0117
6	1	5	5	6	0	6	5	5340.7237	5340.7231	0.00053
6	1	5	8	6	0	6	8	5340.8221	5340.8131	0.00903
6	1	5	6	6	0	6	6	5341.1046	5341.1031	0.00151
6	1	5	7	6	0	6	7	5341.2085	5341.2024	0.00613
4	1	3	5	3	1	2	4	5355.0473	5355.0342	0.01302
4	1	3	6	3	1	2	5	5356.8077	5356.81	−0.00229
7	0	7	8	6	1	6	7	5418.4866	5418.4895	−0.00288
7	0	7	9	6	1	6	8	5420.3212	5420.3224	−0.00125
1	1	1	1	0	0	0	2	5655.2407	5655.2401	0.00063
1	1	1	3	0	0	0	2	5662.9158	5662.9168	−0.00097
1	1	1	2	0	0	0	2	5672.4994	5672.4994	0.00003
7	1	6	9	7	0	7	9	5679.3929	5679.3956	−0.0027
8	1	7	8	8	0	8	9	6085.1189	6085.1396	−0.02067
4	2	3	5	5	1	4	6	6125.8124	6125.8297	−0.01729
5	1	5	5	4	1	4	4	6277.6084	6277.6135	−0.00501
5	1	5	6	4	1	4	5	6278.3463	6278.3474	−0.00108
5	1	5	4	4	1	4	3	6278.4738	6278.4837	−0.00993
5	1	5	7	4	1	4	6	6279.226	6279.2287	−0.00268

Table S11: Assignment in Pickett notation of experimentally determined transitions of $^{13}C_5$-4-ClBzA

J'	K'_a	K'_c	F'	J''	K''_a	K''_c	F''	ν_{obs} / MHz	ν_{calc} / MHz	$\nu_{obs - calc}$ / MHz
5	0	5	5	4	0	4	4	6466.4864	6466.508	−0.02155
5	0	5	7	4	0	4	6	6467.7092	6467.6988	0.01032
5	2	3	7	4	2	2	6	6512.8002	6512.8098	−0.00965
5	1	4	5	4	1	3	4	6691.3603	6691.3658	−0.00555
5	1	4	6	4	1	3	5	6692.1969	6692.201	−0.00411
5	1	4	7	4	1	3	6	6693.0767	6693.0812	−0.00456
2	1	2	4	1	0	1	3	6878.9099	6878.9177	−0.0078
2	1	2	3	1	0	1	2	6888.5437	6888.5444	−0.00066
6	1	6	6	5	1	5	5	7530.6147	7530.6208	−0.00609
6	1	6	8	5	1	5	7	7531.6987	7531.6879	0.01075
6	0	6	5	5	0	5	4	7747.6066	7747.5819	0.02471
6	0	6	8	5	0	5	7	7748.4056	7748.4013	0.0043
6	3	3	8	5	3	2	7	7797.3013	7797.2857	0.01553

Table S12: Assignment in Pickett notation of experimentally determined transitions of $^{13}C_6$-4-ClBzA

J'	K'_a	K'_c	F'	J''	K''_a	K''_c	F''	ν_{obs} / MHz	ν_{calc} / MHz	$\nu_{obs - calc}$ / MHz
5	0	5	6	4	1	4	5	2545.64131	2545.63721	0.00410
2	0	2	3	1	0	1	2	2597.94925	2597.95234	−0.00309
3	1	3	5	2	1	2	4	3771.28144	3771.26404	0.01740
3	0	3	5	2	0	2	4	3892.40216	3892.40844	−0.00627
3	2	2	3	2	2	1	2	3896.55240	3896.54327	0.00912
3	0	3	3	2	0	2	3	3900.71505	3900.71178	0.00327
6	0	6	6	5	1	5	5	4017.93895	4017.95157	−0.01262
6	0	6	7	5	1	5	6	4018.07722	4018.08213	−0.00492
3	1	2	3	2	1	1	2	4019.20992	4019.22124	−0.01132
3	1	2	4	2	1	1	3	4019.51676	4019.53320	−0.01644
1	1	0	1	1	0	1	1	4344.40444	4344.38033	0.02411
1	1	0	2	1	0	1	1	4358.95174	4358.95030	0.00144
1	1	0	3	1	0	1	3	4365.05289	4365.05200	0.00090
1	1	0	2	1	0	1	3	4373.12338	4373.12629	−0.00291
1	1	0	1	1	0	1	2	4376.17193	4376.19021	−0.01829
2	1	1	4	2	0	2	4	4453.35645	4453.35961	−0.00316
2	1	1	2	2	0	2	2	4455.62427	4455.62960	−0.00533
2	1	1	3	2	0	2	3	4461.47074	4461.47574	−0.00500
2	1	1	1	2	0	2	2	4465.25931	4465.25039	0.00892
2	1	1	4	2	0	2	3	4470.98906	4470.99118	−0.00212
3	1	2	5	3	0	3	5	4584.92473	4584.92015	0.00459

Table S12: Assignment in Pickett notation of experimentally determined transitions of $^{13}C_6$-4-ClBzA

J'	K'_a	K'_c	F'	J''	K''_a	K''_c	F''	ν_{obs} / MHz	ν_{calc} / MHz	$\nu_{obs - calc}$ / MHz
3	1	2	4	3	0	3	4	4588.58612	4588.58286	0.00326
4	1	3	3	4	0	4	3	4763.03760	4763.04086	−0.00326
4	1	3	6	4	0	4	6	4763.67874	4763.68088	−0.00214
4	1	3	4	4	0	4	4	4764.86475	4764.86725	−0.00251
4	1	3	5	4	0	4	5	4765.53402	4765.53547	−0.00145
5	1	4	6	5	0	5	5	4989.51363	4989.49496	0.01868
5	1	4	4	5	0	5	4	4993.27749	4993.28188	−0.00439
5	1	4	7	5	0	5	7	4993.54118	4993.54103	0.00015
5	1	4	5	5	0	5	5	4994.20003	4994.20201	−0.00198
5	1	4	6	5	0	5	6	4994.48249	4994.47837	0.00412
4	1	4	4	3	1	3	3	5022.87235	5022.87133	0.00102
4	1	4	5	3	1	3	4	5023.69526	5023.70505	−0.00979
4	1	4	6	3	1	3	5	5025.48611	5025.47816	0.00794
4	0	4	4	3	0	3	3	5181.52272	5181.52353	−0.00082
4	0	4	5	3	0	3	4	5183.59134	5183.59946	−0.00812
4	2	3	5	3	2	2	4	5189.83321	5189.84816	−0.01494
4	2	3	4	3	2	2	3	5192.36554	5192.35341	0.01213
4	2	3	6	3	2	2	5	5196.94139	5196.93269	0.00869
6	1	5	5	6	0	6	5	5278.88732	5278.88027	0.00705
6	1	5	8	6	0	6	8	5278.97192	5278.97047	0.00146
6	1	5	6	6	0	6	6	5279.26436	5279.26399	0.00037
6	1	5	7	6	0	6	7	5279.36269	5279.36466	−0.00197
4	1	3	5	3	1	2	4	5360.56049	5360.55207	0.00843
7	0	7	9	6	1	6	8	5513.21619	5513.22732	−0.01113
1	1	1	3	0	0	0	2	5582.93244	5582.93835	−0.00591
1	1	1	2	0	0	0	2	5592.50893	5592.50524	0.00369
5	1	5	5	4	1	4	4	6277.36660	6277.36181	0.00479
5	1	5	6	4	1	4	5	6278.09829	6278.09574	0.00255
5	1	5	4	4	1	4	3	6278.22564	6278.23113	−0.00549
5	1	5	7	4	1	4	6	6278.95505	6278.97607	−0.02102
5	0	5	4	4	0	4	3	6468.78714	6468.76815	0.01899
5	0	5	7	4	0	4	6	6470.01258	6469.99445	0.01813
5	0	5	6	4	0	4	5	6470.04798	6470.03284	0.01514
5	1	4	5	4	1	3	4	6698.14402	6698.14123	0.00279
5	1	4	4	4	1	3	3	6698.98369	6699.00917	−0.02548
5	1	4	7	4	1	3	6	6699.85549	6699.85460	0.00089
2	1	2	2	1	0	1	1	6781.84362	6781.84773	−0.00411
2	1	2	4	1	0	1	3	6798.22224	6798.23681	−0.01457
2	1	2	3	1	0	1	2	6807.85184	6807.84814	0.00371
6	1	6	6	5	1	5	5	7530.15719	7530.15508	0.00211

Table S12: Assignment in Pickett notation of experimentally determined transitions of $^{13}C_6$-4-ClBzA

J'	K_a'	K_c'	F'	J''	K_a''	K_c''	F''	ν_{obs} / MHz	ν_{calc} / MHz	$\nu_{obs - calc}$ / MHz
6	1	6	7	5	1	5	6	7530.73529	7530.73093	0.00436
6	0	6	5	5	0	5	4	7749.69510	7749.67425	0.02085
6	0	6	8	5	0	5	7	7750.50204	7750.49240	0.00964
3	1	3	4	2	0	2	4	7959.05091	7959.08455	−0.03364
3	1	3	3	2	0	2	2	7969.87888	7969.87795	0.00094
3	1	3	5	2	0	2	4	7971.54951	7971.55084	−0.00133
3	1	3	4	2	0	2	3	7976.72254	7976.71611	0.00643

Table S13: Assignment in Pickett notation of experimentally determined transitions of $^{13}C_7$-4-ClBzA

J'	K_a'	K_c'	F'	J''	K_a''	K_c''	F''	ν_{obs} / MHz	ν_{calc} / MHz	$\nu_{obs - calc}$ / MHz
2	1	2	3	1	1	1	2	2499.62092	2499.62285	−0.00194
5	0	5	7	4	1	4	6	2562.95108	2562.94671	0.00437
2	0	2	4	1	0	1	3	2597.69132	2597.69517	−0.00385
2	1	1	3	1	1	0	2	2668.62304	2668.62841	−0.00537
6	2	5	8	7	1	6	9	2694.38041	2694.39212	−0.01171
3	1	3	5	2	1	2	4	3770.56086	3770.56085	0.00001
3	0	3	3	2	0	2	2	3887.59831	3887.59698	0.00133
3	0	3	5	2	0	2	4	3891.99402	3891.99867	−0.00465
1	1	0	3	1	0	1	3	4351.29808	4351.29994	−0.00186
1	1	0	1	1	0	1	2	4362.45874	4362.45492	0.00381
1	1	0	3	1	0	1	2	4368.95416	4368.95245	0.00172
2	1	1	3	2	0	2	4	4430.30945	4430.31042	−0.00097
2	1	1	3	2	0	2	2	4435.24805	4435.24682	0.00123
2	1	1	3	2	0	2	3	4447.95699	4447.96059	−0.00359
2	1	1	2	2	0	2	3	4454.83397	4454.82836	0.00561
2	1	1	4	2	0	2	3	4457.50145	4457.49231	0.00914
3	1	2	4	3	0	3	3	4567.14492	4567.13351	0.01141
3	1	2	2	3	0	3	2	4570.11425	4570.10815	0.00610
3	1	2	5	3	0	3	5	4571.76545	4571.76605	−0.00060
3	1	2	3	3	0	3	3	4573.68414	4573.68408	0.00006
3	1	2	4	3	0	3	4	4575.43423	4575.42857	0.00567
4	1	3	3	4	0	4	3	4750.42569	4750.40999	0.01571
4	1	3	6	4	0	4	6	4751.06138	4751.04999	0.01139
4	1	3	4	4	0	4	4	4752.20966	4752.23258	−0.02292
4	1	3	5	4	0	4	5	4752.89876	4752.89905	−0.00029
5	1	4	4	5	0	5	4	4981.35994	4981.36077	−0.00083
5	1	4	6	5	0	5	6	4982.54857	4982.55006	−0.00149

Table S13: Assignment in Pickett notation of experimentally determined transitions of $^{13}C_7$-4-ClBzA

J'	K_a'	K_c'	F'	J''	K_a''	K_c''	F''	ν_{obs} / MHz	ν_{calc} / MHz	$\nu_{obs - calc}$ / MHz
4	1	4	5	3	1	3	4	5022.75042	5022.75249	−0.00207
4	1	4	6	3	1	3	5	5024.53255	5024.52742	0.00512
4	1	4	4	3	1	3	4	5027.79014	5027.78470	0.00544
4	0	4	3	3	0	3	2	5180.92354	5180.91834	0.00521
4	0	4	5	3	0	3	4	5182.98730	5183.00728	−0.01998
4	2	2	4	3	2	1	3	5204.20376	5204.21460	−0.01084
4	2	2	6	3	2	1	5	5208.84552	5208.82496	0.02056
6	1	5	5	6	0	6	5	5267.89202	5267.88915	0.00287
6	1	5	8	6	0	6	8	5267.97563	5267.97801	−0.00237
6	1	5	6	6	0	6	6	5268.26467	5268.26602	−0.00135
6	1	5	7	6	0	6	7	5268.36058	5268.36495	−0.00438
4	1	3	5	3	1	2	4	5360.46781	5360.47776	−0.00996
1	1	1	3	0	0	0	2	5568.84657	5568.84431	0.00225
1	1	1	2	0	0	0	2	5578.43021	5578.42738	0.00283
7	1	6	9	7	0	7	9	5615.17088	5615.19716	−0.02628
7	1	6	8	7	0	7	8	5615.24095	5615.22548	0.01547
5	1	5	5	4	1	4	4	6276.15843	6276.15306	0.00537
5	1	5	6	4	1	4	5	6276.88752	6276.88748	0.00004
5	1	5	4	4	1	4	3	6277.01187	6277.02318	−0.01132
5	1	5	7	4	1	4	6	6277.76537	6277.76864	−0.00326
5	0	5	5	4	0	4	4	6467.95661	6467.98435	−0.02774
5	0	5	7	4	0	4	6	6469.18340	6469.17340	0.00999
5	1	4	5	4	1	3	4	6698.01938	6698.02675	−0.00737
5	1	4	7	4	1	3	6	6699.74127	6699.74241	−0.00114
2	1	2	1	1	0	1	1	6775.53564	6775.53451	0.00113
2	1	2	3	1	0	1	3	6775.77909	6775.77536	0.00373
2	1	2	4	1	0	1	3	6783.80205	6783.80041	0.00164
2	1	2	3	1	0	1	2	6793.43417	6793.42787	0.00630
2	1	2	2	1	0	1	2	6799.23603	6799.23973	−0.00369
6	1	6	6	5	1	5	5	7528.67134	7528.67945	−0.00811
6	1	6	5	5	1	5	4	7529.16495	7529.17044	−0.00549
6	1	6	7	5	1	5	6	7529.25286	7529.25518	−0.00232
6	1	6	8	5	1	5	7	7529.74804	7529.75231	−0.00427
6	0	6	5	5	0	5	4	7748.61070	7748.57718	0.03352
6	0	6	8	5	0	5	7	7749.40865	7749.39657	0.01208
6	3	4	7	5	3	3	6	7796.00899	7796.01705	−0.00807
3	1	3	2	2	0	2	1	7949.95536	7949.93668	0.01868
3	1	3	3	2	0	2	2	7954.99713	7954.99522	0.00191
3	1	3	5	2	0	2	4	7956.66442	7956.66608	−0.00166
3	1	3	4	2	0	2	3	7961.84737	7961.84276	0.00461

Table S13: Assignment in Pickett notation of experimentally determined transitions of $^{13}C_7$-4-ClBzA

J'	K_a'	K_c'	F'	J''	K_a''	K_c''	F''	ν_{obs} / MHz	ν_{calc} / MHz	$\nu_{obs - calc}$ / MHz
3	1	3	3	2	0	2	3	7967.68683	7967.70899	−0.02216

6.2 3-ClBzA

Table S14: Assignment in Pickett notation of experimentally determined transitions of c-3-^{35}ClBzA

J'	K_a'	K_c'	F'	J''	K_a''	K_c''	F''	ν_{obs} / MHz	ν_{calc} / MHz	$\nu_{obs - calc}$ / MHz
2	2	0	4	1	1	1	3	8032.07614	8032.07550	0.00064
2	2	0	2	1	1	1	2	8033.72582	8033.72623	−0.00041
2	2	0	2	1	1	1	1	8035.58849	8035.58829	0.0002
2	2	0	3	1	1	1	3	8042.00598	8042.00640	−0.00042
6	0	6	6	5	1	5	5	8581.76617	8581.76635	−0.00018
6	0	6	5	5	1	5	4	8582.01052	8582.01054	−0.00002
6	0	6	7	5	1	5	6	8582.25597	8582.25661	−0.00064
6	0	6	8	5	1	5	7	8582.56767	8582.56706	0.00061
7	3	5	6	7	2	6	6	8661.82414	8661.82410	0.00004
7	3	5	9	7	2	6	9	8662.58249	8662.58252	−0.00003
7	3	5	7	7	2	6	7	8665.70953	8665.70980	−0.00027
7	3	5	8	7	2	6	8	8666.54547	8666.54492	0.00055
7	2	6	7	7	1	7	7	8693.26979	8693.27015	−0.00036
7	2	6	8	7	1	7	8	8694.38638	8694.38586	0.00052
3	2	2	5	2	1	1	4	9079.91580	9079.91509	0.00071
3	2	2	3	2	1	1	2	9080.16193	9080.16226	−0.00033
3	2	2	3	2	1	1	3	9080.91589	9080.91415	0.00174
3	2	2	4	2	1	1	3	9081.05003	9081.05015	−0.00012
6	1	6	6	5	0	5	5	9130.64836	9130.64830	0.00006
6	1	6	7	5	0	5	6	9131.01275	9131.01341	−0.00066
6	1	6	5	5	0	5	4	9131.52441	9131.52470	−0.00029
6	1	6	8	5	0	5	7	9131.86752	9131.86711	0.00041
8	3	6	7	8	2	7	7	9247.89161	9247.89134	0.00027
8	3	6	10	8	2	7	10	9248.53394	9248.53305	0.00089
8	3	6	8	8	2	7	8	9251.51075	9251.51097	−0.00022
8	3	6	9	8	2	7	9	9252.17170	9252.17159	0.00011
8	2	7	7	8	1	8	7	9818.47795	9818.47835	−0.0004
8	2	7	10	8	1	8	10	9819.34061	9819.34031	0.0003
8	2	7	8	8	1	8	8	9823.34488	9823.34437	0.00051
8	2	7	9	8	1	8	9	9824.24534	9824.24482	0.00052
7	0	7	7	6	1	6	6	10066.98192	10066.98279	−0.00087
7	0	7	6	6	1	6	5	10067.23629	10067.23396	0.00233

Table S14: Assignment in Pickett notation of experimentally determined transitions of c-3-^{35}ClBzA

J'	K_a'	K_c'	F'	J''	K_a''	K_c''	F''	ν_{obs} / MHz	ν_{calc} / MHz	$\nu_{obs - calc}$ / MHz
7	0	7	8	6	1	6	7	10067.34618	10067.34572	0.00046
7	0	7	9	6	1	6	8	10067.63484	10067.63378	0.00106
7	4	3	7	7	3	4	7	10068.63808	10068.63826	−0.00018
7	4	3	8	7	3	4	8	10069.10412	10069.10464	−0.00052
3	2	1	2	2	1	2	1	10091.41192	10091.41195	−0.00003
3	2	1	5	2	1	2	4	10097.91411	10097.91343	0.00068
3	2	1	4	2	1	2	4	10099.23293	10099.23378	−0.00085
3	2	1	2	2	1	2	2	10099.67245	10099.67330	−0.00085
3	2	1	3	2	1	2	2	10100.90742	10100.90738	0.00004
3	2	1	3	2	1	2	3	10106.86946	10106.86974	−0.00028
3	2	1	4	2	1	2	3	10107.49844	10107.49802	0.00042
4	2	3	5	3	1	2	5	10295.25546	10295.25637	−0.00091
4	2	3	4	3	1	2	3	10298.46447	10298.46505	−0.00058
4	2	3	3	3	1	2	2	10298.68054	10298.68095	−0.00041
4	2	3	5	3	1	2	4	10298.77398	10298.77265	0.00133
4	2	3	6	3	1	2	5	10299.10404	10299.10276	0.00128
4	2	3	4	3	1	2	4	10300.10233	10300.10312	−0.00079
4	2	3	3	3	1	2	3	10302.35769	10302.35886	−0.00117
6	4	2	5	6	3	3	5	10352.88969	10352.89021	−0.00052
6	4	2	8	6	3	3	8	10353.64773	10353.64695	0.00078
6	4	2	6	6	3	3	6	10356.65180	10356.65234	−0.00054
6	4	2	7	6	3	3	7	10357.71356	10357.71282	0.00074
7	1	7	7	6	0	6	6	10373.62976	10373.63061	−0.00085
7	1	7	8	6	0	6	7	10373.93829	10373.93753	0.00076
7	1	7	6	6	0	6	5	10374.25388	10374.25418	−0.0003
7	1	7	9	6	0	6	8	10374.54580	10374.54439	0.00141
9	1	8	11	9	0	9	11	10439.39741	10439.39654	0.00087
9	1	8	9	9	0	9	9	10443.51926	10443.51901	0.00025
9	1	8	10	9	0	9	10	10444.32057	10444.32168	−0.00111
5	4	1	4	5	3	2	4	10513.54700	10513.54732	−0.00032
5	4	1	7	5	3	2	7	10515.36209	10515.36155	0.00054
5	4	1	5	5	3	2	5	10519.74216	10519.74250	−0.00034
5	4	1	6	5	3	2	6	10521.29708	10521.29622	0.00086
4	4	0	3	4	3	1	3	10590.30685	10590.30702	−0.00017
4	4	0	6	4	3	1	6	10593.66608	10593.66541	0.00067
6	4	3	5	6	3	4	5	10597.16097	10597.16205	−0.00108
6	4	3	8	6	3	4	8	10598.12637	10598.12562	0.00075
5	4	2	4	5	3	3	4	10599.59354	10599.59358	−0.00004
4	4	0	4	4	3	1	4	10599.80839	10599.80861	−0.00022
5	4	2	7	5	3	3	7	10601.47789	10601.47706	0.00083

Table S14: Assignment in Pickett notation of experimentally determined transitions of c-3-^{35}ClBzA

J'	K_a'	K_c'	F'	J''	K_a''	K_c''	F''	ν_{obs} / MHz	ν_{calc} / MHz	$\nu_{obs - calc}$ / MHz
6	4	3	6	6	3	4	6	10601.62080	10601.62226	−0.00146
6	4	3	7	6	3	4	7	10602.80966	10602.81015	−0.00049
4	4	0	5	4	3	1	5	10603.07464	10603.07436	0.00028
5	4	2	5	5	3	3	5	10606.08925	10606.08702	0.00223
5	4	2	6	5	3	3	6	10607.76691	10607.76676	0.00015
4	4	1	6	4	3	2	5	10612.28779	10612.28598	0.00181
4	4	1	3	4	3	2	3	10612.54501	10612.54528	−0.00027
4	4	1	6	4	3	2	6	10616.05645	10616.05618	0.00027
4	4	1	4	4	3	2	4	10622.33285	10622.33298	−0.00013
4	4	1	5	4	3	2	5	10625.50709	10625.50699	0.0001
7	4	4	6	7	3	5	6	10626.86207	10626.86191	0.00016
7	4	4	9	7	3	5	9	10627.67547	10627.67406	0.00141
7	4	4	7	7	3	5	7	10630.48912	10630.49040	−0.00128
7	4	4	8	7	3	5	8	10631.15805	10631.15844	−0.00039
9	4	6	8	9	3	7	8	10898.27204	10898.27226	−0.00022
9	4	6	11	9	3	7	11	10898.65968	10898.65922	0.00046
9	4	6	9	9	3	7	9	10900.83694	10900.83709	−0.00015
9	4	6	10	9	3	7	10	10901.27527	10901.27487	0.0004
5	2	4	5	4	1	3	4	11384.00660	11384.00655	0.00005
5	2	4	6	4	1	3	5	11384.14064	11384.14054	0.0001
8	0	8	8	7	1	7	7	11499.59579	11499.59656	−0.00077
8	0	8	7	7	1	7	6	11499.84017	11499.84050	−0.00033
8	0	8	9	7	1	7	8	11499.87716	11499.87682	0.00034
8	0	8	10	7	1	7	9	11500.14471	11500.14389	0.00082
8	1	8	8	7	0	7	7	11663.10156	11663.10157	−0.00001
8	1	8	9	7	0	7	8	11663.36136	11663.36106	0.0003
8	1	8	7	7	0	7	6	11663.54534	11663.54634	−0.001
8	1	8	10	7	0	7	9	11663.79421	11663.79360	0.00061
8	1	7	8	7	2	6	7	11688.55027	11688.55040	−0.00013
8	1	7	7	7	2	6	6	11688.66981	11688.67026	−0.00045
8	1	7	9	7	2	6	8	11688.70925	11688.70903	0.00022
8	1	7	10	7	2	6	9	11688.87281	11688.87244	0.00037
4	2	2	3	3	1	3	2	12486.98457	12486.98555	−0.00098
4	2	2	5	3	1	3	4	12500.41081	12500.41047	0.00034
3	3	1	2	2	2	0	2	12524.17452	12524.17541	−0.00089
3	3	1	3	2	2	0	3	12528.87677	12528.87782	−0.00105
3	3	1	5	2	2	0	4	12532.48153	12532.48065	0.00088
3	3	1	2	2	2	0	1	12533.91859	12533.91910	−0.00051
3	3	1	4	2	2	0	3	12534.38833	12534.38796	0.00037
3	3	1	4	2	2	0	4	12544.31768	12544.31886	−0.00118

Table S14: Assignment in Pickett notation of experimentally determined transitions of c-3-^{35}ClBzA

J'	K_a'	K_c'	F'	J''	K_a''	K_c''	F''	ν_{obs} / MHz	ν_{calc} / MHz	$\nu_{\text{obs - calc}}$ / MHz
3	3	0	2	2	2	1	2	12565.07128	12565.07229	−0.00101
3	3	0	3	2	2	1	3	12570.38726	12570.38809	−0.00083
3	3	0	5	2	2	1	4	12573.36841	12573.36762	0.00079
3	3	0	2	2	2	1	1	12574.49637	12574.49660	−0.00023
3	3	0	4	2	2	1	3	12575.87778	12575.87756	0.00022
3	3	0	3	2	2	1	2	12576.98803	12576.98816	−0.00013
9	0	9	9	8	1	8	8	12899.95033	12899.95048	−0.00015
9	0	9	10	8	1	8	9	12900.17544	12900.17558	−0.00014
9	0	9	11	8	1	8	10	12900.41547	12900.41508	0.00039
9	1	9	9	8	0	8	8	12984.21374	12984.21365	0.00009
9	1	9	10	8	0	8	9	12984.43312	12984.43308	0.00004
9	1	9	8	8	0	8	7	12984.54125	12984.54148	−0.00023
9	1	9	11	8	0	8	10	12984.75316	12984.75256	0.0006
7	2	6	8	6	1	5	7	13272.59923	13272.59930	−0.00007
7	2	6	7	6	1	5	6	13272.69795	13272.69828	−0.00033
7	2	6	9	6	1	5	8	13274.18871	13274.18838	0.00033
7	2	6	6	6	1	5	5	13274.31140	13274.31218	−0.00078
8	5	4	7	8	4	5	7	13523.99201	13523.99328	−0.00127
8	5	4	10	8	4	5	10	13524.28324	13524.28283	0.00041
8	5	4	8	8	4	5	8	13526.99577	13526.99608	−0.00031
8	5	4	9	8	4	5	9	13527.82100	13527.82101	−0.00001
7	5	2	6	7	4	3	6	13539.76347	13539.76345	0.00002
7	5	2	9	7	4	3	9	13541.05306	13541.05282	0.00024
7	5	2	7	7	4	3	7	13544.52211	13544.52168	0.00043
7	5	2	8	7	4	3	8	13544.83426	13544.83494	−0.00068
7	5	3	6	7	4	4	6	13575.53214	13575.53212	0.00002
7	5	3	9	7	4	4	9	13576.63466	13576.63475	−0.00009
7	5	3	7	7	4	4	7	13580.12255	13580.12306	−0.00051
6	5	1	5	6	4	2	5	13607.71641	13607.71640	0.00001
6	5	1	8	6	4	2	8	13609.12239	13609.12175	0.00064
6	5	1	6	6	4	2	6	13613.53185	13613.53230	−0.00045
10	2	8	9	9	3	7	8	13679.40976	13679.40992	−0.00016
10	2	8	12	9	3	7	11	13679.55344	13679.55352	−0.00008
10	2	8	10	9	3	7	9	13679.64666	13679.64626	0.0004
10	2	8	11	9	3	7	10	13679.76074	13679.76107	−0.00033
4	3	2	4	3	2	1	4	14093.77665	14093.77737	−0.00072
4	3	2	4	3	2	1	3	14094.40585	14094.40565	0.0002
4	3	2	5	3	2	1	4	14095.24128	14095.24106	0.00022
4	3	2	5	3	2	1	5	14096.56021	14096.56141	−0.0012
8	2	7	9	7	1	6	8	14178.54061	14178.54126	−0.00065

Table S14: Assignment in Pickett notation of experimentally determined transitions of c-3-^{35}ClBzA

J'	K_a'	K_c'	F'	J''	K_a''	K_c''	F''	ν_{obs} / MHz	ν_{calc} / MHz	$\nu_{obs - calc}$ / MHz
8	2	7	8	7	1	6	7	14178.61542	14178.61450	0.00092
8	2	7	10	7	1	6	9	14180.01276	14180.01352	−0.00076
8	2	7	7	7	1	6	6	14180.10432	14180.10241	0.00191
10	0	10	10	9	1	9	9	14281.91695	14281.91685	0.0001
10	0	10	11	9	1	9	10	14282.10285	14282.10310	−0.00025
10	0	10	9	9	1	9	8	14282.11786	14282.11737	0.00049
10	0	10	12	9	1	9	11	14282.31302	14282.31284	0.00018
4	3	1	3	3	2	2	2	14298.67465	14298.67481	−0.00016
4	3	1	4	3	2	2	3	14302.55517	14302.55704	−0.00187
4	3	1	5	3	2	2	4	14303.79338	14303.79307	0.00031
10	1	10	10	9	0	9	9	14324.24901	14324.24918	−0.00017
10	1	10	11	9	0	9	10	14324.43476	14324.43506	−0.0003
10	1	10	9	9	0	9	8	14324.50071	14324.50085	−0.00014
10	1	10	12	9	0	9	11	14324.68231	14324.68177	0.00054
5	2	3	4	4	1	4	3	15275.61937	15275.61990	−0.00053
5	2	3	7	4	1	4	6	15279.05538	15279.05457	0.00081
5	2	3	5	4	1	4	4	15284.57138	15284.57130	0.00008
5	3	3	4	4	2	2	3	15519.77368	15519.77434	−0.00066
5	3	3	7	4	2	2	6	15520.17309	15520.17239	0.0007
5	3	3	5	4	2	2	4	15520.98726	15520.98734	−0.00008
5	3	3	6	4	2	2	5	15521.53135	15521.53109	0.00026
11	0	11	11	10	1	10	10	15653.93154	15653.93147	0.00007
11	0	11	12	10	1	10	11	15654.08795	15654.08888	−0.00093
11	0	11	10	10	1	10	9	15654.10780	15654.10733	0.00047
11	0	11	13	10	1	10	12	15654.27050	15654.27053	−0.00003
11	1	11	11	10	0	10	10	15674.78279	15674.78267	0.00012
11	1	11	12	10	0	10	11	15674.94080	15674.94103	−0.00023
11	1	11	10	10	0	10	9	15674.98325	15674.98331	−0.00006
11	1	11	13	10	0	10	12	15675.13933	15675.13927	0.00006
5	3	2	4	4	2	3	3	16134.13580	16134.13610	−0.0003
5	3	2	7	4	2	3	6	16135.46383	16135.46308	0.00075
5	3	2	5	4	2	3	4	16137.91349	16137.91369	−0.0002
5	3	2	6	4	2	3	5	16139.22791	16139.22820	−0.00029
10	2	9	10	9	1	8	9	16192.20053	16192.20165	−0.00112
10	2	9	11	9	1	8	10	16192.20915	16192.20860	0.00055
10	2	9	12	9	1	8	11	16193.17256	16193.17129	0.00127
6	3	4	5	5	2	3	4	16767.20090	16767.20154	−0.00064
6	3	4	8	5	2	3	7	16767.54113	16767.54055	0.00058
6	3	4	6	5	2	3	5	16767.58090	16767.58068	0.00022
6	3	4	7	5	2	3	6	16767.69973	16767.69983	−0.0001

Table S14: Assignment in Pickett notation of experimentally determined transitions of c-3-^{35}ClBzA

J'	K'_a	K'_c	F'	J''	K''_a	K''_c	F''	ν_{obs} / MHz	ν_{calc} / MHz	$\nu_{obs - calc}$ / MHz
11	1	10	11	10	2	9	10	16768.77212	16768.77290	−0.00078
11	1	10	12	10	2	9	11	16768.85130	16768.85165	−0.00035
11	1	10	10	10	2	9	9	16769.08937	16769.09086	−0.00149
11	1	10	13	10	2	9	12	16769.18725	16769.18649	0.00076
12	0	12	12	11	1	11	11	17020.76745	17020.76722	0.00023
12	0	12	13	11	1	11	12	17020.90183	17020.90231	−0.00048
12	0	12	11	11	1	11	10	17020.92093	17020.92046	0.00047
12	0	12	14	11	1	11	13	17021.05923	17021.05927	−0.00004
4	4	1	3	3	3	0	3	17232.90242	17232.90227	0.00015
4	4	1	6	3	3	0	5	17243.88075	17243.88006	0.00069

Table S15: Assignment in Pickett notation of experimentally determined transitions of c-3-^{37}ClBzA

J'	K'_a	K'_c	F'	J''	K''_a	K''_c	F''	ν_{obs} / MHz	ν_{calc} / MHz	$\nu_{obs - calc}$ / MHz
6	0	6	6	5	1	5	5	8402.11669	8402.11721	−0.00052
6	0	6	5	5	1	5	4	8402.31949	8402.31996	−0.00047
6	0	6	7	5	1	5	6	8402.50805	8402.50813	−0.00008
6	0	6	8	5	1	5	7	8402.75526	8402.75485	0.00041
6	1	6	6	5	0	5	5	8981.03446	8981.03524	−0.00078
6	1	6	7	5	0	5	6	8981.33765	8981.33671	0.00094
6	1	6	5	5	0	5	4	8981.68785	8981.68798	−0.00013
6	1	6	8	5	0	5	7	8981.97432	8981.97410	0.00022
3	2	2	5	2	1	1	4	8986.90231	8986.90144	0.00087
3	2	2	3	2	1	1	2	8987.18234	8987.18209	0.00025
3	2	2	4	2	1	1	3	8988.08382	8988.08383	−0.00001
9	4	5	8	9	3	6	8	9149.83313	9149.83355	−0.00042
9	4	5	11	9	3	6	11	9149.93249	9149.93253	−0.00004
8	3	6	7	8	2	7	7	9153.34074	9153.34157	−0.00083
8	3	6	10	8	2	7	10	9153.83996	9153.83947	0.00049
8	3	6	8	8	2	7	8	9156.15121	9156.15158	−0.00037
8	3	6	9	8	2	7	9	9156.66377	9156.66388	−0.00011
7	1	6	7	6	2	5	6	9413.08825	9413.08845	−0.0002
7	1	6	8	6	2	5	7	9413.20725	9413.20731	−0.00006
7	1	6	6	6	2	5	5	9413.31483	9413.31478	0.00005
7	1	6	9	6	2	5	8	9413.48639	9413.48611	0.00028
7	0	7	7	6	1	6	6	9866.71147	9866.71204	−0.00057
7	0	7	6	6	1	6	5	9866.90844	9866.90917	−0.00073
7	0	7	8	6	1	6	7	9867.00311	9867.00272	0.00039

Table S15: Assignment in Pickett notation of experimentally determined transitions of c-3-^{37}ClBzA

J'	K_a'	K_c'	F'	J''	K_a''	K_c''	F''	ν_{obs} / MHz	ν_{calc} / MHz	$\nu_{obs - calc}$ / MHz
7	0	7	9	6	1	6	8	9867.22408	9867.22377	0.00031
3	2	1	2	2	1	2	1	9960.85211	9960.85209	0.00002
3	2	1	5	2	1	2	4	9965.93644	9965.93588	0.00056
3	2	1	4	2	1	2	4	9966.87923	9966.87988	−0.00065
3	2	1	2	2	1	2	2	9967.36713	9967.36798	−0.00085
3	2	1	3	2	1	2	2	9968.25511	9968.25525	−0.00014
3	2	1	4	2	1	2	3	9973.39634	9973.39584	0.0005
4	2	3	3	3	1	2	2	10185.13636	10185.13753	−0.00117
4	2	3	4	3	1	2	3	10185.19822	10185.19789	0.00033
4	2	3	5	3	1	2	4	10185.57017	10185.57067	−0.0005
4	2	3	6	3	1	2	5	10185.58023	10185.57806	0.00217
7	1	7	7	6	0	6	6	10195.17537	10195.17591	−0.00054
7	1	7	8	6	0	6	7	10195.42178	10195.42286	−0.00108
7	1	7	6	6	0	6	5	10195.64786	10195.64743	0.00043
7	1	7	9	6	0	6	8	10195.88371	10195.88366	0.00005
5	4	1	5	5	3	2	5	10523.35193	10523.35200	−0.00007
5	4	1	6	5	3	2	6	10524.70524	10524.70599	−0.00075
6	4	3	5	6	3	4	5	10592.10972	10592.10949	0.00023
4	4	0	6	4	3	1	6	10592.23197	10592.23118	0.00079
6	4	3	8	6	3	4	8	10592.93093	10592.93067	0.00026
6	4	3	6	6	3	4	6	10595.79434	10595.79502	−0.00068
5	4	2	4	5	3	3	4	10596.39813	10596.39767	0.00046
6	4	3	7	6	3	4	7	10596.74927	10596.74927	0.0000
4	4	0	4	4	3	1	4	10597.28958	10597.29027	−0.00069
5	4	2	7	5	3	3	7	10597.92009	10597.91875	0.00134
7	4	4	6	7	3	5	6	10616.61695	10616.61726	−0.00031
5	2	4	5	4	1	3	4	11254.33304	11254.33333	−0.00029
5	2	4	6	4	1	3	5	11254.55158	11254.55158	0.0000
5	2	4	7	4	1	3	6	11255.07857	11255.07805	0.00052
5	2	4	4	4	1	3	3	11255.17225	11255.17254	−0.00029
8	0	8	8	7	1	7	7	11277.94807	11277.94816	−0.00009
8	0	8	7	7	1	7	6	11278.13523	11278.13601	−0.00078
8	0	8	9	7	1	7	8	11278.17284	11278.17274	0.0001
8	0	8	10	7	1	7	9	11278.37555	11278.37555	0.0000
8	1	7	8	7	2	6	7	11377.58984	11377.58963	0.00021
8	1	7	9	7	2	6	8	11377.71436	11377.71469	−0.00033
8	1	7	7	7	2	6	6	11377.72352	11377.72301	0.00051
8	1	7	10	7	2	6	9	11377.87668	11377.87672	−0.00004
8	1	8	8	7	0	7	7	11455.80047	11455.80139	−0.00092
8	1	8	9	7	0	7	8	11456.00845	11456.00826	0.00019

Table S15: Assignment in Pickett notation of experimentally determined transitions of c-3-^{37}ClBzA

J'	K_a'	K_c'	F'	J''	K_a''	K_c''	F''	ν_{obs} / MHz	ν_{calc} / MHz	$\nu_{obs - calc}$ / MHz
8	1	8	7	7	0	7	6	11456.14069	11456.14080	−0.00011
8	1	8	10	7	0	7	9	11456.34010	11456.33983	0.00027
6	2	5	7	5	1	4	6	12216.74176	12216.73990	0.00186
6	2	5	8	5	1	4	7	12217.73996	12217.74046	−0.0005
6	2	5	5	5	1	4	4	12217.77843	12217.77915	−0.00072
4	2	2	3	3	1	3	2	12287.76800	12287.76782	0.00018
4	2	2	6	3	1	3	5	12291.14969	12291.14922	0.00047
4	2	2	4	3	1	3	3	12294.65116	12294.65094	0.00022
9	0	9	9	8	1	8	8	12655.82177	12655.82200	−0.00023
9	0	9	8	8	1	8	7	12655.99391	12655.99416	−0.00025
9	0	9	10	8	1	8	9	12656.00345	12656.00208	0.00137
9	0	9	11	8	1	8	10	12656.18386	12656.18380	0.00006
9	1	9	9	8	0	8	8	12748.88650	12748.88689	−0.00039
9	1	9	10	8	0	8	9	12749.06041	12749.06118	−0.00077
9	1	9	8	8	0	8	7	12749.13944	12749.13820	0.00124
9	1	9	11	8	0	8	10	12749.30715	12749.30705	0.0001
7	2	6	8	6	1	5	7	13112.01615	13112.01593	0.00022
7	2	6	7	6	1	5	6	13112.05906	13112.05923	−0.00017
7	2	6	9	6	1	5	8	13113.14162	13113.14135	0.00027
7	2	6	6	6	1	5	5	13113.20152	13113.20158	−0.00006
9	1	8	9	8	2	7	8	13196.48800	13196.48822	−0.00022
9	1	8	10	8	2	7	9	13196.59242	13196.59237	0.00005
9	1	8	8	8	2	7	7	13196.64634	13196.64616	0.00018
9	1	8	11	8	2	7	10	13196.77005	13196.76969	0.00036
9	5	4	8	9	4	5	8	13251.40362	13251.40343	0.00019
9	5	4	11	9	4	5	11	13251.74751	13251.74691	0.0006
11	5	7	10	11	4	8	10	13433.92753	13433.92870	−0.00117
11	5	7	13	11	4	8	13	13434.11258	13434.11256	0.00002
8	5	3	7	8	4	4	7	13434.64417	13434.64490	−0.00073
8	5	3	10	8	4	4	10	13435.00422	13435.00380	0.00042
11	5	7	11	11	4	8	11	13435.44648	13435.44619	0.00029
10	5	6	9	10	4	7	9	13435.63650	13435.63748	−0.00098
11	5	7	12	11	4	8	12	13435.66432	13435.66393	0.00039
10	5	6	12	10	4	7	12	13436.09836	13436.09654	0.00182
8	5	3	8	8	4	4	8	13437.12142	13437.12144	−0.00002
8	5	3	9	8	4	4	9	13437.66899	13437.66946	−0.00047
4	3	2	3	3	2	1	3	13954.05289	13954.05418	−0.00129
4	3	2	3	3	2	1	2	13954.94102	13954.94145	−0.00043
4	3	2	6	3	2	1	5	13955.41610	13955.41532	0.00078
4	3	2	4	3	2	1	4	13956.32194	13956.32243	−0.00049

Table S15: Assignment in Pickett notation of experimentally determined transitions of c-3-^{37}ClBzA

J'	K_a'	K_c'	F'	J''	K_a''	K_c''	F''	ν_{obs} / MHz	ν_{calc} / MHz	$\nu_{obs - calc}$ / MHz
4	3	2	4	3	2	1	3	13956.77117	13956.77108	0.00009
4	3	2	5	3	2	1	4	13957.54712	13957.54685	0.00027
4	3	2	5	3	2	1	5	13958.48960	13958.49085	−0.00125
8	2	7	9	7	1	6	8	13995.87349	13995.87365	−0.00016
8	2	7	8	7	1	6	7	13995.91073	13995.91012	0.00061
8	2	7	10	7	1	6	9	13996.94729	13996.94714	0.00015
8	2	7	7	7	1	6	6	13996.99380	13996.99409	−0.00029
10	0	10	10	9	1	9	9	14014.33649	14014.33668	−0.00019
10	0	10	11	9	1	9	10	14014.48511	14014.48535	−0.00024
10	0	10	9	9	1	9	8	14014.49046	14014.49010	0.00036
10	0	10	12	9	1	9	11	14014.64462	14014.64481	−0.00019
10	1	10	10	9	0	9	9	14061.80290	14061.80280	0.0001
10	1	10	11	9	0	9	10	14061.95014	14061.95027	−0.00013
10	1	10	9	9	0	9	8	14061.99556	14061.99610	−0.00054
10	1	10	12	9	0	9	11	14062.13991	14062.14024	−0.00033
4	3	1	3	3	2	2	2	14147.86598	14147.86616	−0.00018
4	3	1	6	3	2	2	5	14148.98851	14148.98802	0.00049
4	3	1	4	3	2	2	3	14150.98346	14150.98348	−0.00002
4	3	1	5	3	2	2	4	14151.98379	14151.98364	0.00015
10	1	9	10	9	2	8	9	14869.87139	14869.87230	−0.00091
10	1	9	11	9	2	8	10	14869.95542	14869.95470	0.00072
10	1	9	9	9	2	8	8	14870.07851	14870.07896	−0.00045
10	1	9	12	9	2	8	11	14870.17636	14870.17570	0.00066
9	2	8	10	8	1	7	9	14926.80399	14926.80459	−0.0006
9	2	8	9	8	1	7	8	14926.81828	14926.81749	0.00079
9	2	8	11	8	1	7	10	14927.72193	14927.72220	−0.00027
9	2	8	8	8	1	7	7	14927.74279	14927.74260	0.00019
5	2	3	4	4	1	4	3	14991.71850	14991.71885	−0.00035
5	2	3	7	4	1	4	6	14994.35253	14994.35315	−0.00062
5	2	3	6	4	1	4	5	15001.06116	15001.06061	0.00055
5	3	3	4	4	2	2	3	15360.52546	15360.52604	−0.00058
5	3	3	7	4	2	2	6	15360.93586	15360.93532	0.00054
5	3	3	5	4	2	2	4	15361.69348	15361.69361	−0.00013
5	3	3	6	4	2	2	5	15362.18020	15362.18038	−0.00018
11	0	11	11	10	1	10	10	15362.19262	15362.19215	0.00047
11	0	11	13	10	1	10	12	15362.45592	15362.45596	−0.00004
11	1	11	11	10	0	10	10	15385.92667	15385.92609	0.00058
11	1	11	12	10	0	10	11	15386.05086	15386.05168	−0.00082
11	1	11	10	10	0	10	9	15386.08108	15386.08030	0.00078
11	1	11	13	10	0	10	12	15386.20462	15386.20420	0.00042

Table S15: Assignment in Pickett notation of experimentally determined transitions of c-3-^{37}ClBzA

J'	K'_a	K'_c	F'	J''	K''_a	K''_c	F''	ν_{obs} / MHz	ν_{calc} / MHz	$\nu_{obs - calc}$ / MHz
5	3	2	4	4	2	3	3	15935.13531	15935.13589	−0.00058
5	3	2	5	4	2	3	4	15938.12700	15938.12638	0.00062
10	2	9	10	9	1	8	9	15944.48331	15944.48392	−0.00061
10	2	9	11	9	1	8	10	15944.49609	15944.49546	0.00063
10	2	9	9	9	1	8	8	15945.22035	15945.22074	−0.00039
10	2	9	12	9	1	8	11	15945.22681	15945.22624	0.00057
11	1	10	11	10	2	9	10	16423.61643	16423.61663	−0.0002
11	1	10	12	10	2	9	11	16423.68366	16423.68309	0.00057
11	1	10	10	10	2	9	9	16423.85556	16423.85667	−0.00111
11	1	10	13	10	2	9	12	16423.93501	16423.93397	0.00104
6	3	4	5	5	2	3	4	16594.29278	16594.29318	−0.0004
6	3	4	8	5	2	3	7	16594.58657	16594.58614	0.00043
6	3	4	6	5	2	3	5	16594.75549	16594.75519	0.0003
6	3	4	7	5	2	3	6	16594.93300	16594.93284	0.00016
12	0	12	12	11	1	11	11	16704.40539	16704.40597	−0.00058
12	0	12	13	11	1	11	12	16704.51255	16704.51339	−0.00084
12	0	12	11	11	1	11	10	16704.52432	16704.52338	0.00094
12	0	12	14	11	1	11	13	16704.63340	16704.63325	0.00015
12	1	12	12	11	0	11	11	16716.08692	16716.08717	−0.00025
12	1	12	13	11	0	11	12	16716.19466	16716.19506	−0.0004
12	1	12	11	11	0	11	10	16716.21503	16716.21402	0.00101
12	1	12	14	11	0	11	13	16716.32113	16716.32129	−0.00016
11	2	10	11	10	1	9	10	17057.48998	17057.49037	−0.00039
11	2	10	12	10	1	9	11	17057.51925	17057.51917	0.00008
11	2	10	10	10	1	9	9	17058.05962	17058.06002	−0.0004
11	2	10	13	10	1	9	12	17058.08461	17058.08376	0.00085
4	4	1	6	3	3	0	5	17097.93738	17097.93678	0.0006
4	4	0	4	3	3	1	4	17098.42448	17098.42558	−0.0011
4	4	1	3	3	3	0	2	17098.68735	17098.68725	0.0001
4	4	1	5	3	3	0	4	17099.05388	17099.05396	−0.00008
4	4	1	4	3	3	0	3	17099.76765	17099.76798	−0.00033
4	4	0	6	3	3	1	5	17101.08867	17101.08809	0.00058
4	4	0	3	3	3	1	2	17101.81810	17101.81848	−0.00038
4	4	0	5	3	3	1	4	17102.22166	17102.22140	0.00026
4	4	0	4	3	3	1	3	17102.93867	17102.93877	−0.0001
7	3	5	7	6	2	4	6	17643.12815	17643.12762	0.00053
7	3	5	8	6	2	4	7	17643.22151	17643.22169	−0.00018
7	3	5	6	6	2	4	5	17643.40200	17643.40339	−0.00139
7	3	5	9	6	2	4	8	17643.42157	17643.42080	0.00077
12	1	11	12	11	2	10	11	17891.65386	17891.65533	−0.00147

Table S15: Assignment in Pickett notation of experimentally determined transitions of c-3-^{37}ClBzA

J'	K_a'	K_c'	F'	J''	K_a''	K_c''	F''	ν_{obs} / MHz	ν_{calc} / MHz	$\nu_{obs - calc}$ / MHz
12	1	11	13	11	2	10	12	17891.71192	17891.71139	0.00053
12	1	11	14	11	2	10	13	17891.97071	17891.97079	−0.00008
6	3	3	5	5	2	4	4	17894.81359	17894.81326	0.00033
6	3	3	8	5	2	4	7	17896.02118	17896.02040	0.00078

Table S16: Assignment in Pickett notation of experimentally determined transitions of t-3-^{35}ClBzA

J'	K_a'	K_c'	F'	J''	K_a''	K_c''	F''	ν_{obs} / MHz	ν_{calc} / MHz	$\nu_{obs - calc}$ / MHz
6	1	6	6	5	1	5	5	8203.18937	8203.18935	0.00002
6	1	6	5	5	1	5	4	8203.53474	8203.53464	0.0001
6	1	6	7	5	1	5	6	8203.65614	8203.65643	−0.00029
6	0	6	6	5	0	5	5	8487.15781	8487.15822	−0.00041
6	0	6	5	5	0	5	4	8487.39946	8487.39975	−0.00029
6	0	6	7	5	0	5	6	8487.72969	8487.72946	0.00023
6	0	6	8	5	0	5	7	8487.98043	8487.98020	0.00023
6	2	5	6	5	2	4	5	8722.46247	8722.46244	0.00003
6	2	5	7	5	2	4	6	8722.69126	8722.69046	0.0008
6	2	5	8	5	2	4	7	8724.07468	8724.07452	0.00016
6	2	5	5	5	2	4	4	8724.24683	8724.24710	−0.00027
6	3	4	7	5	3	3	6	8798.59774	8798.59809	−0.00035
6	3	4	6	5	3	3	5	8799.34784	8799.34771	0.00013
6	3	4	8	5	3	3	7	8801.95100	8801.95050	0.0005
6	3	4	5	5	3	3	4	8802.66297	8802.66218	0.00079
6	3	3	8	5	3	2	7	8816.81026	8816.80998	0.00028
6	3	3	5	5	3	2	4	8817.50405	8817.50407	−0.00002
6	2	4	6	5	2	3	5	8996.29776	8996.29900	−0.00124
6	2	4	7	5	2	3	6	8996.31054	8996.30996	0.00058
6	2	4	5	5	2	3	4	8997.57557	8997.57636	−0.00079
6	2	4	8	5	2	3	7	8997.62301	8997.62282	0.00019
6	1	5	6	5	1	4	5	9183.07215	9183.07244	−0.00029
6	1	5	7	5	1	4	6	9183.40328	9183.40329	−0.00001
6	1	5	5	5	1	4	4	9183.49396	9183.49454	−0.00058
6	1	5	8	5	1	4	7	9183.89598	9183.89572	0.00026
7	1	7	7	6	1	6	6	9546.16833	9546.16855	−0.00022
7	1	7	6	6	1	6	5	9546.70425	9546.70391	0.00034
7	1	7	8	6	1	6	7	9546.74155	9546.74133	0.00022
7	1	7	9	6	1	6	8	9546.85750	9546.85736	0.00014
6	1	6	6	5	0	5	5	9634.26317	9634.26344	−0.00027

Table S16: Assignment in Pickett notation of experimentally determined transitions of t-3-^{35}ClBzA

J'	K_a'	K_c'	F'	J''	K_a''	K_c''	F''	ν_{obs} / MHz	ν_{calc} / MHz	$\nu_{obs - calc}$ / MHz
6	1	6	5	5	0	5	4	9634.50028	9634.50107	−0.00079
6	1	6	7	5	0	5	6	9634.70552	9634.70576	−0.00024
6	1	6	8	5	0	5	7	9635.20113	9635.20094	0.00019
7	0	7	7	6	0	6	6	9807.04936	9807.04943	−0.00007
7	0	7	6	6	0	6	5	9807.29421	9807.29437	−0.00016
7	0	7	8	6	0	6	7	9807.45712	9807.45711	0.00001
7	0	7	9	6	0	6	8	9807.71008	9807.70982	0.00026
7	2	6	7	6	2	5	6	10155.82088	10155.82113	−0.00025
7	2	6	8	6	2	5	7	10155.85376	10155.85393	−0.00017
7	2	6	6	6	2	5	5	10156.72754	10156.72762	−0.00008
7	2	6	9	6	2	5	8	10156.90793	10156.90782	0.00011
2	2	1	2	1	1	0	2	10158.07671	10158.07709	−0.00038
2	2	1	1	1	1	0	1	10160.08608	10160.08648	−0.0004
2	2	1	4	1	1	0	3	10162.57452	10162.57303	0.00149
2	2	1	3	1	1	0	2	10167.44909	10167.44920	−0.00011
2	2	1	2	1	1	0	1	10173.13096	10173.13153	−0.00057
2	2	1	3	1	1	0	3	10175.68622	10175.68618	0.00004
8	0	8	7	7	1	7	6	10218.41575	10218.41722	−0.00147
8	0	8	8	7	1	7	7	10218.46802	10218.46778	0.00024
8	0	8	9	7	1	7	8	10218.74362	10218.74330	0.00032
8	0	8	10	7	1	7	9	10218.87244	10218.87188	0.00056
7	4	4	8	6	4	3	7	10261.06035	10261.06066	−0.00031
7	4	3	7	6	4	2	6	10262.73601	10262.73614	−0.00013
7	4	4	9	6	4	3	8	10264.78005	10264.78007	−0.00002
7	4	4	6	6	4	3	5	10265.58870	10265.58928	−0.00058
7	4	3	9	6	4	2	8	10265.62510	10265.62486	0.00024
7	3	5	8	6	3	4	7	10274.66258	10274.66261	−0.00003
7	3	5	7	6	3	4	6	10274.98807	10274.98822	−0.00015
7	3	5	9	6	3	4	8	10276.77477	10276.77428	0.00049
7	3	5	6	6	3	4	5	10277.01735	10277.01757	−0.00022
7	3	4	8	6	3	3	7	10307.75374	10307.75354	0.0002
7	3	4	7	6	3	3	6	10307.89874	10307.89872	0.00002
7	2	5	7	6	2	4	6	10569.19498	10569.19503	−0.00005
7	2	5	8	6	2	4	7	10569.30099	10569.30053	0.00046
7	2	5	6	6	2	4	5	10569.90655	10569.90641	0.00014
7	2	5	9	6	2	4	8	10570.06977	10570.06959	0.00018
7	1	6	7	6	1	5	6	10673.54105	10673.54126	−0.00021
7	1	6	8	6	1	5	7	10673.81177	10673.81222	−0.00045
7	1	6	6	6	1	5	5	10673.87370	10673.87310	0.0006
7	1	6	9	6	1	5	8	10674.16505	10674.16446	0.00059

Table S16: Assignment in Pickett notation of experimentally determined transitions of t-3-^{35}ClBzA

J'	K_a'	K_c'	F'	J''	K_a''	K_c''	F''	ν_{obs} / MHz	ν_{calc} / MHz	$\nu_{obs - calc}$ / MHz
8	1	8	8	7	1	7	7	10880.70778	10880.70782	−0.00004
8	1	8	7	7	1	7	6	10880.86287	10880.86317	−0.0003
8	1	8	9	7	1	7	8	10880.97219	10880.97200	0.00019
8	1	8	10	7	1	7	9	10881.25639	10881.25576	0.00063
8	0	8	8	7	0	7	7	11104.69201	11104.69211	−0.0001
8	0	8	7	7	0	7	6	11104.92710	11104.92808	−0.00098
8	0	8	9	7	0	7	8	11105.00463	11105.00383	0.0008
8	0	8	10	7	0	7	9	11105.24091	11105.24016	0.00075
8	2	7	8	7	2	6	7	11579.24251	11579.24299	−0.00048
8	2	7	9	7	2	6	8	11579.33380	11579.33390	−0.0001
8	2	7	7	7	2	6	6	11579.87488	11579.87486	0.00002
8	2	7	10	7	2	6	9	11580.06459	11580.06424	0.00035
8	4	5	9	7	4	4	8	11738.70898	11738.70898	0.0000
8	4	5	8	7	4	4	7	11739.19898	11739.19859	0.00039
8	4	4	9	7	4	3	8	11741.02462	11741.02413	0.00049
8	4	5	10	7	4	4	9	11741.18185	11741.18163	0.00022
8	4	4	8	7	4	3	7	11741.50908	11741.50862	0.00046
8	4	5	7	7	4	4	6	11741.63891	11741.64097	−0.00206
8	4	4	10	7	4	3	9	11743.49431	11743.49385	0.00046
8	4	4	7	7	4	3	6	11743.94718	11743.94701	0.00017
8	3	6	8	7	3	5	7	11751.14740	11751.14767	−0.00027
8	3	6	9	7	3	5	8	11751.28050	11751.28060	−0.0001
8	1	8	8	7	0	7	7	11766.93211	11766.93215	−0.00004
8	1	8	9	7	0	7	8	11767.23270	11767.23253	0.00017
8	1	8	7	7	0	7	6	11767.37389	11767.37403	−0.00014
8	1	8	10	7	0	7	9	11767.62473	11767.62404	0.00069
8	3	5	10	7	3	4	9	11817.72935	11817.72949	−0.00014
8	3	5	7	7	3	4	6	11817.73869	11817.73826	0.00043
8	1	7	8	7	1	6	7	12141.85830	12141.85853	−0.00023
8	1	7	9	7	1	6	8	12142.07330	12142.07367	−0.00037
8	1	7	7	7	1	6	6	12142.14204	12142.14162	0.00042
8	1	7	10	7	1	6	9	12142.36820	12142.36770	0.0005
8	2	6	8	7	2	5	7	12153.86370	12153.86424	−0.00054
8	2	6	9	7	2	5	8	12154.11400	12154.11390	0.0001
8	2	6	7	7	2	5	6	12154.44770	12154.44927	−0.00157
8	2	6	10	7	2	5	9	12154.46323	12154.46156	0.00167
9	1	9	9	8	1	8	8	12207.41145	12207.41141	0.00004
9	1	9	8	8	1	8	7	12207.57343	12207.57492	−0.00149
9	1	9	10	8	1	8	9	12207.65644	12207.65603	0.00041
9	1	9	11	8	1	8	10	12207.84735	12207.84711	0.00024

Table S17: Assignment in Pickett notation of experimentally determined transitions of t-3-^{37}ClBzA

J'	K_a'	K_c'	F'	J''	K_a''	K_c''	F''	ν_{obs} / MHz	ν_{calc} / MHz	$\nu_{obs - calc}$ / MHz
5	1	4	5	4	1	3	4	7504.77586	7504.77532	0.00054
5	1	4	6	4	1	3	5	7505.18101	7505.18135	−0.00034
5	1	4	4	4	1	3	3	7505.42298	7505.42325	−0.00027
5	1	4	7	4	1	3	6	7505.69703	7505.69640	0.00063
6	0	6	6	5	0	5	5	8315.77985	8315.77976	0.00009
6	0	6	5	5	0	5	4	8315.95599	8315.95629	−0.0003
6	0	6	7	5	0	5	6	8316.23740	8316.23748	−0.00008
6	0	6	8	5	0	5	7	8316.42035	8316.41976	0.00059
6	2	4	6	5	2	3	5	8791.09093	8791.09206	−0.00113
6	2	4	7	5	2	3	6	8791.10337	8791.10262	0.00075
6	1	5	6	5	1	4	5	8980.67048	8980.67065	−0.00017
6	1	5	7	5	1	4	6	8980.93993	8980.94036	−0.00043
6	1	5	8	5	1	4	7	8981.32480	8981.32524	−0.00044
7	1	7	7	6	1	6	6	9352.52323	9352.52325	−0.00002
7	1	7	6	6	1	6	5	9352.90650	9352.90740	−0.0009
7	1	7	8	6	1	6	7	9352.95569	9352.95462	0.00107
7	1	7	9	6	1	6	8	9353.06862	9353.06862	0.0000
7	0	7	7	6	0	6	6	9612.17049	9612.17059	−0.0001
7	0	7	6	6	0	6	5	9612.35102	9612.35121	−0.00019
7	0	7	8	6	0	6	7	9612.49717	9612.49723	−0.00006
7	0	7	9	6	0	6	8	9612.68257	9612.68248	0.00009
7	2	6	7	6	2	5	6	9939.16548	9939.16639	−0.00091
7	2	6	8	6	2	5	7	9939.18666	9939.18667	−0.00001
7	2	6	6	6	2	5	5	9939.87989	9939.87977	0.00012
7	2	6	9	6	2	5	8	9940.02889	9940.02849	0.0004
7	2	5	7	6	2	4	6	10326.18419	10326.18497	−0.00078
7	2	5	8	6	2	4	7	10326.27657	10326.27586	0.00071
7	2	5	6	6	2	4	5	10326.77375	10326.77382	−0.00007
7	2	5	9	6	2	4	8	10326.89413	10326.89346	0.00067
7	1	6	7	6	1	5	6	10440.50642	10440.50632	0.0001
7	1	6	8	6	1	5	7	10440.72730	10440.72750	−0.0002
7	1	6	6	6	1	5	5	10440.76393	10440.76353	0.0004
7	1	6	9	6	1	5	8	10440.99863	10440.99832	0.00031
8	1	8	8	7	1	7	7	10661.10292	10661.10262	0.0003
8	1	8	7	7	1	7	6	10661.21304	10661.21328	−0.00024
8	1	8	9	7	1	7	8	10661.30409	10661.30416	−0.00007
8	1	8	10	7	1	7	9	10661.53414	10661.53368	0.00046
8	0	8	8	7	0	7	7	10886.54838	10886.54841	−0.00003
8	0	8	7	7	0	7	6	10886.72302	10886.72385	−0.00083
8	0	8	9	7	0	7	8	10886.79817	10886.79746	0.00071

Table S17: Assignment in Pickett notation of experimentally determined transitions of t-3-^{37}ClBzA

J'	K_a'	K_c'	F'	J''	K_a''	K_c''	F''	ν_{obs} / MHz	ν_{calc} / MHz	$\nu_{\mathrm{obs\ -\ calc}}$ / MHz
8	0	8	10	7	0	7	9	10886.97283	10886.97254	0.00029
8	2	7	8	7	2	6	7	11333.52701	11333.52730	−0.00029
8	2	7	9	7	2	6	8	11333.61135	11333.61133	0.00002
8	2	7	7	7	2	6	6	11334.04131	11334.04137	−0.00006
8	2	7	10	7	2	6	9	11334.17828	11334.17812	0.00016
8	2	6	8	7	2	5	7	11873.61222	11873.61168	0.00054
8	2	6	9	7	2	5	8	11873.83686	11873.83646	0.0004
8	2	6	10	7	2	5	9	11874.09520	11874.09542	−0.00022
8	2	6	7	7	2	5	6	11874.11569	11874.11509	0.0006
8	1	7	8	7	1	6	7	11879.98252	11879.98285	−0.00033
8	1	7	9	7	1	6	8	11880.15827	11880.15856	−0.00029
8	1	7	7	7	1	6	6	11880.19869	11880.19847	0.00022
8	1	7	10	7	1	6	9	11880.38115	11880.38124	−0.00009
9	2	8	9	8	2	7	8	12718.08394	12718.08377	0.00017
9	2	8	10	8	2	7	9	12718.47432	12718.47461	−0.00029
9	2	8	11	8	2	7	10	12718.57865	12718.57837	0.00028
9	2	8	8	8	2	7	7	12718.79388	12718.79406	−0.00018
10	1	10	10	9	1	9	9	13256.48302	13256.48283	0.00019
10	1	10	9	9	1	9	8	13256.59097	13256.59221	−0.00124
10	1	10	11	9	1	9	10	13256.64601	13256.64586	0.00015
10	1	10	12	9	1	9	11	13256.76352	13256.76349	0.00003
9	1	8	9	8	1	7	8	13294.47553	13294.47536	0.00017
9	1	8	10	8	1	7	9	13294.61564	13294.61597	−0.00033
9	1	8	8	8	1	7	7	13294.67323	13294.67313	0.0001
9	1	8	11	8	1	7	10	13294.81824	13294.81832	−0.00008
10	0	10	10	9	0	9	9	13400.78203	13400.78148	0.00055
10	0	10	9	9	0	9	8	13400.92987	13400.93031	−0.00044
10	0	10	11	9	0	9	10	13400.96104	13400.96104	0.0000
10	0	10	12	9	0	9	11	13401.10151	13401.10146	0.00005
9	2	7	9	8	2	6	8	13423.61093	13423.61139	−0.00046
9	2	7	10	8	2	6	9	13423.64485	13423.64408	0.00077
9	2	7	8	8	2	6	7	13423.75538	13423.75677	−0.00139
9	2	7	11	8	2	6	10	13423.97413	13423.97407	0.00006
11	1	11	11	10	1	10	10	14544.91520	14544.91590	−0.0007
11	1	11	10	10	1	10	9	14545.01099	14545.01020	0.00079
11	1	11	12	10	1	10	11	14545.05256	14545.05301	−0.00045
11	1	11	13	10	1	10	12	14545.15242	14545.15184	0.00058
11	0	11	11	10	0	10	10	14653.35484	14653.35431	0.00053
11	0	11	10	10	0	10	9	14653.47236	14653.47366	−0.0013
11	0	11	12	10	0	10	11	14653.48996	14653.48887	0.00109

Table S17: Assignment in Pickett notation of experimentally determined transitions of t-3-^{37}ClBzA

J'	K_a'	K_c'	F'	J''	K_a''	K_c''	F''	ν_{obs} / MHz	ν_{calc} / MHz	$\nu_{obs - calc}$ / MHz
11	0	11	13	10	0	10	12	14653.60897	14653.60926	−0.00029
11	1	10	13	10	1	9	12	16032.32539	16032.32497	0.00042
3	3	1	5	2	2	0	4	16480.20189	16480.20123	0.00066
3	3	1	4	2	2	0	3	16482.76448	16482.76514	−0.00066

6.3 2-ClBzA

Table S18: Assignment in Pickett notation of experimentally determined transitions of t-2-^{35}ClBzA

J'	K_a'	K_c'	F'	J''	K_a''	K_c''	F''	ν_{obs} / MHz	ν_{calc} / MHz	$\nu_{obs - calc}$ / MHz
5	0	5	7	4	1	4	6	8580.92370	8580.92286	0.00084
4	2	3	5	3	1	2	4	8581.19951	8581.19896	0.00055
4	1	3	4	3	1	2	3	8581.64599	8581.64546	0.00053
4	2	3	4	3	1	2	3	8581.77734	8581.77701	0.00033
4	2	3	4	3	1	2	4	8582.29490	8582.29647	−0.00157
4	2	3	6	3	1	2	5	8583.28132	8583.28094	0.00038
4	1	3	3	3	1	2	2	8583.83539	8583.83498	0.00041
4	2	3	3	3	1	2	2	8583.94262	8583.94215	0.00047
4	1	3	3	3	1	2	3	8584.90785	8584.90798	−0.00013
4	2	3	3	3	1	2	3	8585.01401	8585.01515	−0.00114
5	0	5	4	4	1	4	4	8591.93367	8591.93381	−0.00014
3	3	1	2	2	2	0	2	8660.02009	8660.02164	−0.00155
3	3	1	2	2	2	0	1	8668.52762	8668.52730	0.00032
3	3	1	5	2	2	0	4	8670.22244	8670.22206	0.00038
3	3	1	3	2	2	0	2	8676.89314	8676.89252	0.00062
3	3	1	4	2	2	0	3	8678.86649	8678.86601	0.00048
3	3	1	4	2	2	0	4	8687.53429	8687.53499	−0.0007
4	2	2	4	3	3	1	4	10020.81182	10020.81229	−0.00047
4	2	2	5	3	3	1	4	10021.30533	10021.30441	0.00092
4	2	2	4	3	3	1	3	10029.00251	10029.00276	−0.00025
4	3	2	5	3	3	1	4	10036.66892	10036.66923	−0.00031
4	2	2	6	3	3	1	5	10037.17759	10037.17619	0.0014
4	3	2	4	3	3	1	3	10043.64846[1]	10043.64899	−0.00053
4	2	2	3	3	3	1	2	10044.41758[1]	10044.41893	−0.00135
4	3	2	3	3	3	1	2	10057.18536	10057.18521	0.00015
3	3	0	3	2	2	1	3	10088.10090	10088.10070	0.0002
3	3	0	2	2	2	1	2	10090.03980	10090.04051	−0.00071

[1]Measured at the COBRA in Hannover.

Table S18: Assignment in Pickett notation of experimentally determined transitions of t-2-^{35}ClBzA

J'	K_a'	K_c'	F'	J''	K_a''	K_c''	F''	ν_{obs} / MHz	ν_{calc} / MHz	$\nu_{obs - calc}$ / MHz
3	3	0	4	2	2	1	3	10092.51457	10092.51448	0.00009
3	3	0	5	2	2	1	4	10098.49182	10098.49058	0.00124
3	3	0	3	2	2	1	2	10099.18944	10099.18932	0.00012
3	3	0	2	2	2	1	1	10103.96630	10103.96637	−0.00007
3	3	0	4	2	2	1	4	10107.80184	10107.80265	−0.00081
8	3	6	10	8	2	7	10	10133.56472	10133.56403	0.00069
7	2	6	9	7	1	7	9	10133.73718	10133.73721	−0.00003
7	1	6	7	7	0	7	7	10138.17082	10138.17022	0.0006
5	1	4	6	4	2	3	5	10138.73264	10138.73129	0.00135
6	0	6	7	5	1	5	6	10140.33799	10140.33742	0.00057
6	1	6	5	5	0	5	4	10140.47538	10140.47572	−0.00034
5	2	4	7	4	2	3	6	10140.71079	10140.71152	−0.00073
6	1	6	8	5	0	5	7	10140.91219	10140.91153	0.00066
5	1	4	4	4	2	3	3	10141.04613	10141.04719	−0.00106
5	2	4	4	4	1	3	3	10141.15619	10141.15657	−0.00038
6	0	6	6	5	1	5	6	10143.39837	10143.39837	0.0000
6	0	6	5	5	1	5	5	10152.85944	10152.86057	−0.00113
3	3	0	2	2	1	1	1	10178.12713	10178.12779	−0.00066
3	3	0	5	2	1	1	4	10186.36969	10186.36959	0.0001
3	3	0	3	2	1	1	2	10193.12638	10193.12542	0.00096
3	3	0	4	2	1	1	3	10202.72882	10202.72864	0.00018
4	2	2	3	3	2	1	2	10215.44697	10215.44744	−0.00047
4	2	2	6	3	2	1	5	10217.93100	10217.92996	0.00104
4	2	2	4	3	2	1	3	10221.30543	10221.30461	0.00082
4	2	2	4	3	2	1	4	10223.58787	10223.58758	0.00029
4	2	2	5	3	2	1	4	10224.08067[1]	10224.07971	0.00096
4	3	2	3	3	2	1	2	10228.21377[1]	10228.21372	0.00005
4	3	2	6	3	2	1	5	10231.33006	10231.32968	0.00038
4	3	2	3	3	2	1	3	10232.61633	10232.61618	0.00015
4	3	2	4	3	2	1	3	10235.95046	10235.95084	−0.00038
4	3	2	4	3	2	1	4	10238.23254	10238.23381	−0.00127
4	3	2	5	3	2	1	4	10239.44458	10239.44453	0.00005
4	3	1	4	3	3	0	3	11538.07136	11538.07093	0.00043
4	3	1	6	3	3	0	5	11545.28040	11545.27894	0.00146
4	3	1	3	3	3	0	2	11550.73213	11550.73246	−0.00033
3	2	1	2	2	1	2	1	11601.23878	11601.24013	−0.00135
3	2	1	2	2	0	2	1	11601.81649	11601.81785	−0.00136
3	2	1	3	2	1	2	2	11605.01439	11605.01348	0.00091
3	2	1	5	2	1	2	4	11605.17145	11605.17121	0.00024
3	2	1	5	2	0	2	4	11605.99705	11605.99686	0.00019

Table S18: Assignment in Pickett notation of experimentally determined transitions of t-2-^{35}ClBzA

J'	K_a'	K_c'	F'	J''	K_a''	K_c''	F''	ν_{obs} / MHz	ν_{calc} / MHz	$\nu_{obs - calc}$ / MHz
3	2	1	4	2	1	2	3	11608.61149	11608.61150	−0.00001
3	2	1	2	2	1	2	2	11609.41452	11609.41594	−0.00142
3	2	1	4	2	0	2	3	11609.92099	11609.92112	−0.00013
3	2	1	3	2	1	2	3	11610.89313	11610.89447	−0.00134
7	0	7	8	6	1	6	8	11687.59373	11687.59564	−0.00191
5	2	3	6	4	3	2	5	11691.36179	11691.36072	0.00107
5	3	3	6	4	3	2	5	11691.90201	11691.90038	0.00163
5	3	3	5	4	3	2	4	11693.00169	11693.00159	0.0001
8	2	7	10	8	1	8	10	11693.39681	11693.39718	−0.00037
5	2	3	7	4	3	2	6	11694.48574	11694.48547	0.00027
5	3	3	7	4	3	2	6	11694.95599	11694.95596	0.00003
5	2	3	4	4	3	2	3	11695.58426	11695.58592	−0.00166
5	3	3	4	4	3	2	3	11696.03836	11696.03861	−0.00025
7	0	7	7	6	1	6	6	11700.17243	11700.17237	0.00006
7	1	7	8	6	0	6	7	11700.51071	11700.50976	0.00095
7	0	7	9	6	1	6	8	11700.94113	11700.94111	0.00002
5	3	3	6	4	2	2	5	11707.26476[1]	11707.26520	−0.00044
5	3	3	5	4	2	2	4	11707.64727[1]	11707.64782	−0.00055
5	2	3	7	4	2	2	6	11707.88536[1]	11707.88519	0.00017
5	3	3	7	4	2	2	6	11708.35604	11708.35568	0.00036
5	3	3	4	4	2	2	3	11708.80504	11708.80489	0.00015
5	3	2	5	4	4	1	4	13066.44063	13066.44162	−0.00099
5	3	2	7	4	4	1	6	13076.64999	13076.64757	0.00242
5	3	2	4	4	4	1	3	13082.28018	13082.28092	−0.00074
5	4	2	6	4	4	1	5	13095.13845	13095.13978	−0.00133
5	4	2	5	4	4	1	4	13100.13284	13100.13387	−0.00103
5	4	2	7	4	4	1	6	13108.21427	13108.21236	0.00191
5	4	2	4	4	4	1	3	13113.04295	13113.04224	0.00071
4	4	0	5	3	3	1	4	13173.90269	13173.90277	−0.00008
4	4	0	4	3	3	1	3	13178.49781	13178.49805	−0.00024
4	4	0	6	3	3	1	5	13181.05408	13181.05348	0.0006
4	4	0	3	3	3	1	2	13185.31556	13185.31512	0.00044
6	2	4	7	5	3	3	6	13258.47024[1]	13258.47018	0.00006
6	3	4	7	5	3	3	6	13258.48334[1]	13258.48364	−0.0003
6	2	4	6	5	3	3	5	13258.93152[1]	13258.93158	−0.00006
6	2	4	7	5	2	3	6	13259.01032[1]	13259.00984	0.00048
6	3	4	7	5	2	3	6	13259.02262[1]	13259.02330	−0.00068
4	4	0	6	3	2	1	5	13361.80761	13361.80725	0.00036

[1]Measured at the COBRA in Hannover.

[1]Measured at the COBRA in Hannover.

Table S18: Assignment in Pickett notation of experimentally determined transitions of t-2-^{35}ClBzA

J'	K_a'	K_c'	F'	J''	K_a''	K_c''	F''	ν_{obs} / MHz	ν_{calc} / MHz	$\nu_{obs - calc}$ / MHz
4	4	0	5	3	2	1	4	13376.67774	13376.67807	−0.00033
5	3	2	4	4	3	1	3	13380.08420	13380.08420	0.0000
5	3	2	7	4	3	1	6	13381.90930	13381.90907	0.00023
5	3	2	5	4	3	1	4	13385.80748	13385.80721	0.00027
5	3	2	6	4	3	1	5	13387.72759	13387.72688	0.00071
5	4	2	4	4	3	1	3	13410.84551	13410.84552	−0.00001
5	4	2	7	4	3	1	6	13413.47432	13413.47386	0.00046
5	4	2	5	4	3	1	4	13419.49940	13419.49946	−0.00006
5	4	2	5	4	3	1	5	13420.81047	13420.81164	−0.00117
5	4	2	6	4	3	1	5	13422.29175	13422.29184	−0.00009
5	4	1	6	4	4	0	5	14569.12859	14569.12910	−0.00051
5	4	1	5	4	4	0	4	14573.56351	14573.56427	−0.00076
5	4	1	7	4	4	0	6	14582.19827	14582.19524	0.00303
5	4	1	4	4	4	0	3	14586.48058	14586.48152	−0.00094
4	3	1	5	3	2	2	4	14662.82573	14662.82373	0.002
4	3	1	4	3	2	2	3	14664.17012	14664.16964	0.00048
4	3	1	6	3	2	2	5	14666.48604	14666.48636	−0.00032
4	3	1	5	3	1	2	4	14668.30404	14668.30413	−0.00009
4	3	1	4	3	1	2	3	14669.09550	14669.09685	−0.00135
4	3	1	6	3	1	2	5	14670.84742	14670.84684	0.00058
4	3	1	3	3	1	2	2	14671.53711	14671.53656	0.00055
6	3	3	7	5	4	2	6	14803.53830	14803.53742	0.00088
6	3	3	6	5	4	2	5	14804.50184	14804.50167	0.00017
6	4	3	7	5	4	2	6	14805.11504	14805.11267	0.00237
6	4	3	6	5	4	2	5	14806.04039	14806.04076	−0.00037
6	3	3	8	5	4	2	7	14806.58222[1]	14806.58227	−0.00005
6	3	3	5	5	4	2	4	14807.52602	14807.52732	−0.0013
6	4	3	8	5	4	2	7	14808.01100	14808.01070	0.0003
6	4	3	5	5	4	2	4	14808.92212	14808.92314	−0.00102
8	2	7	8	7	1	6	7	14819.72047	14819.72162	−0.00115
8	2	7	9	7	1	6	8	14819.75085	14819.75038	0.00047
7	2	5	9	6	3	4	8	14820.38651	14820.38691	−0.0004
7	3	5	9	6	2	4	8	14820.39958	14820.39896	0.00062
8	2	7	7	7	1	6	6	14820.62111	14820.62142	−0.00031
8	2	7	10	7	1	6	9	14820.65729	14820.65539	0.0019
9	1	9	10	8	0	8	9	14820.79649[1]	14820.79605	0.00044
9	1	9	8	8	0	8	7	14820.83990[1]	14820.84014	−0.00024
9	1	9	11	8	0	8	10	14821.06520[1]	14821.06497	0.00023

[1]Measured at the COBRA in Hannover.

Table S18: Assignment in Pickett notation of experimentally determined transitions of t-2-^{35}ClBzA

J'	K_a'	K_c'	F'	J''	K_a''	K_c''	F''	ν_{obs} / MHz	ν_{calc} / MHz	$\nu_{obs - calc}$ / MHz
6	4	3	8	5	3	2	7	14839.57527	14839.57550	−0.00023
6	4	3	7	5	3	2	6	14839.67686	14839.67763	−0.00077
6	4	3	5	5	3	2	4	14839.68593	14839.68446	0.00147
6	4	3	6	5	3	2	5	14839.73154	14839.73301	−0.00147
4	4	1	3	3	1	2	2	14969.34057	14969.33984	0.00073
4	4	1	4	3	1	2	3	14988.46315	14988.46244	0.00071
4	4	1	5	3	1	2	4	14995.45597	14995.45619	−0.00022
5	5	1	4	4	4	0	3	15041.48305	15041.48275	0.0003
5	5	1	7	4	4	0	6	15043.23946	15043.23887	0.00059
5	5	1	5	4	4	0	4	15050.19186	15050.19157	0.00029
5	5	1	6	4	4	0	5	15051.97203	15051.97130	0.00073
6	4	2	7	5	5	1	6	16070.29245	16070.29111	0.00134
6	4	2	6	5	5	1	5	16075.17022	16075.17097	−0.00075
6	4	2	8	5	5	1	7	16087.04737	16087.04726	0.00011
6	4	2	5	5	5	1	4	16091.77962	16091.78252	−0.0029
6	5	2	6	5	5	1	5	16141.47659	16141.47737	−0.00078
6	5	2	8	5	5	1	7	16150.17656	16150.17516	0.0014
6	5	2	5	5	5	1	4	16153.95402	16153.95300	0.00102
5	5	0	4	4	4	1	4	16240.45354	16240.45379	−0.00025
5	5	0	6	4	4	1	5	16248.27483	16248.27477	0.00006
5	5	0	5	4	4	1	4	16251.56172	16251.56179	−0.00007
5	5	0	7	4	4	1	6	16255.34726	16255.34612	0.00114
5	5	0	4	4	4	1	3	16258.50254	16258.50339	−0.00085
5	5	0	6	4	4	1	6	16266.54129	16266.54344	−0.00215
7	3	4	9	6	3	3	8	16381.54240[1]	16381.54303	−0.00063
7	4	4	9	6	3	3	8	16381.58679[1]	16381.58679	0.0000
7	3	4	6	6	3	3	5	16381.91944[1]	16381.92066	−0.00122
7	4	4	6	6	3	3	5	16381.96338[1]	16381.96356	−0.00018
6	4	2	5	5	4	1	4	16546.78164	16546.78376	−0.00212
6	4	2	8	5	4	1	7	16548.08990	16548.09088	−0.00098
6	4	2	6	5	4	1	5	16551.79923	16551.79827	0.00096
6	4	2	7	5	4	1	6	16553.13546	16553.13331	0.00215
5	5	0	4	4	3	1	3	16556.30611	16556.30667	−0.00056
5	5	0	7	4	3	1	6	16560.60636	16560.60763	−0.00127
5	5	0	5	4	3	1	4	16570.92770	16570.92738	0.00032
5	5	0	6	4	3	1	5	16575.42484	16575.42683	−0.00199
6	5	2	5	5	4	1	4	16608.95390	16608.95424	−0.00034
6	5	2	8	5	4	1	7	16611.21887	16611.21878	0.00009
6	5	2	6	5	4	1	5	16618.10489	16618.10468	0.00021
6	5	2	7	5	4	1	6	16620.45366	16620.45405	−0.00039

Table S18: Assignment in Pickett notation of experimentally determined transitions of t-2-^{35}ClBzA

J'	K_a'	K_c'	F'	J''	K_a''	K_c''	F''	ν_{obs} / MHz	ν_{calc} / MHz	$\nu_{obs - calc}$ / MHz
6	5	1	8	5	5	0	7	17597.96173	17597.96047	0.00126
6	5	1	5	5	5	0	4	17601.60005	17601.60031	−0.00026
5	4	1	6	4	3	2	5	17706.36279	17706.36264	0.00015
5	4	1	5	4	3	2	4	17708.41178	17708.41333	−0.00155
5	4	1	7	4	3	2	6	17712.67478	17712.67281	0.00197
5	4	1	4	4	3	2	3	17714.61000	17714.61143	−0.00143
5	4	1	6	4	2	2	5	17721.72595	17721.72746	−0.00151
5	4	1	5	4	2	2	4	17723.05853	17723.05956	−0.00103
5	4	1	7	4	2	2	6	17726.07389	17726.07253	0.00136
5	4	1	4	4	2	2	3	17727.37877	17727.37771	0.00106
5	5	1	4	4	2	2	3	18182.37976[1]	18182.37894	0.00082
5	5	1	7	4	2	2	6	18187.11710[1]	18187.11616	0.00094
5	5	1	5	4	2	2	4	18199.68498[1]	18199.68686	−0.00188
5	5	1	6	4	2	2	5	18204.57075[1]	18204.56966	0.00109
6	6	1	5	5	5	0	4	18244.19394	18244.19371	0.00023
6	6	1	8	5	5	0	7	18245.62376	18245.62386	−0.0001
6	6	1	6	5	5	0	5	18252.39397	18252.39359	0.00038

Table S19: Assignment in Pickett notation of experimentally determined transitions of t-2-^{37}ClBzA

J'	K_a'	K_c'	F'	J''	K_a''	K_c''	F''	ν_{obs} / MHz	ν_{calc} / MHz	$\nu_{obs - calc}$ / MHz
4	1	3	6	3	2	2	5	8442.45143	8442.45108	0.00035
4	1	3	3	3	2	2	2	8443.49195	8443.49199	−0.00004
5	0	5	5	4	1	4	4	8450.61881	8450.61952	−0.00071
5	1	5	5	4	0	4	4	8450.62433	8450.62424	0.00009
5	1	5	4	4	0	4	3	8451.22724	8451.22773	−0.00049
5	0	5	4	4	1	4	3	8451.23169	8451.23216	−0.00047
5	0	5	7	4	1	4	6	8451.69108	8451.69092	0.00016
5	1	5	7	4	0	4	6	8451.69745	8451.69610	0.00135
3	3	1	2	2	2	0	2	8633.18004	8633.18212	−0.00208
3	3	1	2	2	2	0	1	8640.46439	8640.46324	0.00115
3	3	1	5	2	2	0	4	8642.73421	8642.73398	0.00023
3	3	1	3	2	2	0	3	8643.89408	8643.89486	−0.00078
3	3	1	3	2	2	0	2	8649.18386	8649.18319	0.00067
3	3	1	4	2	2	0	3	8651.39436	8651.39457	−0.00021
3	3	1	4	2	2	0	4	8658.77731	8658.77599	0.00132
4	2	2	5	3	3	1	4	9724.93240	9724.93201	0.00039

[1]Measured at the COBRA in Hannover.

Table S19: Assignment in Pickett notation of experimentally determined transitions of t-2-^{37}ClBzA

J'	K_a'	K_c'	F'	J''	K_a''	K_c''	F''	ν_{obs} / MHz	ν_{calc} / MHz	$\nu_{obs - calc}$ / MHz
4	2	2	4	3	3	1	3	9732.83723	9732.83748	−0.00025
4	2	2	6	3	3	1	5	9742.10303	9742.10117	0.00186
4	2	2	3	3	3	1	2	9749.95137	9749.95193	−0.00056
3	3	0	3	2	2	1	3	9905.61964	9905.62016	−0.00052
3	3	0	4	2	2	1	3	9909.69641	9909.69587	0.00054
3	3	0	5	2	2	1	4	9915.14961	9915.14954	0.00007
3	3	0	3	2	2	1	2	9915.75092	9915.75075	0.00017
3	3	0	2	2	2	1	1	9921.21839	9921.21896	−0.00057
5	1	4	6	4	2	3	5	9986.31780[1]	9986.31744	0.00036
5	1	4	5	4	2	3	4	9986.53968[1]	9986.54110	−0.00142
6	0	6	6	5	1	5	5	9987.43948	9987.43873	0.00075
5	2	4	6	4	1	3	5	9987.56570	9987.56601	−0.00031
6	0	6	7	5	1	5	6	9987.78870	9987.78766	0.00104
5	1	4	7	4	2	3	6	9987.81936	9987.81824	0.00112
6	0	6	8	5	1	5	7	9988.22367[1]	9988.22259	0.00108
5	2	4	7	4	1	3	6	9988.95025[1]	9988.94989	0.00036
5	2	4	4	4	1	3	3	9989.13377[1]	9989.13372	0.00005
3	3	0	5	2	1	1	4	10103.10074	10103.10077	−0.00003
4	2	2	6	3	2	1	5	10123.05522	10123.05572	−0.0005
4	2	2	4	3	2	1	3	10125.41349	10125.41282	0.00067
4	3	2	3	3	2	1	2	10178.12697	10178.13414	−0.00717
4	3	2	6	3	2	1	5	10181.81176	10181.81163	0.00013
4	3	2	4	3	2	1	3	10186.70445	10186.70385	0.0006
4	3	2	5	3	2	1	4	10190.39249	10190.39263	−0.00014
4	3	1	5	3	3	0	4	11185.97007[1]	11185.97057	−0.0005
4	3	1	4	3	3	0	3	11191.67195[1]	11191.67170	0.00025
4	3	1	6	3	3	0	5	11199.32935[1]	11199.32790	0.00145
4	3	1	3	3	3	0	2	11204.99644[1]	11204.99828	−0.00184
3	2	1	5	2	1	2	4	11336.68245	11336.68326	−0.00081
3	2	1	4	2	1	2	3	11337.33969	11337.33677	0.00292
5	2	3	6	4	3	2	5	11494.25605	11494.25631	−0.00026
5	2	3	5	4	3	2	4	11495.41749	11495.41763	−0.00014
5	2	3	7	4	3	2	6	11497.65698[1]	11497.65796	−0.00098
5	2	3	4	4	3	2	3	11498.78910[1]	11498.79194	−0.00284
6	1	5	7	5	2	4	6	11523.52760	11523.52855	−0.00095
6	2	5	7	5	1	4	6	11523.57885	11523.58063	−0.00178
6	1	5	6	5	2	4	5	11523.58834	11523.58835	−0.00001
6	2	5	6	5	1	4	5	11523.63884	11523.63893	−0.00009

[1]Measured at the COBRA in Hannover.

Table S19: Assignment in Pickett notation of experimentally determined transitions of t-2-^{37}ClBzA

J'	K_a'	K_c'	F'	J''	K_a''	K_c''	F''	ν_{obs} / MHz	ν_{calc} / MHz	$\nu_{obs - calc}$ / MHz
7	0	7	7	6	1	6	6	11524.19089[1]	11524.18927	0.00162
7	0	7	8	6	1	6	7	11524.46015[1]	11524.46032	−0.00017
7	0	7	6	6	1	6	5	11524.50930[1]	11524.51426	−0.00496
6	1	5	8	5	2	4	7	11524.62956	11524.63142	−0.00186
6	2	5	8	5	1	4	7	11524.67608	11524.67891	−0.00283
6	1	5	5	5	2	4	4	11524.68915	11524.68793	0.00122
6	2	5	5	5	1	4	4	11524.73166	11524.73466	−0.003
7	0	7	9	6	1	6	8	11524.78878	11524.78765	0.00113
5	2	3	4	4	2	2	3	11556.21255	11556.21207	0.00048
5	2	3	7	4	2	2	6	11556.41376	11556.41387	−0.00011
5	2	3	5	4	2	2	4	11556.70915	11556.70866	0.00049
5	2	3	6	4	2	2	5	11556.95131	11556.95365	−0.00234
5	3	3	4	4	2	2	3	11560.50324	11560.50368	−0.00044
5	3	3	7	4	2	2	6	11560.78550	11560.78530	0.0002
5	3	3	5	4	2	2	4	11561.28876	11561.29047	−0.00171
5	3	3	6	4	2	2	5	11561.62041	11561.62147	−0.00106
3	3	1	5	2	0	2	4	11721.46263	11721.46504	−0.00241
3	3	1	3	2	0	2	2	11732.06661	11732.06620	0.00041
3	3	1	4	2	0	2	3	11744.87436	11744.87248	0.00188
4	4	1	3	3	3	0	2	11835.65361	11835.65328	0.00033
4	4	1	6	3	3	0	5	11837.61261	11837.61227	0.00034
4	4	1	4	3	3	0	4	11839.98150	11839.98220	−0.0007
4	4	1	4	3	3	0	3	11844.05851	11844.05791	0.0006
4	4	1	5	3	3	0	4	11845.98041	11845.98007	0.00034
4	4	1	5	3	3	0	5	11854.68578	11854.68532	0.00046
5	3	2	6	4	4	1	5	12593.39599	12593.39636	−0.00037
5	3	2	5	4	4	1	4	12599.80266	12599.80375	−0.00109
5	3	2	7	4	4	1	6	12611.89468	12611.89212	0.00256
5	3	2	4	4	4	1	3	12618.25867	12618.25858	0.00009
5	4	2	5	4	4	1	4	12739.02793	12739.02927	−0.00134
5	4	2	7	4	4	1	6	12746.93752	12746.93676	0.00076
5	4	2	4	4	4	1	3	12751.68313	12751.68430	−0.00117
4	4	0	4	3	3	1	4	12920.57034	12920.57220	−0.00186
4	4	0	5	3	3	1	4	12924.23580	12924.23520	0.0006
4	4	0	4	3	3	1	3	12928.07195	12928.07191	0.00004
4	4	0	6	3	3	1	5	12929.88636	12929.88544	0.00092
4	4	0	3	3	3	1	2	12933.74393	12933.74420	−0.00027
6	2	4	7	5	3	3	6	13058.72383	13058.72498	−0.00115

[1] Measured at the COBRA in Hannover.

Table S19: Assignment in Pickett notation of experimentally determined transitions of t-2-^{37}ClBzA

J'	K_a'	K_c'	F'	J''	K_a''	K_c''	F''	ν_{obs} / MHz	ν_{calc} / MHz	$\nu_{obs - calc}$ / MHz
6	2	4	6	5	3	3	5	13059.05260	13059.05396	−0.00136
8	1	8	8	7	0	7	7	13060.90235	13060.90137	0.00098
8	1	8	9	7	0	7	8	13061.11640[1]	13061.11808	−0.00168
8	1	8	7	7	0	7	6	13061.15565[1]	13061.15445	0.0012
8	0	8	10	7	1	7	9	13061.37513[1]	13061.37324	0.00189
6	3	4	7	5	2	3	6	13063.63682	13063.63827	−0.00145
6	3	4	6	5	2	3	5	13063.87613	13063.87758	−0.00145
6	3	4	8	5	2	3	7	13064.77519	13064.77617	−0.00098
6	3	4	5	5	2	3	4	13065.02014	13065.02094	−0.0008
5	4	2	4	4	3	1	3	13382.34021	13382.33931	0.0009
5	4	2	7	4	3	1	6	13385.22100	13385.22113	−0.00013
5	4	2	5	4	3	1	4	13391.41532	13391.41548	−0.00016
5	4	2	6	4	3	1	5	13394.30238	13394.30277	−0.00039
5	4	1	7	4	4	0	6	14091.82946	14091.82908	0.00038
4	3	1	5	3	2	2	4	14286.83986	14286.83870	0.00116
4	3	1	4	3	2	2	3	14288.49052	14288.49068	−0.00016
4	3	1	6	3	2	2	5	14291.52187	14291.52164	0.00023
6	3	3	7	5	4	2	6	14531.85186[1]	14531.85220	−0.00034
6	3	3	6	5	4	2	5	14532.99221[1]	14532.99257	−0.00036
6	3	3	8	5	4	2	7	14535.78267[1]	14535.78185	0.00082
6	3	3	5	5	4	2	4	14536.89775[1]	14536.89842	−0.00067
8	2	7	8	7	1	6	7	14596.89367	14596.89607	−0.0024
8	2	7	9	7	1	6	8	14596.92798	14596.93019	−0.00221
8	1	7	7	7	2	6	6	14597.57427[1]	14597.57559	−0.00132
9	1	9	9	8	0	8	8	14597.58985[1]	14597.58937	0.00048
7	2	5	7	6	2	4	7	14597.61108[1]	14597.59780	0.01328
6	4	3	5	5	3	2	4	14683.28130	14683.28182	−0.00052
6	4	3	8	5	3	2	7	14683.92705	14683.92733	−0.00028
6	4	3	6	5	3	2	5	14685.79170	14685.79287	−0.00117
6	4	3	7	5	3	2	6	14686.47350	14686.47484	−0.00134
5	5	1	4	4	4	0	3	15048.60383	15048.60288	0.00095
5	5	1	7	4	4	0	6	15050.02465	15050.02374	0.00091
5	5	1	5	4	4	0	4	15056.10812	15056.10692	0.0012
5	5	1	6	4	4	0	5	15057.49068	15057.49026	0.00042
6	4	2	7	5	5	1	6	15379.37159	15379.37171	−0.00012
6	4	2	6	5	5	1	5	15384.94939	15384.94939	0.0000
6	4	2	8	5	5	1	7	15399.45583	15399.45346	0.00237
6	4	2	5	5	5	1	4	15405.00098	15405.00205	−0.00107

[1]Measured at the COBRA in Hannover.

Table S19: Assignment in Pickett notation of experimentally determined transitions of t-2-^{37}ClBzA

J'	K'_a	K'_c	F'	J''	K''_a	K''_c	F''	ν_{obs} / MHz	ν_{calc} / MHz	$\nu_{obs - calc}$ / MHz
5	5	0	6	4	4	1	5	15939.02139	15939.02030	0.00109
5	5	0	5	4	4	1	4	15941.53087	15941.53054	0.00033
5	5	0	7	4	4	1	6	15943.78761	15943.78605	0.00156
5	5	0	4	4	4	1	3	15946.31301	15946.31271	0.0003
9	2	8	8	8	1	7	7	16134.11095	16134.11252	−0.00157
9	2	8	11	8	1	7	10	16134.15861	16134.16190	−0.00329
10	1	10	10	9	0	9	9	16134.26232	16134.26095	0.00137
10	1	10	11	9	0	9	10	16134.40915	16134.40861	0.00054
10	1	10	9	9	0	9	8	16134.42912	16134.42686	0.00226
10	1	10	12	9	0	9	11	16134.57749	16134.57608	0.00141
7	4	4	8	6	3	3	7	16144.89333	16144.89521	−0.00188
7	4	4	7	6	3	3	6	16145.03831	16145.04064	−0.00233
7	4	4	9	6	3	3	8	16145.54343	16145.54468	−0.00125
7	4	4	6	6	3	3	5	16145.69513	16145.69696	−0.00183
6	5	2	5	5	4	1	4	16617.12026	16617.11878	0.00148
6	5	2	8	5	4	1	7	16619.47984	16619.48010	−0.00026
6	5	2	6	5	4	1	5	16626.38576	16626.38556	0.0002
6	5	2	7	5	4	1	6	16628.74765	16628.74880	−0.00115
5	4	1	6	4	3	2	5	17214.36024	17214.35937	0.00087
5	4	1	5	4	3	2	4	17216.39235	17216.39192	0.00043
5	4	1	7	4	3	2	6	17220.85845	17220.85743	0.00102
5	4	1	4	4	3	2	3	17222.86536	17222.86658	−0.00122
7	4	3	8	6	5	2	7	17542.88859	17542.88863	−0.00004
7	4	3	7	6	5	2	6	17544.04857	17544.04903	−0.00046
7	4	3	9	6	5	2	8	17547.64499	17547.64423	0.00076
7	4	3	6	6	5	2	5	17548.78278	17548.78293	−0.00015
10	1	9	10	9	2	8	9	17670.21690	17670.21967	−0.00277
10	2	9	11	9	1	8	10	17670.26749	17670.27044	−0.00295
9	2	7	11	8	3	6	10	17670.55497	17670.55819	−0.00322
9	2	7	8	8	3	6	7	17670.60688	17670.60949	−0.00261
10	2	9	9	9	1	8	8	17670.67413	17670.67774	−0.00361
10	2	9	12	9	1	8	11	17670.72843	17670.73110	−0.00267
11	1	11	13	10	0	10	12	17671.18683	17671.18510	0.00173
8	4	5	9	7	3	4	8	17671.81058	17671.81287	−0.00229
8	3	5	10	7	4	4	9	17671.88030	17671.88255	−0.00225
8	4	5	8	7	3	4	7	17671.97610	17671.97937	−0.00327
8	3	5	7	7	4	4	6	17672.05424	17672.05799	−0.00375
8	4	5	10	7	3	4	9	17672.77039	17672.77289	−0.0025
8	4	5	7	7	3	4	6	17672.93643	17672.94003	−0.0036
7	5	3	7	6	4	2	6	17845.22305	17845.22422	−0.00117

Table S19: Assignment in Pickett notation of experimentally determined transitions of t-2-^{37}ClBzA

J'	K'_a	K'_c	F'	J''	K''_a	K''_c	F''	ν_{obs} / MHz	ν_{calc} / MHz	$\nu_{obs - calc}$ / MHz
7	5	3	8	6	4	2	7	17846.16110	17846.16357	−0.00247

Table S20: Assignment in Pickett notation of experimentally determined transitions of $^{13}C_1$ t-2-ClBzA

J'	K'_a	K'_c	F'	J''	K''_a	K''_c	F''	ν_{obs} / MHz	ν_{calc} / MHz	$\nu_{obs - calc}$ / MHz
5	0	5	7	4	1	4	6	8530.46391	8530.47199	−0.00808
5	1	4	6	4	2	3	5	10079.04507	10079.04900	−0.00393
5	1	4	5	4	2	3	4	10079.41444	10079.40651	0.00793
6	0	6	6	5	0	5	5	10080.26740	10080.25853	0.00887
6	0	6	7	5	1	5	6	10080.69360	10080.69576	−0.00216
6	1	6	5	5	0	5	4	10080.84582	10080.84327	0.00255
6	1	6	8	5	0	5	7	10081.27776	10081.28461	−0.00685
6	1	5	7	5	2	4	6	11630.34591	11630.34177	0.00414
6	1	5	6	5	2	4	5	11630.47437	11630.46525	0.00912
7	0	7	7	6	1	6	6	11631.35786	11631.35351	0.00435
7	1	7	8	6	0	6	7	11631.69405	11631.69532	−0.00127
7	1	7	6	6	0	6	5	11631.79331	11631.79168	0.00163
6	1	5	8	5	2	4	7	11631.85440	11631.85891	−0.00451
6	1	5	5	5	2	4	4	11631.97824	11631.97738	0.00086
7	1	7	9	6	0	6	8	11632.13188	11632.13761	−0.00573
5	3	3	6	4	2	2	5	11632.40773	11632.40048	0.00725
5	3	3	5	4	2	2	4	11632.95693	11632.96436	−0.00743
6	2	4	7	5	3	3	6	13180.40090	13180.40736	−0.00646
7	2	6	8	6	2	5	7	13181.48931	13181.49892	−0.00961
7	2	6	7	6	1	5	6	13181.51936	13181.52002	−0.00066
6	2	4	8	5	3	3	7	13182.35753	13182.35605	0.00148
8	1	8	8	7	0	7	7	13182.40117	13182.39602	0.00515
8	1	8	9	7	0	7	8	13182.66999	13182.67043	−0.00044
8	1	8	7	7	0	7	6	13182.73819	13182.73666	0.00153
8	1	8	10	7	0	7	9	13183.00978	13183.01477	−0.00499
7	2	5	8	6	3	4	7	14731.58152	14731.58461	−0.00309
7	2	5	7	6	3	4	6	14731.86763	14731.86082	0.00681
8	2	7	8	7	1	6	7	14732.54538	14732.55556	−0.01018
8	2	7	9	7	1	6	8	14732.57292	14732.58160	−0.00868
7	2	5	9	6	3	4	8	14733.17377	14733.17338	0.00039
9	0	9	9	8	0	8	8	14733.41335	14733.40579	0.00756
8	2	7	7	7	1	6	6	14733.49488	14733.47883	0.01605
9	1	9	10	8	0	8	9	14733.63013	14733.63087	−0.00074

Table S20: Assignment in Pickett notation of experimentally determined transitions of $^{13}C_1$ t-2-ClBzA

J'	K_a'	K_c'	F'	J''	K_a''	K_c''	F''	ν_{obs} / MHz	ν_{calc} / MHz	$\nu_{obs - calc}$ / MHz
9	1	9	8	8	0	8	7	14733.67439	14733.67822	−0.00383
9	1	9	11	8	0	8	10	14733.90399	14733.90652	−0.00253

7 NQCC calcultation

7.1 2-ClBzA

Table S21: Nuclear quadrupole coupling constants of 2-t-ClBzA determined experimentally and computationally.

calculation	$3/2\chi_{aa}$ / MHz	$1/4\chi_{bb-cc}$ / MHz	χ_{ab} / MHz
B2PLYP-D3/def2-TZVPP	−111.995311	1.17626972	−16.1281253
DKH-B2PLYP-D3/x2c-TZVPPall-2c	−110.349976	0.85874906	−18.6875342
DKH-B2PLYP-D3/x2c-TZVPPall-s	−111.042749	0.91648009	−18.5586016
B2PLYP-D4/def2-TZVPP	−112.471592	1.25757426	−14.9330929
B3LYP-D3/def2-TZVPP	−109.042368	1.33743551	−18.9184583
DKH-B3LYP-D3/x2c-TZVPPall-2c	−107.15745	0.88376584	−22.0070684
DKH-B3LYP-D3/x2c-TZVPPall-s	−107.830017	0.94967543	−21.8242535
B3LYP-D4/def2-TZVPP	−109.628338	1.44760556	−17.6060396
B3LYP/def2-TZVPP	−111.721088	1.72808215	−13.0953218
DKH-B3LYP/x2c-TZVPPall-2c	−110.381728	1.36870649	−16.1492933
DKH-B3LYP/x2c-TZVPPall-s	−111.00811	1.42595643	−15.9607053
B97-D3/def2-TZVPP	−107.307551	1.81804633	−15.4584453
DKH-B97-D3/x2c-TZVPPall-2c	−105.613145	1.38843126	−19.1089707
DKH-B97-D3/x2c-TZVPPall-s	−106.383854	1.4808009	−18.5104924
B97-D4/def2-TZVPP	−102.931539	0.59078087	−25.6672157
B97M-D3BJ/def2-TZVPP	−106.652304	1.92340546	−12.6873559
DKH-B97M-D3BJ/x2c-TZVPPall-2c	−104.016882	1.63715578	−15.1332272
DKH-B97M-D3BJ/x2c-TZVPPall-s	−105.15707	1.70980065	−14.6232698
B97M-D4/def2-TZVPP	−108.984637	2.24573704	−2.19762785
BP86-D3/def2-TZVPP	−105.685309	1.60732808	−18.0351736
DKH-BP86-D3/x2c-TZVPPall-2c	−103.996676	1.18829702	−20.9794561
DKH-BP86-D3/x2c-TZVPPall-s	−104.781818	1.27970449	−20.5002885
BP86-D4/def2-TZVPP	−106.444472	1.73722289	−16.3917636
MP2/6-311++G(2d,2p)	−106.242413	1.22197346	−10.0278798
MP2/6-311++G(d,p)	−104.048634	1.10747358	−11.9868861
experimental ^{35}Cl	−89.970(2)	−1.3814(6)	−34.30(2)
experimental ^{37}Cl	−84.882(1)	1.2399(6)	−1.8(6)

Table S22: Nuclear quadrupole coupling constants of 2-t-ClBzA determined experimentally and computationally.

calculation	χ_{xx} / MHz	χ_{yy} / MHz	χ_{zz} / MHz
B2PLYP-D3/def2-TZVPP	34.9792307	41.9155538	−76.8947845
DKH-B2PLYP-D3/x2c-TZVPPall-2c	35.0658273	41.5348748	−76.6007021
DKH-B2PLYP-D3/x2c-TZVPPall-s	35.1812893	41.820239	−77.0015283

Table S22: Nuclear quadrupole coupling constants of 2-t-ClBzA determined experimentally and computationally.

calculation	χ_{xx} / MHz	χ_{yy} / MHz	χ_{zz} / MHz
B2PLYP-D4/def2-TZVPP	34.975382	41.91336	−76.888742
B3LYP-D3/def2-TZVPP	33.6725851	42.1390706	−75.8116557
DKH-B3LYP-D3/x2c-TZVPPall-2c	33.9516184	41.7649261	−75.7165445
DKH-B3LYP-D3/x2c-TZVPPall-s	34.043988	42.0240183	−76.0680063
B3LYP-D4/def2-TZVPP	33.6475683	42.1283992	−75.7759675
B3LYP/def2-TZVPP	33.7841984	42.1666624	−75.9508608
DKH-B3LYP/x2c-TZVPPall-2c	34.0555343	41.7907426	−75.8482013
DKH-B3LYP/x2c-TZVPPall-s	34.1498283	42.0487001	−76.2004527
B97-D3/def2-TZVPP	32.133091	41.5189262	−73.6520171
DKH-B97-D3/x2c-TZVPPall-2c	32.4275192	41.2514577	−73.678977
DKH-B97-D3/x2c-TZVPPall-s	32.5006452	41.4723698	−73.9710906
B97-D4/def2-TZVPP	33.1299134	41.4768573	−74.6048463
B97M-D3BJ/def2-TZVPP	31.703957	40.8356091	−72.5395661
DKH-B97M-D3BJ/x2c-TZVPPall-2c	31.3979825	40.0402645	−71.438247
DKH-B97M-D3BJ/x2c-TZVPPall-s	31.6327554	40.4069574	−72.0397128
B97M-D4/def2-TZVPP	31.8367384	40.8622308	−72.6989692
BP86-D3/def2-TZVPP	32.0137802	41.352225	−73.3660052
DKH-BP86-D3/x2c-TZVPPall-2c	32.2889648	41.0303	−73.3192648
DKH-BP86-D3/x2c-TZVPPall-s	32.3678638	41.2686277	−73.6364915
BP86-D4/def2-TZVPP	32.0080071	41.3492465	−73.3553292
MP2/6-311++G(2d,2p)	32.9701909	38.7755561	−71.745747
MP2/6-311++G(d,p)	32.4679317	38.2332048	−70.7011366
experimental ^{35}Cl	39.101	32.7528	−71.85
experimental ^{37}Cl	30.821	25.8080	−56.63

7.2 3-ClBzA

Table S23: Nuclear quadrupole coupling constants of 3-t-ClBzA determined experimentally and computationally.

calculation	$3/2\chi_{aa}$ / MHz	$1/4\chi_{bb-cc}$ / MHz	χ_{ab} / MHz
B2PLYP-D3/def2-TZVPP	−84.0073075	−3.90598523	45.1726061
DKH-B2PLYP-D3/x2c-TZVPPall-2c	−83.8081354	−3.98921413	45.0840852
DKH-B2PLYP-D3/x2c-TZVPPall-s	−84.2064795	−3.98921413	45.334253
B2PLYP-D4/def2-TZVPP	−83.9726688	−3.9079096	45.182228
B3LYP-D3/def2-TZVPP	−82.9825817	−3.46674832	44.9282115
DKH-B3LYP-D3/x2c-TZVPPall-2c	−82.9594893	−3.59375658	44.8974216
DKH-B3LYP-D3/x2c-TZVPPall-s	−83.3231948	−3.58702129	45.1110264
B3LYP-D4/def2-TZVPP	−82.8988717	−3.46722941	44.9339846

Table S23: Nuclear quadrupole coupling constants of 3-t-ClBzA determined experimentally and computationally.

calculation	$3/2\chi_{aa}$ / MHz	$1/4\chi_{bb-cc}$ / MHz	χ_{ab} / MHz
B3LYP/def2-TZVPP	−83.0374262	−3.5033113	45.0455979
DKH-B3LYP/x2c-TZVPPall-2c	−83.0027875	−3.62983847	45.014808
DKH-B3LYP/x2c-TZVPPall-s	−83.366493	−3.62358428	45.2284128
B97-D3/def2-TZVPP	−80.5145802	−3.12661633	44.0526242
DKH-B97-D3/x2c-TZVPPall-2c	−80.5780843	−3.25170023	44.1084308
DKH-B97-D3/x2c-TZVPPall-s	−80.8782857	−3.24496494	44.287397
B97-D4/def2-TZVPP	−81.6259025	−3.39939545	44.3278088
B97M-D3BJ/def2-TZVPP	−79.5475854	−3.13238944	43.0442555
DKH-B97M-D3BJ/x2c-TZVPPall-2c	−78.3640993	−3.16750915	42.4630965
DKH-B97M-D3BJ/x2c-TZVPPall-s	−79.0106869	−3.18627173	42.8210289
B97M-D4/def2-TZVPP	−79.6110896	−3.17232007	43.1751125
BP86-D3/def2-TZVPP	−80.5059205	−3.04627399	43.8755824
DKH-BP86-D3/x2c-TZVPPall-2c	−80.477055	−3.17520662	43.8871286
DKH-BP86-D3/x2c-TZVPPall-s	−80.8061219	−3.16750915	44.0853384
BP86-D4/def2-TZVPP	−80.4481895	−3.05060381	43.8929017
MP2/6-311++G(2d,2p)	−78.2832759	−3.77272277	41.5143833
MP2/6-311++G(d,p)	−76.5946433	−3.83093489	41.2180306
experimental ^{35}Cl	−78.548(2)	−3.5901(5)	42.34(1)
experimental ^{37}Cl	−62.71(1)	−2.694(2)	32.90(1)

Table S24: Nuclear quadrupole coupling constants of 3-t-ClBzA determined experimentally and computationally.

calculation	χ_{xx} / MHz	χ_{yy} / MHz	χ_{zz} / MHz
B2PLYP-D3/def2-TZVPP	35.8144062	41.1859158	−77.000322
DKH-B2PLYP-D3/x2c-TZVPPall-2c	35.9144734	40.9504102	−76.8648836
DKH-B2PLYP-D3/x2c-TZVPPall-s	36.0472548	41.2036758	−77.2509306
B2PLYP-D4/def2-TZVPP	35.8067088	41.1847168	−76.9914256
B3LYP-D3/def2-TZVPP	34.5943572	41.5620604	−76.1564176
DKH-B3LYP-D3/x2c-TZVPPall-2c	34.8406763	41.3259516	−76.1666278
DKH-B3LYP-D3/x2c-TZVPPall-s	34.9484408	41.5569412	−76.5053821
B3LYP-D4/def2-TZVPP	34.567416	41.5525986	−76.1200146
B3LYP/def2-TZVPP	34.6867268	41.6009041	−76.2857065
DKH-B3LYP/x2c-TZVPPall-2c	34.9272728	41.3631232	−76.290396
DKH-B3LYP/x2c-TZVPPall-s	35.0369618	41.5941031	−76.6291405
B97-D3/def2-TZVPP	33.0914261	41.0678325	−74.1592586
DKH-B97-D3/x2c-TZVPPall-2c	33.3627619	40.9147853	−74.2775472
DKH-B97-D3/x2c-TZVPPall-s	33.4493584	41.1093973	−74.5587557
B97-D4/def2-TZVPP	34.0074251	41.0025698	−75.0099948

Table S24: Nuclear quadrupole coupling constants of 3-t-ClBzA determined experimentally and computationally.

calculation	χ_{xx} / MHz	χ_{yy} / MHz	χ_{zz} / MHz
B97M-D3BJ/def2-TZVPP	32.7796785	40.1367592	−72.9183621
DKH-B97M-D3BJ/x2c-TZVPPall-2c	32.4563847	39.4509033	−71.907288
DKH-B97M-D3BJ/x2c-TZVPPall-s	32.7104013	39.7951198	−72.5035967
B97M-D4/def2-TZVPP	32.88167	40.181453	−73.063123
BP86-D3/def2-TZVPP	32.9278548	41.0634941	−73.991349
DKH-BP86-D3/x2c-TZVPPall-2c	33.1760982	40.8555737	−74.031672
DKH-BP86-D3/x2c-TZVPPall-s	33.2703923	41.0709854	−74.3413776
BP86-D4/def2-TZVPP	32.918233	41.0613295	−73.9776381
MP2/6-311++G(2d,2p)	33.6398708	37.718163	−71.3580338
MP2/6-311++G(d,p)	33.1934175	37.1327791	−70.3261966
experimental ^{35}Cl	38.690	33.3629	−72.05
experimental ^{37}Cl	30.49	26.291	−56.78

Table S25: Nuclear quadrupole coupling constants of 3-c-ClBzA determined experimentally and computationally.

calculation	$3/2\chi_{aa}$ / MHz	$1/4\chi_{bb-cc}$ / MHz	χ_{ab} / MHz
B2PLYP-D3/def2-TZVPP	−60.48191297	−7.681113463	54.8271586
DKH-B2PLYP-D3/x2c-TZVPPall-2c	−60.31160643	−7.76338018	54.6866798
DKH-B2PLYP-D3/x2c-TZVPPall-s	−60.58871537	−7.782142765	54.9907299
B2PLYP-D4/def2-TZVPP	−60.4443878	−7.684000015	54.8310074
B3LYP-D3/def2-TZVPP	−59.63615338	−7.214935401	54.546201
DKH-B3LYP-D3/x2c-TZVPPall-2c	−59.64481304	−7.33809493	54.4749994
DKH-B3LYP-D3/x2c-TZVPPall-s	−59.90460267	−7.345792401	54.7367134
B3LYP-D4/def2-TZVPP	−59.54089719	−7.217340861	54.5404279
B3LYP/def2-TZVPP	−59.73429613	−7.246206375	54.6308731
DKH-B3LYP/x2c-TZVPPall-2c	−59.72563648	−7.369846997	54.5596715
DKH-B3LYP/x2c-TZVPPall-s	−59.98542611	−7.378506651	54.8213855
B97-D3/def2-TZVPP	−57.54629012	−6.803601816	53.4242946
DKH-B97-D3/x2c-TZVPPall-2c	−57.63288666	−6.928204622	53.4416139
DKH-B97-D3/x2c-TZVPPall-s	−57.84071837	−6.935421	53.6609918
B97-D4/def2-TZVPP	−58.56524279	−7.100435526	53.7841514
B97M-D3BJ/def2-TZVPP	−56.84197156	−6.771368658	52.3716655
DKH-B97M-D3BJ/x2c-TZVPPall-2c	−56.02219094	−6.750681706	51.6211621
DKH-B97M-D3BJ/x2c-TZVPPall-s	−56.48115263	−6.799271989	52.0579936
B97M-D4/def2-TZVPP	−56.94588741	−6.806488368	52.4659595
BP86-D3/def2-TZVPP	−57.44526082	−6.727108202	53.3223031
DKH-BP86-D3/x2c-TZVPPall-2c	−57.47701288	−6.850748824	53.2780427
DKH-BP86-D3/x2c-TZVPPall-s	−57.71082355	−6.857003019	53.5166643

Table S25: Nuclear quadrupole coupling constants of 3-c-ClBzA determined experimentally and computationally.

calculation	$3/2\chi_{aa}$ / MHz	$1/4\chi_{bb-cc}$ / MHz	χ_{ab} / MHz
BP86-D4/def2-TZVPP	−57.37309703	−6.733843489	53.3300006
MP2/6-311++G(2d,2p)	−56.28198058	−7.328954184	50.6397346
MP2/6-311++G(d,p)	−54.58757486	−7.399193603	50.1317016
experimental ^{35}Cl	−56.075(1)	−7.2225(4)	51.50(2)
experimental ^{37}Cl	−45.949(3)	−5.4002(5)	40.10(3)

Table S26: Nuclear quadrupole coupling constants of 3-c-ClBzA determined experimentally and computationally.

calculation	χ_{xx} / MHz	χ_{yy} / MHz	χ_{zz} / MHz
B2PLYP-D3/def2-TZVPP	35.5238268	41.5263624	−77.0482647
DKH-B2PLYP-D3/x2c-TZVPPall-2c	35.629667	41.277622	−76.9092133
DKH-B2PLYP-D3/x2c-TZVPPall-s	35.760524	41.5401065	−77.3006305
B2PLYP-D4/def2-TZVPP	35.5161293	41.524391	−77.0405203
B3LYP-D3/def2-TZVPP	34.3095508	41.8903379	−76.1979643
DKH-B3LYP-D3/x2c-TZVPPall-2c	34.5577942	41.6538597	−76.2116539
DKH-B3LYP-D3/x2c-TZVPPall-s	34.6597857	41.8914043	−76.55119
B3LYP-D4/def2-TZVPP	34.2806853	41.8780076	−76.1606172
B3LYP/def2-TZVPP	34.4038448	41.9270832	−76.330928
DKH-B3LYP/x2c-TZVPPall-2c	34.6482395	41.6902134	−76.3384529
DKH-B3LYP/x2c-TZVPPall-s	34.7521553	41.9264235	−76.6785788
B97-D3/def2-TZVPP	32.7893003	41.3705304	−74.1598307
DKH-B97-D3/x2c-TZVPPall-2c	33.0683336	41.2173118	−74.2837211
DKH-B97-D3/x2c-TZVPPall-s	33.1510815	41.4144545	−74.565536
B97-D4/def2-TZVPP	33.7226186	41.3175898	−75.0402085
B97M-D3BJ/def2-TZVPP	32.4910234	40.4257082	−72.9148071
DKH-B97M-D3BJ/x2c-TZVPPall-2c	32.1754271	39.7401431	−71.9155701
DKH-B97M-D3BJ/x2c-TZVPPall-s	32.4255949	40.0878618	−72.5134566
B97M-D4/def2-TZVPP	32.5949392	40.4661825	−73.0611217
BP86-D3/def2-TZVPP	32.6026367	41.3794498	−73.9820864
DKH-BP86-D3/x2c-TZVPPall-2c	32.8605019	41.1685664	−74.0290684
DKH-BP86-D3/x2c-TZVPPall-s	32.9509472	41.3861142	−74.3370614
BP86-D4/def2-TZVPP	32.5930148	41.3762099	−73.9673004
MP2/6-311++G(2d,2p)	33.4185686	38.0403602	−71.4589288
MP2/6-311++G(d,p)	32.9932833	37.4372015	−70.4324092
experimental ^{35}Cl	38.979	33.1366	−72.12
experimental ^{37}Cl	30.724	26.1167	−56.84

7.3 4-ClBzA

Table S27: Nuclear quadrupole coupling constants of 4-ClBzA determined experimentally and computationally.

calculation	$3/2\chi_{aa}$ / MHz	$1/4\chi_{bb-cc}$ / MHz	χ_{ab} / MHz
B2PLYP-D3/def2-TZVPP	−113.256734	1.673718761	−10.3203837
DKH-B2PLYP-D3/x2c-TZVPPall-2c	−112.942099	1.596744055	−10.2838207
DKH-B2PLYP-D3/x2c-TZVPPall-s	−113.513637	1.628977213	10.358871
B2PLYP-D4/def2-TZVPP	−113.245187	1.674199853	−10.3203837
B3LYP-D3/def2-TZVPP	−111.749954	2.160583776	−9.89702281
DKH-B3LYP-D3/x2c-TZVPPall-2c	−111.683563	2.048970452	−9.85661109
DKH-B3LYP-D3/x2c-TZVPPall-s	−112.197369	2.08360907	−9.86815729
B3LYP-D4/def2-TZVPP	−111.697996	2.163951419	−9.89894718
B3LYP/def2-TZVPP	−111.960672	2.146151018	−9.86815729
DKH-B3LYP/x2c-TZVPPall-2c	−111.888508	2.035499879	−9.82582121
DKH-B3LYP/x2c-TZVPPall-s	−112.402314	2.069657404	−9.83544304
B97-D3/def2-TZVPP	−108.462172	2.494461563	−9.79888006
DKH-B97-D3/x2c-TZVPPall-2c	−108.566087	2.40064864	−9.77963638
DKH-B97-D3/x2c-TZVPPall-s	−108.981751	2.431919614	−9.81235063
B97-D4/def2-TZVPP	−110.032456	2.152886305	−9.7546196
B97M-D3BJ/def2-TZVPP	−106.678283	2.293846235	−9.76809018
DKH-B97M-D3BJ/x2c-TZVPPall-2c	−105.119545	2.213503886	−9.61029203
DKH-B97M-D3BJ/x2c-TZVPPall-s	−105.999943	2.242850493	−9.67572053
B97M-D4/def2-TZVPP	−106.917866	2.277008018	−9.7546196
BP86-D3/def2-TZVPP	−108.112899	2.545938397	−9.90087154
DKH-BP86-D3/x2c-TZVPPall-2c	−108.104239	2.44346582	−9.86045982
DKH-BP86-D3/x2c-TZVPPall-s	−108.557428	2.477623346	−9.89509844
BP86-D4/def2-TZVPP	−108.098466	2.547381673	−9.90087154
MP2/6-311++G(2d,2p)	−105.613145	1.297985978	−9.50060307
MP2/6-311++G(d,p)	−104.166983	1.260460809	−9.38706538
experimental ^{35}Cl	−106.044(2)	1.497(1)	−9.5(1)
experimental ^{37}Cl	−83.605(3)	1.186(1)	−7.3(4)

Table S28: Nuclear quadrupole coupling constants of 4-ClBzA determined experimentally and computationally.

calculation	χ_{xx} / MHz	χ_{yy} / MHz	χ_{zz} / MHz
B2PLYP-D3/def2-TZVPP	34.4038448	42.0051165	−76.4108857
DKH-B2PLYP-D3/x2c-TZVPPall-2c	34.4538784	41.7444576	−76.198336
DKH-B2PLYP-D3/x2c-TZVPPall-s	34.5808866	42.0086091	−76.5875713
B2PLYP-D4/def2-TZVPP	34.3999961	42.0032658	−76.4032619
B3LYP-D3/def2-TZVPP	32.9278548	42.4080372	−75.3378164
DKH-B3LYP-D3/x2c-TZVPPall-2c	33.1299134	42.1589051	−75.2888185
DKH-B3LYP-D3/x2c-TZVPPall-s	33.2319049	42.3972649	−75.6291698

Table S28: Nuclear quadrupole coupling constants of 4-ClBzA determined experimentally and computationally.

calculation	χ_{xx} / MHz	χ_{yy} / MHz	χ_{zz} / MHz
B3LYP-D4/def2-TZVPP	32.9047624	42.3990541	−75.3038165
B3LYP/def2-TZVPP	33.0279219	42.4442362	−75.4721581
DKH-B3LYP/x2c-TZVPPall-2c	33.2242075	42.1929103	−75.4190422
DKH-B3LYP/x2c-TZVPPall-s	33.3281233	42.4309785	−75.7591018
B97-D3/def2-TZVPP	31.165134	41.9830978	−73.1482319
DKH-B97-D3/x2c-TZVPPall-2c	31.3864363	41.8264918	−73.2148525
DKH-B97-D3/x2c-TZVPPall-s	31.4634111	42.0306249	−73.494036
B97-D4/def2-TZVPP	32.3717126	41.8094901	−74.1812027
B97M-D3BJ/def2-TZVPP	30.9707729	40.9972009	−71.9698981
DKH-B97M-D3BJ/x2c-TZVPPall-2c	30.6128405	40.3035565	−70.916397
DKH-B97M-D3BJ/x2c-TZVPPall-s	30.8476134	40.6599606	−71.507574
B97M-D4/def2-TZVPP	31.0843106	41.0395134	−72.1257484
BP86-D3/def2-TZVPP	30.9457561	41.9889141	−72.9346702
DKH-BP86-D3/x2c-TZVPPall-2c	31.1478147	41.7757207	−72.9235354
DKH-BP86-D3/x2c-TZVPPall-s	31.2305625	41.9971721	−73.2277347
BP86-D4/def2-TZVPP	30.9380587	41.9870761	−72.9251348
MP2/6-311++G(2d,2p)	32.6084098	38.6281601	−71.2365698
MP2/6-311++G(d,p)	32.2023718	38.0638397	−70.2642872
experimental ^{35}Cl	39.164	32.354	−71.51
experimental ^{37}Cl	30.807	25.496	−56.30

8 Raman Spectra

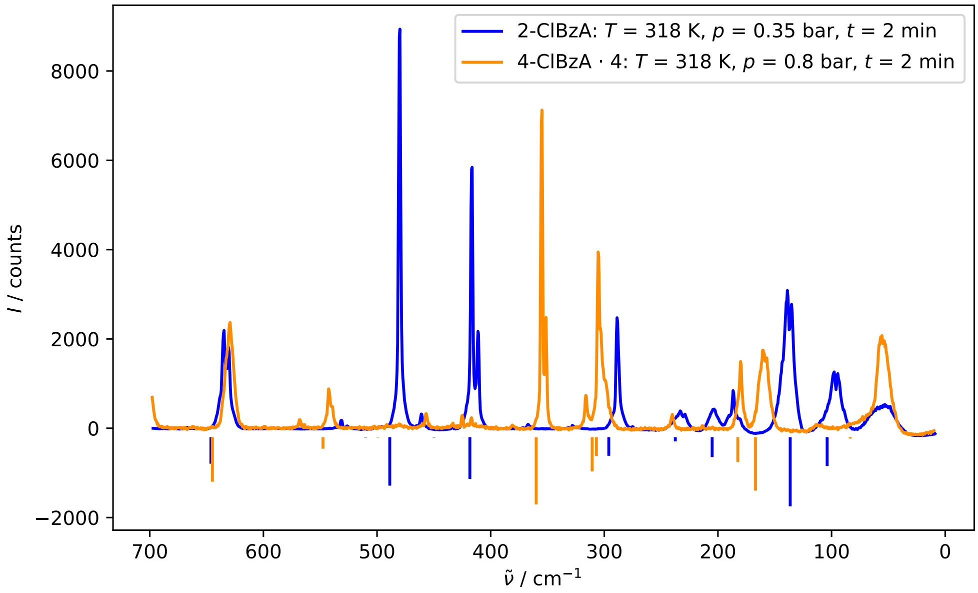

Figure S5: Raman spectra of 2-ClBzA and 4-ClBzA recorded at the Curry setup (`https://doi.org/10.6084/m9.figshare.6395840.v1`) recorded in helium. The 4-ClBzA spectrum is scaled Below is the the harmonic predictions at B3LYP-D3(BJ, abc)/ma-def2-TZVP level of theory.
